# Boronic acid–DMAPO cooperative catalysis for dehydrative condensation between carboxylic acids and amines†
†Electronic supplementary information (ESI) available: Experimental procedure and characterization data of new compounds are provided. CCDC 1429213. For ESI and crystallographic data in CIF or other electronic format see DOI: 10.1039/c5sc03761a


**DOI:** 10.1039/c5sc03761a

**Published:** 2015-11-06

**Authors:** Kazuaki Ishihara, Yanhui Lu

**Affiliations:** a Graduate School of Engineering , Nagoya University , B2-3(611), Furo-cho, Chikusa , Nagoya 464-8603 , Japan . Email: ishihara@cc.nagoya-u.ac.jp; b JST , CREST , B2-3(611), Furo-cho, Chikusa , Nagoya 464-8603 , Japan

## Abstract

Arylboronic acid and 4-(*N*,*N*-dimethylamino)pyridine *N*-oxide (DMAPO) cooperatively catalyse the dehydrative condensation reaction between carboxylic acids and amines to give the corresponding amides under azeotropic reflux conditions. This cooperative use is much more effective than their individual use as catalysts, and chemoselectively promotes the amide condensation of (poly)conjugated carboxylic acids. The present method is practical and scalable, and has been applied to the synthesis of sitagliptin and a drug candidate.

## Abstract 2

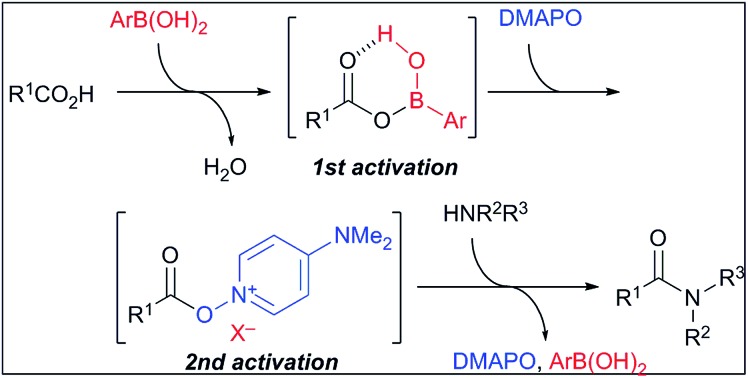
Arylboronic acid and DMAPO cooperatively catalyse the dehydrative condensation reaction between carboxylic acids and amines to give the corresponding amides under azeotropic reflux conditions. This cooperative use is much more effective than their individual use as catalysts.

## Introduction

**Scheme 1:**
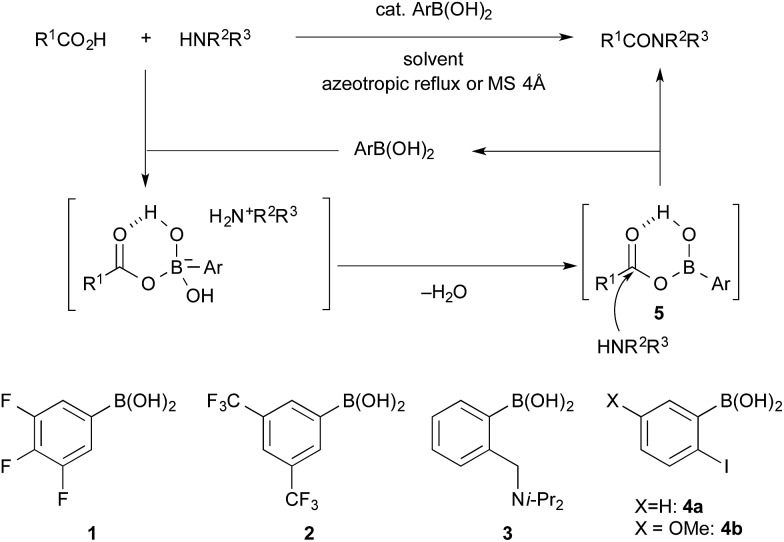
Dehydrative condensation of carboxylic acids with amines catalysed by arylboronic acids, and representative examples of catalysts.

The catalytic dehydrative condensation reaction between carboxylic acids and amines is one of the most ideal methods for synthesizing the corresponding amides.^1^ In 1996, Yamamoto *et al.* reported the first example of the dehydrative amide condensation reaction catalysed by *meta*- or *para*-electron-deficient group-substituted phenylboronic acids such as 3,4,5-trifluorophenylboronic acid (**1**) (p*K*_a_ = 6.8)2*a* and 3,5-bis(trifluoromethyl)phenylboronic acid (**2**) (p*K*_a_ = 7.2)2*b* under azeotropic reflux conditions (Scheme 1).^3^ These boronic acids are more acidic than phenylboronic acid (p*K*_a_ = 8.8, 8.9).^2^

In 2006, Whiting *et al.* reported that *ortho*-Brønsted base-substituted phenylboronic acids such as 2-(*N*,*N*-diisopropylaminomethyl)phenylboronic acid (**3**) were effective catalysts for the amide condensation of aromatic carboxylic acids under the same conditions as above.1a,^4^ In 2008 and 2012, Hall *et al.* reported that 2-iodophenylboronic acid (**4a**) and 2-iodo-5-methoxyphenylboronic acid (**4b**) were also effective catalysts for the amide condensation in the presence of drying agents (activated 4 Å molecular sieves) at lower temperature.^5^ The *o*-iodo group of **4a** and **4b** assists the catalysis of amide condensation as a weak base.^6^ In addition to these boronic acids, boric acid,7a,c benzo[1,3,2]dioxaborol-2-ol,7*b* methylboronic acid,7*d* and some *o*-Brønsted base-substituted boronic acids^8^ have been reported to be useful as amidation catalysts. However, the substrate scope is still quite limited. For example, harsh conditions (higher temperature, prolonged reaction time, excess amounts of substrates, increased amounts of catalysts, *etc.*) are required for sterically hindered α-branched carboxylic acids and conjugated carboxylic acids. In 2013, Whiting *et al.* discovered an interesting synergistic catalytic effect between *o*-tolylboronic acid (50 mol%) and *o*-nitrophenylboronic acid (50 mol%) in dipeptide synthesis.3*f* To the best of our knowledge, this was the first example of two cooperative promoters for direct amidation.3f,^9^

**Scheme 2:**
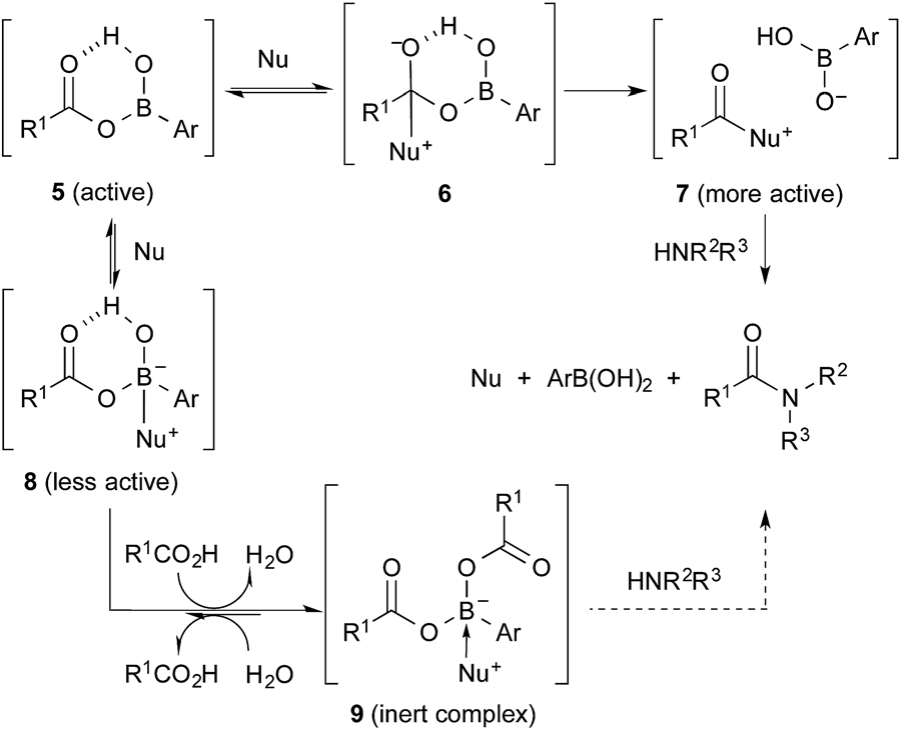
Our proposal: the second activation of a mixed anhydride **5** with a nucleophilic additive (Nu) to generate a more active cationic intermediate **7**.

In the process catalysed by arylboronic acid, a mixed anhydride intermediate **5** is generated from the carboxylic acid and arylboronic acid under azeotropic reflux conditions or in the presence of drying agents in the first stage (Schemes 1 and 2). This is the first activation of the carboxylic acid. If a nucleophilic additive (Nu) reacts with **5** to generate a more active cationic intermediate **7** (ref. 10) (second activation) *via* a tetrahedral intermediate **6**, the amide condensation may proceed more rapidly. However, if Nu preferentially coordinates as a Lewis base to the boron atom of **5**, a less active species **8** is generated and the amide condensation may be suppressed. Here we report that arylboronic acids and *N*,*N*-dimethylaminopyridine *N*-oxide (DMAPO) cooperatively promote the dehydrative condensation between various carboxylic acids and amines.

## Results and discussion

First, the amide condensation reaction between 2-phenylbutyric acid and benzylamine was examined in the presence of 5 mol% each of boronic acid **2** and a nucleophilic additive under azeotropic reflux conditions in fluorobenzene (bp. 85 °C)3*f* for 17 h (Table 1). Boronic acid **2** did not promote the reaction in the absence of additive under these conditions (entry 1). Tertiary amines such as *N*,*N*-diisopropylethylamine and 4-(*N*,*N*-dimethylamino)pyridine (DMAP)^11^ were not effective as additives (entries 2 and 3). 4-Methoxypyridine *N*-oxide (MPO) was also less active (entry 4). In contrast, a more nucleophilic but weak base, DMAPO,^12^ was quite effective for the amide condensation (entry 5). However, a more nucleophilic additive such as 4-(pyrrolidin-1-yl)pyridine *N*-oxide (PPYO) was less effective than DMAPO (entry 6), perhaps because the strong nucleophilicity of PPYO might reduce the activity of **7**.

**Table 1:** Effect of additives on the dehydrative condensation between 2-phenylbutyric acid and benzylamine^*a*^

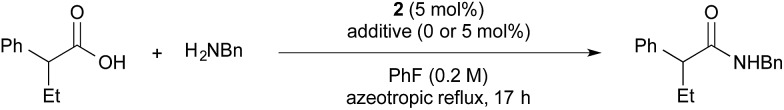					
Entry	Additive	Yield^*b*^ (%)	Entry	Additive	Yield^*b*^ (%)
1	None	<5	4	MPO	<5
2	*i*-Pr_2_EtN	<5	5	DMAPO	99
3	DMAP	<5	6	PPYO	27

^*a*^A solution of 2-phenylbutyric acid (0.5 mmol) and benzylamine (0.5 mmol) in fluorobenzene was heated in the presence of **2** (5 mol%) and additive (0 or 5 mol%) under azeotropic reflux conditions.

^*b*^Isolated yield.

Next, the cooperative effects of several boronic acids (5 mol%) were compared in the condensation reaction between 2-phenylbutyric acid or benzoic acid and benzylamine in the presence of DMAPO (5 mol%) (Table 2). These less reactive carboxylic acids were not activated by the individual use of boronic acids under the same conditions. As expected, **2**–DMAPO and **4b**–DMAPO efficiently activated 2-phenylbutyric acid (entries 1 and 3). Phenylboronic acid and **3** were almost inert, even in the presence of DMAPO (entries 2 and 4). Interestingly, **2**–DMAPO was more effective than **4b**–DMAPO for the amide condensation of benzoic acid (entries 1 and 3). While Whiting's catalyst **3** was quite effective for the amide condensation of benzoic acid, the catalytic activity of **3** was suppressed in the presence of DMAPO (entry 4).^13^

**Table 2:** Cooperative effects of boronic acid–DMAPO on the dehydrative condensation reaction^*a*^

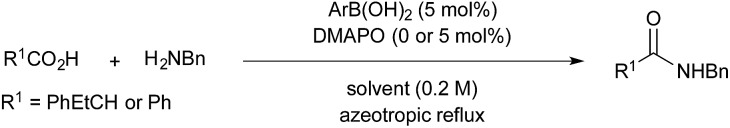			
Entry	ArB(OH)_2_	Yield^*b*^ ^,^^*c*^ (%) of PhEtCHCONHBn	Yield^*b*^ ^,^^*d*^ (%) of PhCONHBn
1	**2**	99 [<5]	97 [<5]
2	PhB(OH)_2_	<5 [<5]	<5 [<5]
3	**4b**	92 [<5]	20 [8]
4	**3**	7 [15]	80 [95]

^*a*^0.5 mmol of carboxylic acid and 0.5 mmol of benzylamine were used in the presence of 5 mol% of ArB(OH)_2_ and 0 or 5 mol% of DMAPO.

^*b*^The results when both catalysts were used are shown. For comparison, the results without DMAPO are shown in brackets.

^*c*^Conditions: fluorobenzene (bp. 85 °C), 17 h.

^*d*^Conditions: toluene (bp. 110 °C), 4 h.

To explore the substrate scope using the cooperative catalysts, **2**–DMAPO, the amide condensation reactions of several less reactive α-branched carboxylic acids and arenecarboxylic acids were examined under azeotropic reflux conditions in fluorobenzene (bp. 85 °C) or toluene (bp. 110 °C). As shown in Table 3, in each example, the cooperative catalysts were much more effective than **2** alone, the results for which are shown in brackets. Notably, not only aliphatic primary amines but also sterically hindered aliphatic secondary amines, less nucleophilic anilines and alkoxyamines reacted with these carboxylic acids. In particular, **2**–DMAPO was effective in the amidation of arenecarboxylic acids with sterically hindered amines, in comparison with **3** and **4b** (entries 9–14). This cooperative method is scalable to practical volumes: the catalytic loading of **2**–DMAPO could be reduced to 2.5 mol% for the dehydrative condensation on an 80 mmol scale (entry 4).

**Table 3:** Cooperative effect of **2**–DMAPO on the dehydrative condensation of α-branched carboxylic acids and arenecarboxylic acids^*a*^

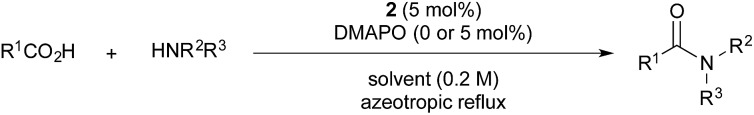			
Entry	Amide	Solvent, time	Yield^*b*^ (%)
1^*c*^	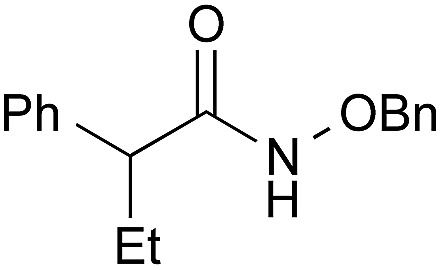	PhF, 25 h	93 [<5]
2^*c*^	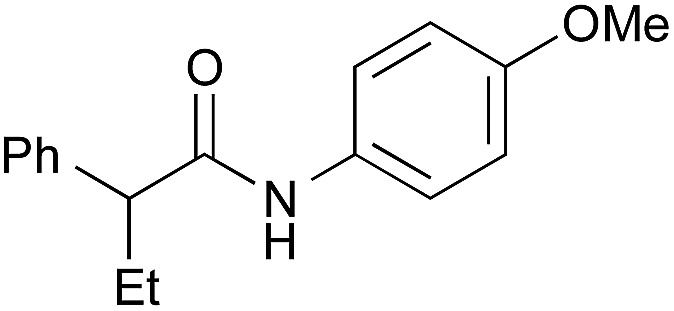	PhF, 17 h	90 [<5]
3	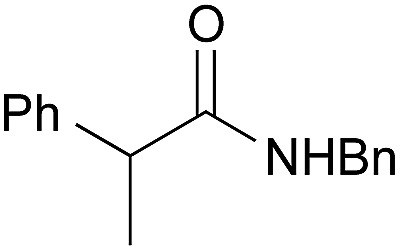	PhH, 8 h	95 [<5]
4^*d*^		PhCH_3_, 11 h	98
5	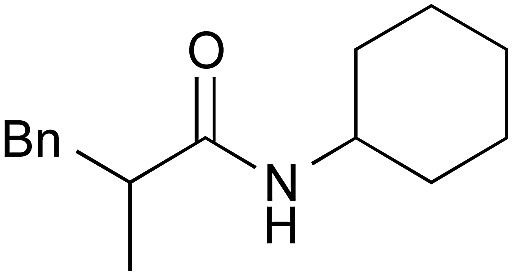	PhCH_3_, 8 h	97 [<5]
6^*c*^	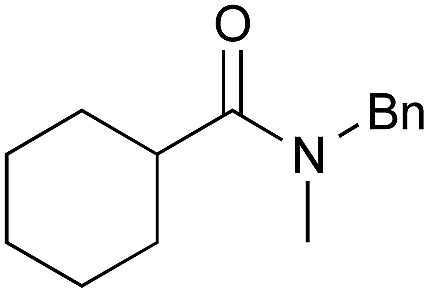	PhCH_3_, 18 h	92 [<5]
7^*c*^	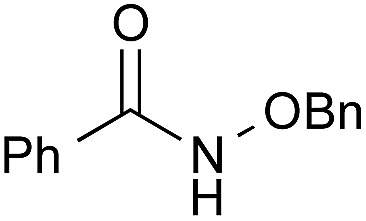	PhCH_3_, 12 h	70 [23]
8	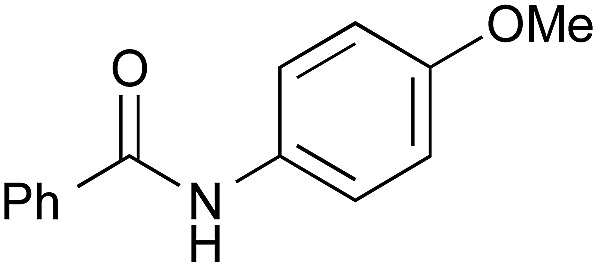	PhCH_3_, 23 h	91 [19]
9^*e*^	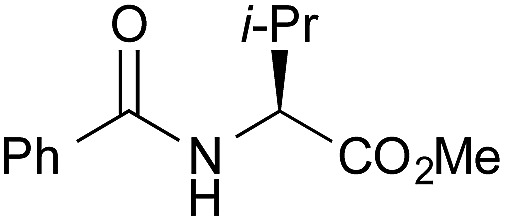	PhF, 23 h	85^*f*^ [30]
10^*e*^ ^,^^*g*^		PhF, 23 h	[2]
11^*e*^ ^,^^*h*^		PhF, 23 h	[17]
12	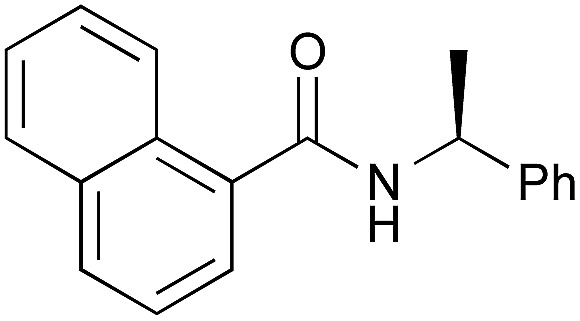	PhCH_3_, 9 h	95 [<5]
13^*g*^		PhCH_3_, 9 h	[39]
14^*h*^		PhCH_3_, 9 h	[<5]
15	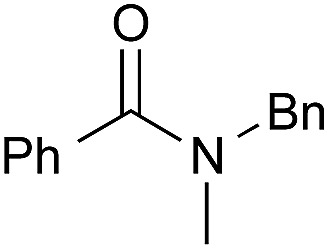	PhCH_3_, 8 h	92 [32]

^*a*^Unless noted otherwise, 0.5 mmol of carboxylic acid and 0.5 mmol of amine were used in the presence of 5 mol% of **2** and 0 or 5 mol% of DMAPO.

^*b*^The results when both catalysts were used are shown. For comparison, the results without DMAPO are shown in brackets.

^*c*^10 mol% of each of the catalysts was used.

^*d*^2.5 mol% of each of **2** and DMAPO was used on an 80 mmol scale in 70 mL of toluene.

^*e*^15 mol% of each of the catalysts was used.

^*f*^99% ee.

^*g*^ **3** was used.

^*h*^ **4b** was used.

The boronic acid-catalysed condensation of relatively more reactive α-nonbranched carboxylic acids with sterically hindered secondary amines and less nucleophilic anilines proceeded even in the absence of DMAPO, as shown in brackets in Table 4.

**Table 4:** Cooperative effects of boronic acid–DMAPO on the dehydrative condensation of α-nonbranched carboxylic acids^*a*^

Entry	Amide	ArB(OH)_2_	Solvent, time	Yield^*b*^ (%)
1^*c*^	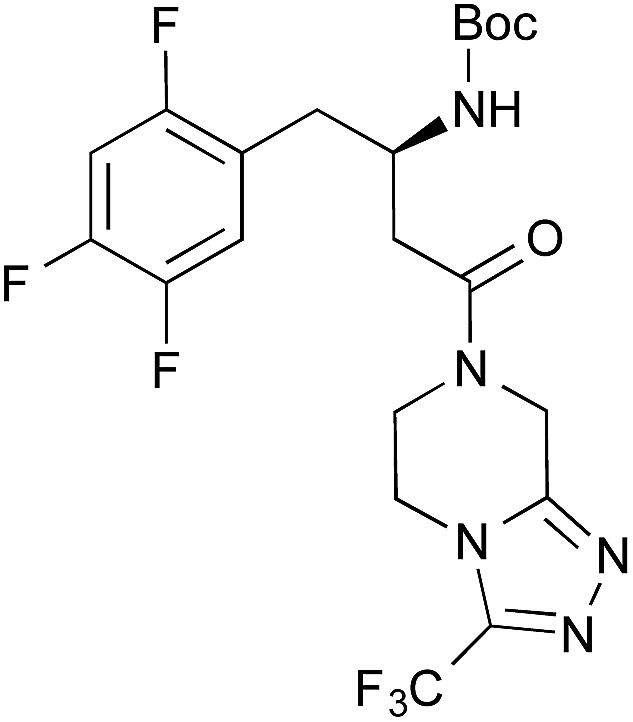	**2**	PhF, 23 h	81 [55]
2^*c*^		PhB(OH)_2_	PhF, 23 h	92 [50]
3^*c*^		**4b**	PhF, 23 h	98 [53]
4^*d*^		**4b**	PhF, 40 h	>99
5^*c*^		—	PhF, 23 h	<5 [<5]
6	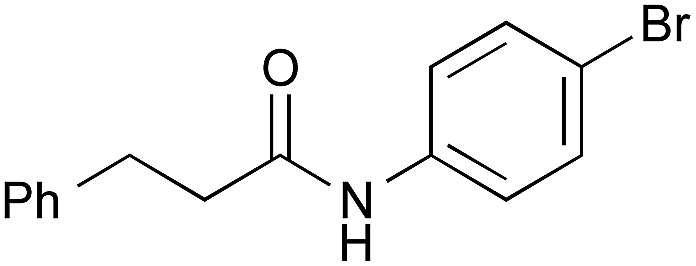	PhB(OH)_2_	PhH, 17 h	82 [44]

^*a*^Unless noted otherwise, 0.55 mmol of carboxylic acid and 0.50 mmol of amine were used in the presence of 5 mol% of ArB(OH)_2_ and 0 or 5 mol% of DMAPO.

^*b*^The results when both catalysts were used are shown. For comparison, the results without DMAPO are shown in brackets.

^*c*^10 mol% of each of the catalysts was used.

^*d*^The reaction was carried out at a 5 mmol scale.

Nevertheless, the addition of DMAPO was also quite effective for these reactions. Interestingly, **4b** and phenylboronic acid were slightly more reactive than **2** in the presence of DMAPO. In particular, the utility of inexpensive phenylboronic acid is industrially significant. This catalytic method is readily scalable. 2.5 g of *N*-Boc protected sitagliptin,^14^ an anti-diabetic drug, was obtained by carrying out the condensation on a 5 mmol scale (entry 4).

The results in Tables 1–4 suggest that both the nucleophilicity of the additive and the Lewis acidity and steric effect of the boronic acid are important in the cooperative catalysis with an ArB(OH)_2_–nucleophilic base (Table 5). The reactivity from highest to lowest followed the order arenecarboxylic acids, α-branched carboxylic acids, α-nonbranched carboxylic acids. As a result, **2** was more effective for arenecarboxylic acids and α-branched carboxylic acids. On the other hand, **4b** and phenylboronic acid were more effective for α-nonbranched carboxylic acids.

**Table 5:** Relationship between the cooperative effects of boronic acid–DMAPO and the reactivity of carboxylic acids

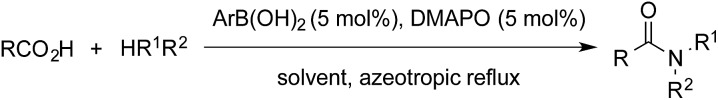			
RCO_2_H	Catalytic activity of ArB(OH)_2_–DMAPO		
	**2**	**4b**	PhB(OH)_2_
ArCO_2_H	High	Low	Low
R^3^R^4^CHCO_2_H	High	Good	Low
R^3^CH_2_CO_2_H	Good	High	High

**Fig. 1:**
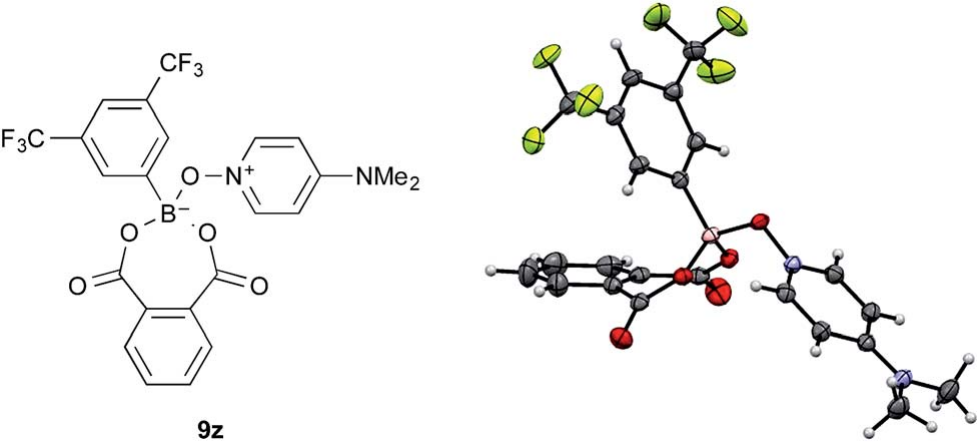
X-ray structure of **9z**.

The amide condensation reaction should occur through the active intermediate **6** (Scheme 2). However, not only **6** but also the undesired complex **8** would be generated in an equilibrium mixture. Complex **8** might be converted to the more stable complex **9**, which is inert to the amide condensation. In fact, the generation of inert complex **9** was ascertained by ^11^B and ^1^H NMR analysis in the amidation of less-hindered carboxylic acids.^15^ Also, the chemical structure of the cyclic complex prepared from **2**, phthalic acid, and DMAPO was determined to be that of **9z** by X-ray diffraction analysis (Fig. 1).^16^

For sterically hindered carboxylic acids such as arenecarboxylic acids and α-branched carboxylic acids, the desired intermediate **7** was preferentially generated. Thus, *o*-nonsubstituted and *m*- or *p*-electron-deficient group-substituted phenylboronic acids such as **1** and **2** were more suitable. In contrast, for less sterically hindered α-nonbranched carboxylic acids, the undesired complex **9** was generated more easily. In addition, the strong Lewis acidity of **2** helped to stabilize **9** by the tight coordination of DMAPO to the boron centre. This is why **4b** and phenylboronic acid were slightly more effective than **2** for the condensation of α-nonbranched carboxylic acids. Not only Lewis acidity, but also the bulkiness of the *o*-substituent of the boronic acid might suppress the generation and stability of **9**. It is noted that the effect of DMAPO was not striking at ambient temperature. Heating was required to accelerate the equilibrium between **6** and **8**.

The utility of the cooperative catalysts was also demonstrated for the selective amide condensation of β-substituted acrylic acids to give the corresponding amides **10** (Table 6). The production of Michael adducts **11** was fairly minimal. In contrast, when boronic acids were used in the absence of DMAPO, the yield and selectivity of the reaction for **10** were moderate. Control experiments ascertained that **10** (*n* = 1) was selectively obtained from **13**,^17^ and **11** (*n* = 1) was not generated from **10** (*n* = 1) but **14**. Amide **10c** is known to be a potential antimitotic agent, especially for brain cancers (entry 6).^18^ The cooperative catalysts were effective for the selective amide condensation of not only β-substituted acrylic acids, but also polyconjugated carboxylic acids and but-2-ynoic acid (entries 12–17).
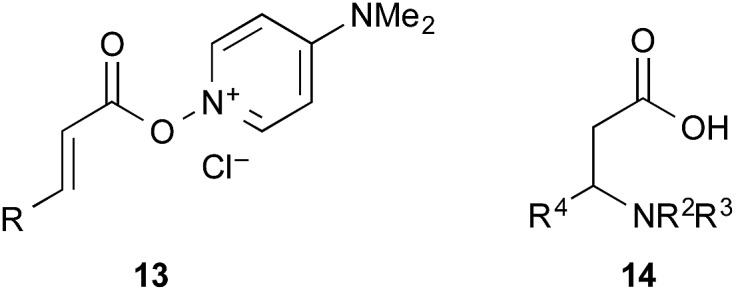


**Table 6:** Cooperative effects of boronic acid–DMAPO on the dehydrative condensation of conjugated carboxylic acids^*a*^

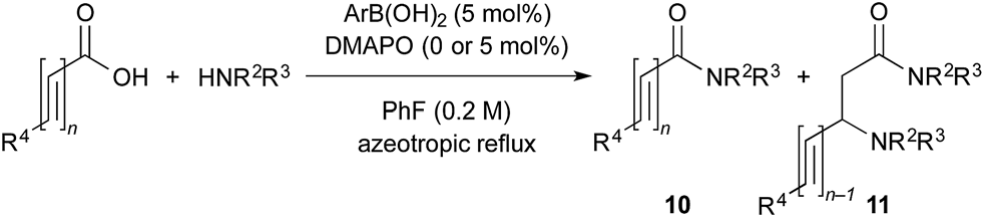				
Entry	ArB(OH)_2_	Time (h)	Product **10** or **12**	
			Yield^*b*^ (%)	Selectivity^*b*^ (%)
1	**2**	12	**10a**, 87 [42]	93 [74]^*c*^
2	**4b**	10	**10a**, 82 [79]	94 [91]^*c*^
3	PhB(OH)_2_	12	**10a**, 15 [4]	95 [78]^*c*^
4	**2**	15	**10b**, 77 [17]	89 [30]^*c*^
5	**4b**	16	**10b**, 65 [29]	89 [47]^*c*^
6^*d*^ ^,^^*e*^	**2**	15	**10c**, 96 [49]	96 [64]^*c*^
7	**2**	38	**10d**, 90 [78]	>95 [>95]^*c*^
8	**2**	8	**10e**, 68 [19]	82 [37]^*c*^
9	**4b**	7	**10e**, 72 [62]	84 [78]^*c*^
10^*d*^	PhB(OH)_2_	19	**10e**, 81 [56]	92 [76]^*c*^
11^*d*^	PhB(OH)_2_	22	**10f**, 73 [32]	96 [55]^*c*^
12^*d*^	**2**	16	**10g**, 98 [45]	>99 [45]^*f*^
13^*d*^	**2**	16	**10h**, 97 [69]	>99 [79]^*f*^
14^*d*^	**2**	16	**10i**, 73 [<5]	97 [<5]^*f*^
15^*d*^	**2**	23	**10j**, 92 [-]	>99 [50]^*f*^
16^*d*^	**2**	24	**10k**, 99 [25]	>99 [56]^*f*^
17^*d*^	**2**	14	**12**, 98 [46]	>99 [82]^*f*^
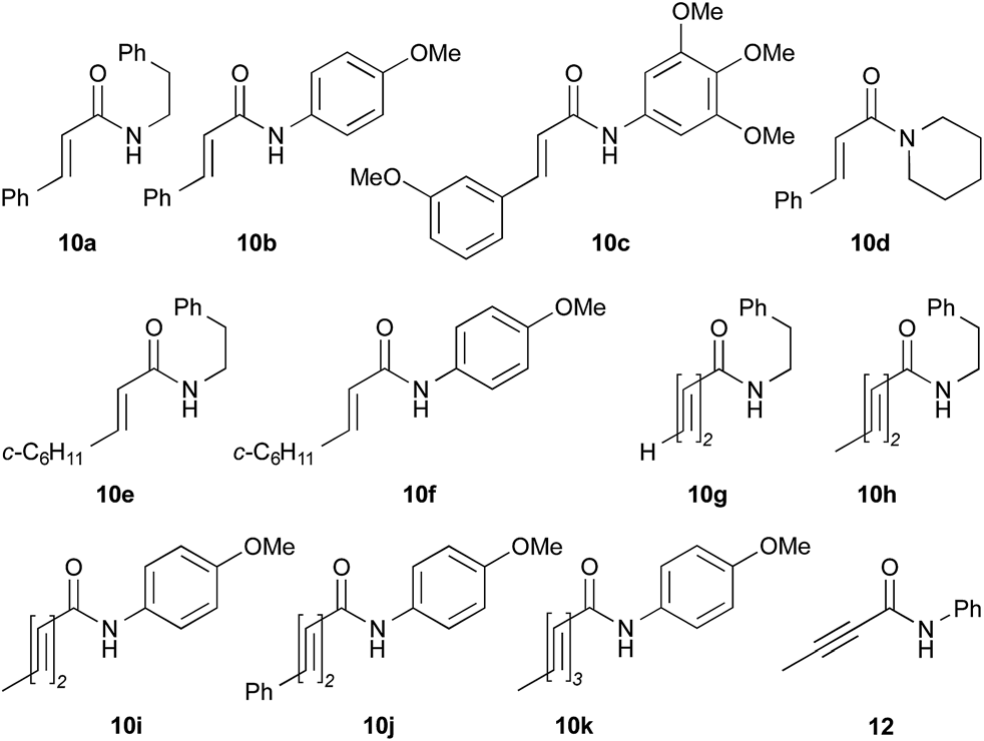				

^*a*^Unless noted otherwise, 0.5 mmol of carboxylic acid and 0.5 mmol of amine were used in the presence of 5 mol% of ArB(OH)_2_ and 0 or 5 mol% of DMAPO.

^*b*^The results when both catalysts were used are shown. For comparison, the results without DMAPO are shown in brackets.

^*c*^β-Aminoamide **11** (*n* = 1) was obtained as the sole minor product.

^*d*^10 mol% of each of the catalysts was used.

^*e*^Toluene was used as a solvent.

^*f*^Several minor products including **11** were obtained.

## Conclusions

In conclusion, this new cooperative catalytic system is quite effective for the amidation reaction of less reactive carboxylic acids, such as sterically hindered α-branched carboxylic acids and arenecarboxylic acids, and the chemoselective amidation reaction of conjugated carboxylic acids. Based on the NMR spectra and X-ray diffraction analysis of inert species **9**, a preliminary mechanism was proposed. Further mechanistic studies are in progress. We believe that these findings will trigger the further development of high-performance amidation catalysts.

## Supplementary Material

Supplementary information

Crystal structure data
