# Supplementary material for: Boronic acid–DMAPO cooperative catalysis for dehydrative condensation between carboxylic acids and amines
Source: Chem Sci. 2015 Nov 6;7(2):1276–80. doi: 10.1039/c5sc03761a (PMC5975839; doi:10.1039/c5sc03761a)
Supplement: Supplementary file 1 [file SC-007-C5SC03761A-s001.pdf]

Electronic Supplementary Material (ESI) for Chemical Science.
This journal is © The Royal Society of Chemistry 2015

Supporting Information
for Boronic Acid–DMAPO Cooperative Catalysis for Dehydrative Condensation between Carboxylic Acids and Amines

Kazuaki Ishihara*†‡ and Yanhui Lu†

†*Graduate School of Engineering, Nagoya University, Furo-cho, Chikusa-ku, Nagoya 464-8603, Japan*

‡*Japan Science and Technology (JST), CREST, Japan*

E-mail: ishihara@cc.nagoya-u.ac.jp

Contents

General Methods……………………………………………………………S2
Detailed Synthetic Procedures and Characterization of the Products…………………S2
Inert Species 9……………………………………………………………S11
Control Experiments for the Chemoselective Dehydrative Condensation of Unsaturated Carboxylic Acids……………………………………………………S15
References……………………………………………………………S17
^{1}H and ^{13}C NMR Charts of New Compounds…………………………………S18

Experimental section

General Methods. IR spectra were recorded on a JASCO FT/IR-460 plus spectrometer. ^{1}H NMR spectra were measured on a JEOL ECS-400 spectrometer (400 MHz) at ambient temperature. Data were recorded as follows: chemical shift in ppm from internal tetramethysilane on the δ scale, multiplicity (s = singlet; d = doublet; t = triplet; m = multiplet), coupling constant (Hz), and integration. ^{13}C NMR spectra were measured on a JEOL ECS-400 (100 MHz). Chemical shifts were recorded in ppm from the solvent resonance employed as the internal standard ($CDCl_3$ at 77.0 ppm). ^{11}B NMR spectra were taken on a JEOL ECS-400 (128 MHz) spectrometer using $B(OMe)_3$ as an external reference. Analytical HPLC was performed on a Shimadzu Model LC-10AD instrument coupled diode array-detector SPD-MA-10A-VP using a column of Daicel CHIRALCEL OD-H (4.6 # 250 mm). Optical rotations were measured on a RUDOLPH AUTOPOL IV digital polarimeter. For TLC analysis, Merck precoated TLC plates (silica gel 60 F254 0.25 mm) were used. For preparative column chromatography, Merck silica gel 60 (0.040–0.063 mm) was used. High resolution mass spectral analysis (HRMS) was performed at Chemical Instrument Facility, Nagoya University.

Dry toluene was purchased from Kanto as the "anhydrous" and stored under nitrogen. Fluorobenzene was purchased from TCI and used directly, benzene was purchased from Wako and used directly. Molecular sieves were activated by heating in a flask by a microwave oven for 1 min and then placed under high vacuum for 10 min.

3,4,5-Trifluorophenylboronic acid **1** (Wako), 3,5-bis(trifluoromethyl)phenylboronic acid **2** (Aldrich), phenylboronic acid (Wako), 4-(*N,N*-dimethylamino)pyridine *N*-oxide (DMAPO) (TCI) and other materials were obtained from commercial supplies and used without further purification. 2-[(*N,N*-diisopropylamino)methyl]phenylboronic acid **3**[1] and 2-iodo-5-methoxyphenylboronic acid **4b**[2] were previously reported.

General procedure for the amide condensation under azeotropic reflux conditions: A 10-mL, single-necked, round-bottomed flask equipped with a Teflon-coated magnetic stirring bar and a 5-mL pressure-equalized addition funnel [containing a cotton plug and ca. 2 g of activated molecular sieves 4Å (pellets)] surmounted by a reflux condenser was charged with carboxylic acid, $ArB(OH)_2$, and DMAPO in fluorobenzene or benzene or toluene (0.2 *M*). Dean-Stark apparatus was also available in place of a pressure-equalized addition funnel containing molecular sieves. After the mixture was stirred at ambient temperature for 5 min, amine (1.0 equiv) was added. The resulting mixture was heated under azeotropic reflux conditions with the removal of water for

several hours. After the reaction mixture was cooled to ambient temperature, the solvent was evaporated. The residue was purified by column chromatography on silica gel (eluents: hexane–EtOAc = 4:1) to give the desired amide products.

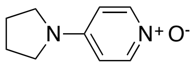
***p*-Pyrrolidinopyridine *N*-Oxide (PPYO)** (Table 1): PPYO was synthesized from *p*-pyrrolidinopyridine (PPY) according to the literature method to prepare DMAPO.[3] 3-Chloroperoxybenzoic acid (*m*-CPBA, 75%, 805 mg, 3.5 mmol) was added to a solution of PPY (435 mg, 2.94 mmol) in dichloromethane (10 mL) at 0 °C. After the reaction mixture was stirred for 5 h at room temperature, it was passed through a column of anion-exchange resin (DOWEX™ 1x2 100–200 Mesh Anion Exchange Resin; Wako Pure Chemical Industries, Ltd.) with methanol, and the filtrate was concentrated by evaporation of the solvent to afford the crude product. The residue was purified by column chromatography on silica gel (NH) (eluents: hexane–EtOAc = 1:1 to chloroform–methanol = 10:1) to give PPYO as a white solid (190 mg, 39% yield). IR (KBr) 1627, 1516, 1461, 1208, 1188, 818 cm^{-1}; ^1H NMR ($CDCl_3$, 400 MHz) δ 2.08 (dt, 4H, *J* = 3.7, 6.4 Hz), 3.33 (t, 4H, *J* = 6.4 Hz), 6.35 (d, 2H, *J* = 6.9 Hz), 7.96 (d, 2H, *J* = 6.9 Hz); ^{13}C NMR ($CDCl_3$, 100 MHz) δ 25.4 (2C), 47.6 (2C), 107.7 (2C), 140.0 (2C), 145.7; HRMS (FAB) calcd for $C_9H_{13}N_2O$ $[M+H]^+$ 165.1028, found 165.1033.

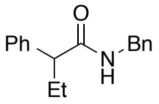
***N*-Benzyl-2-phenylbutanamide** (Tables 1 and 2):[4] white solid; ^1H NMR ($CDCl_3$, 400 MHz) δ 0.89 (t, *J* = 7.3 Hz, 3H), 1.76–1.88 (m, 1H), 2.17–2.29 (m, 1H), 3.25 (t, *J* = 7.8 Hz, 1H), 4.34 (dd, *J* = 5.9, 15.1 Hz, 1H), 4.44 (dd, *J* = 5.9, 14.6 Hz), 5.74 (brs, 1H), 7.14 (d, *J* = 6.4 Hz, 2H), 7.20–7.37 (m, 8H); ^{13}C NMR (100 MHz, $CDCl_3$) δ 12.4, 26.4, 43.4, 55.1, 127.2, 127.3, 127.4 (2C), 128.0 (2C), 128.6 (2C), 128.7 (2C), 138.4, 140.1, 173.6.

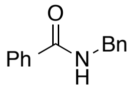
***N*-Benzyl Benzamide** (Table 2):[5] white solid; ^1H NMR ($CDCl_3$, 400 MHz) δ 4.65 (d, *J* = 6.0 Hz, 2H), 6.42 (br, 1H), 7.27–7.54 (m, 8H), 7.79 (d, *J* = 7.3 Hz, 2H); ^{13}C NMR ($CDCl_3$, 100 MHz) δ 44.1, 127.1 (2C), 127.5, 127.9 (2C), 128.6 (2C), 128.8 (2C), 131.5, 134.4, 138.4, 167.5.

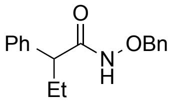
N*-(Benzyloxy)-2-phenylbutanamide** (entry 1, Table 3): white solid; IR (KBr) 3213, 2958, 1655 cm^{-1}; ^1H NMR (DMSO-d_6, 400 MHz) δ 0.80 (t, *J* = 7.3 Hz, 3H), 1.57–1.69 (m,1H), 1.89–2.01 (m, 1H), 3.13 (dd, *J* = 6.4, 8.7 Hz, 1H), 4.73 (s, 2H), 7.20–7.45 (m, 10H), 11.22 (s, 1H); ^{13}C NMR (DMSO-d_6, 100 MHz) δ 12.1, 25.9, 50.0, 76.7, 126.8, 127.7 (2C), 128.2 (5C), 128.9 (2C), 135.9, 140.2, 169.7; HRMS (ESI) calcd for $C_{17}H_{20}NO_2$ $[M+H]^+$ 270.1489, found 270.1490. ***Note:

To a solution of *O*-benzylhydroxylamine hydrochloride (0.50 mmol) in 2 mL of water was added sodium hydrogen carbonate (0.55 mmol, 1.1 equiv) portionwise. Then 3 mL of dichloromethane was added. The resulting mixture was stirred at room temperature for 15 minutes. Organic phases were collected, and water phase was re-extracted with dichloromethane (3 mL x 2). The combined organic layers were washed with brine (5 mL) and dried over anhydrous $MgSO_4$. Removal of the solvent under reduced pressure to give *O*-benzylhydroxylamine in quantitative yield. Then it was used as a substrate amine following general procedure.

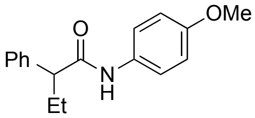

***N*-Benzyl-2-phenylbutanamide** (entry 2, Table 3): white solid; IR (KBr) 3282, 1655, 1604, 1511, 1234 cm^{-1}; ^{1}H NMR ($CDCl_3$, 400 MHz) δ 0.92 (t, *J* = 7.3 Hz, 3H), 1.80–1.91 (m, 1H), 2.21–2.33 (m, 1H), 3.37 (t, *J* = 7.3 Hz, 1H), 3.76 (s, 3H), 6.80 (d, *J* = 6.9 Hz, 2H), 7.04 (br, 1H), 7.25-7.36 (m, 7H); ^{13}C NMR (100 MHz, $CDCl_3$) δ 12.5, 26.6, 55.6, 56.0, 114.1 (2C), 121.8 (2C), 127.5, 128.2 (2C), 129.1 (2C), 131.1, 139.8, 156.5, 171.7; HRMS (ESI) calcd for $C_{17}H_{20}NO_2$ $[M+H]^+$ 270.1489, found 270.1491.

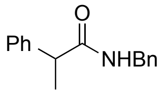

***N*-benzyl-2-phenylpropanamide** (entries 3 and 4, Table 3):[6] White solid; ^{1}H NMR (400 MHz, $CDCl_3$) δ 1.56 (d, *J* = 7.3 Hz, 3H), 3.56 (q, *J* = 7.2 Hz, 1H), 4.37 (dd, *J* = 5.9, 15.1 Hz, 1H), 4.42 (dd, *J* = 5.9, 15.1 Hz, 1H), 5.63 (br, 1H), 7.14 (d, *J* = 6.9 Hz, 2H), 7.21–7.38 (m, 8H); ^{13}C NMR ($CDCl_3$, 100 MHz) δ 18.6, 43.6, 47.1, 127.35, 127.38, 127.5 (2C), 127.7 (2C), 128.7 (2C), 129.0 (2C), 138.4, 141.4, 174.2.

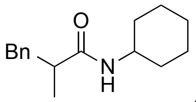

***N*-Cyclohexyl-2-methyl-3-phenylpropanamide** (entry 5, Table 3): White solid; IR (KBr) 3293, 2934, 1638, 1552 cm^{-1}; ^{1}HNMR ($CDCl_3$, 400 MHz) δ 0.74–0.78 (m, 1 H), 0.92–1.14 (m, 2 H), 1.17 (d, *J* = 6.8 Hz, 3 H), 1.21–1.30 (m, 2 H), 1.50–1.72 (m, 4 H), 1.76–1.87 (m, 1 H), 2.28–2.40 (m, 1 H), 2.65 (dd, *J* = 5.9, 13.2 Hz, 1 H), 2.92 (dd, *J* = 8.7, 13.2 Hz, 1 H), 3.60–3.75 (m, 1 H), 5.00 (d, *J* = 7.3 Hz, 1H), 7.12–7.32 (m, 5H); ^{13}C NMR ($CDCl_3$, 100 MHz) δ 17.9, 24.8, 24.9, 25.6, 33.0, 33.1, 40.7, 44.1, 47.8, 126.3, 128.4 (2C), 129.0 (2C), 140.1, 174.5. HRMS (FAB) calcd for $C_{16}H_{23}NO$ $[M+H]^+$ 246.1858, found 246.1857.

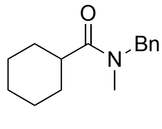

***N*-Benzyl-*N*-methylcyclohexanecarboxamide** (entry 6, Table 3):[7] colorless oil; a mixture of two rotamers; ^{1}H NMR ($CDCl_3$, 400 MHz) δ 1.12–1.39 (m, 3H), 1.51–1.89 (m, 7H), 2.46–2.62 (m, 1H), 2.91 (s, 1.2H), 2.94 (s, 1.8H), 4.56 (s, 0.8H), 4.59 (s, 1.2H), 7.12–7.44 (m, 5H); ^{13}C NMR ($CDCl_3$, 100 MHz) δ 25.6 (0.8C), 25.7 (1.2C), 29.1 (1.2C), 29.5 (0.8C), 33.7 (0.4C), 34.4

(0.6C), 40.5 (0.4C), 40.6 (0.6C), 50.5, 52.9, 126.1, 127.0 (0.6C), 127.4 (0.4C), 127.7, 128.4, 128.7, 136.9 (0.4C), 137.6 (0.6C), 176.0 (0.6C), 176.6 (0.4C).

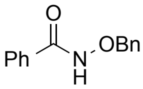

N*-(Benzyloxy)benzamide** (entry 7, Table 3):[8] white solid; ^{1}H NMR (DMSO-d_6, 400 MHz) δ 4.93 (s, 2H), 7.32–7.57 (m, 8H), 7.74 (d, *J* = 6.9 Hz, 2H), 11.77 (brs, 1H); ^{13}C NMR (DMSO-d_6, 100 MHz) δ 77.0, 127.1 (2C), 128.3 (3C), 128.5 (2C), 128.9 (2C), 131.6, 132.4, 135.9, 164.4. ***Note: To a solution of *O*-benzylhydroxylamine hydrochloride (0.5 mmol) in 2 mL of water was added sodium hydrogen carbonate (0.55 mmol, 1.1 equiv) portionwise. Then 3 mL of dichloromethane was added. The resulting mixture was stirred at room temperature for 15 minutes. Organic phases were collected, and water phase was re-extracted with dichloromethane (3 mL x 2). The combined organic layers were washed with brine (5 mL) and dried over anhydrous $MgSO_4$. Removal of the solvent under reduced pressure to give *O*-benzylhydroxylamine in quantitative yield. Then it was used as a substrate amine following general procedure.

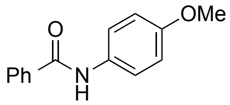

***N*-(4-Methoxyphenyl)benzamide** (entry 8, Table 3):[9] white solid; ^{1}H NMR ($CDCl_3$, 400 MHz) δ 3.82 (s, 3H), 6.91 (d, *J* = 9.1 Hz, 2H), 7.45–7.57 (m, 5H), 7.73 (br, 1H), 7.86 (d, *J* = 7.4 Hz, 2H); ^{13}CNMR ($CDCl_3$, 100 MHz) δ 55.6, 114.3 (2C), 122.4 (2C), 127.1 (2C), 128.8 (2C), 131.1, 131.8, 135.1, 156.8, 165.9.

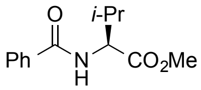

Methyl Benzoyl-L-valinate (entries 9–11, Table 3):[10] white solid; HPLC (Daicel Chiral OD-H, hexane/Ethanol = 90:10, flow rate = 1.0 mL/min), t_R = 5.79 (minor enantiomer), t_R = 6.64 (major enantiomer) min, 99% ee; ^{1}H NMR ($CDCl_3$, 400 MHz) 0.95–1.04 (m, 6H), 2.22–2.38 (m, 1H), 3.78 (s, 3H), 4.80 (dd, *J* = 4.6, 8.7 Hz, 1H), 6.64 (brd, *J* = 8.4 Hz, 1H), 7.45 (tm, *J* = 7.3 Hz, 2H), 7.52 (tm, *J* = 7.3 Hz, 1H), δ 7.81 (dm, *J* = 7.3 Hz, 2H); ^{13}C NMR ($CDCl_3$, 100 MHz) δ 18.0, 19.1, 31.6, 52.3, 57.5, 127.1 (2C), 128.6 (2C), 131.8, 134.1, 167.4, 172.7. ***Note***: To a solution of L-valine methyl ester hydrochloride (0.6 mmol) in 2 mL of water was added sodium hydrogen carbonate (0.63 mmol, 1.05 equiv) portionwise. Then 3 mL of dichloromethane was added. The resulting mixture was stirred at room temperature for 15 minutes. Organic phase was collected, and water phase was re-extracted with dichloromethane (3 mL x 2). The combined organic layers were washed with brine (5 mL) and dried over anhydrous $MgSO_4$. Removal of the solvent under reduced pressure to give L-valine methyl ester in quantitative yield. Then it was used as a substrate amine following general procedure.

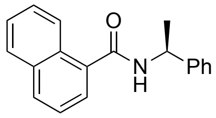

(*S*)-*N*-(1-Phenylethyl)-1-naphthamide (entry 12–14, Table 3): white solid; IR (KBr) 3316, 1633, 1523 cm^{-1}; ^{1}H NMR ($CDCl_3$, 400 MHz) δ 1.67 (d, *J* = 6.9 Hz, 3H), 5.47 (quint, *J* = 7.3 Hz, 1H), 6.21 (brd, *J* = 5.5 Hz, 1H), 7.28–7.56 (m, 8H), 7.60 (d, *J* = 6.9 Hz, 1H), 7.86 (d, *J* = 6.9 Hz, 1H), 7.91 (d, *J* = 8.2 Hz, 1H), 8.28 (d, *J* = 8.2 Hz, 1H); ^{13}C NMR (100 MHz, $CDCl_3$) δ 22.0, 49.4, 124.7, 124.9, 125.5, 126.3 (2C), 126.5, 127.1, 127.5, 128.4, 128.8 (2C), 130.2, 130.6, 133.7, 134.6, 143.2, 168.7; HRMS (ESI) calcd for $C_{19}H_{18}NO$ $[M+H]^+$ 276.1383, found 276.1383.

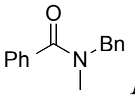

***N*-Benzyl-*N*-methylbenzamide** (entry 15, Table 3):[5] colorless oil; a mixture of two rotamers; ^{1}H NMR ($CDCl_3$, 400 MHz) δ 2.86 (s, 1.5H), 3.03 (s, 1.5H), 4.51 (s, 1H), 4.76 (s, 1H), 7.14–7.50 (m, 10H); ^{13}C NMR ($CDCl_3$, 100 MHz) δ 33.1 (0.5C), 36.9 (0.5C), 50.7 (0.5C), 55.1 (0.5C), 126.7, 126.9, 127.4 (0.5C), 127.5 (0.5C), 128.1, 128.3 (2C), 128.6, 128.8, 129.5 (2C), 136.2, 136.5 (0.5C), 137.0 (0.5C), 171.5 (0.5C), 172.2 (0.5C).

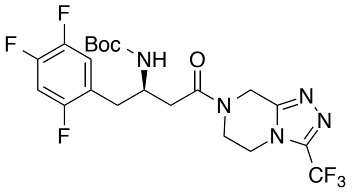

tert*-Butyl (*R*)-(4-Oxo-4-(3-(trifluoromethyl)-5,6-dihydro[1,2,4]-triazolo[4,3-*a*]pyrazin-7(8*H*)-yl)-1-(2,4,5-trifluorophenyl)butan-2-yl)carbamate** (entries 1–5, Table 4):[11] white solid; IR (KBr) 3365, 2979, 2935, 1679, 1655, 1523, 1423, 1167, 1132, 1017, 851 cm^{-1}; ^{1}H NMR (400 MHz, $CDCl_3$) δ 1.36 (s, 9H), 2.63–2.97 (m, 4H), 3.97–4.30 (m, 5H), 4.92 (brs, 1H), 5.01–5.12 (m, 1H), 5.22–5.32 (m, 1H), 6.83–6.92 (m, 1H), 7.04–7.11 (m, 1H); ***Note: 3-(Trifluoromethyl)-5,6,7,8-tetrahydro-[1,2,4]triazolo[4,3-*a*]pyrazine hydrogen chloride (0.5 mmol, 114.3 mg) was dissolved in 125 μL of 4 *M* NaOH solution and stirred at room temperature for 10 minutes. Then (*R*)-3-[(*tert*-butoxycarbonyl)amino]-4-(2,4,5-trifluorophenyl)butanoic acid (0.55 mmol, 183.3 mg) was added. After removing water under vacuum, boronic acids (or and DMAPO) and fluorobenzene (2.5 mL) were added successively. Then following the general procedure to azeotropic reflux. Then the mixture was diluted by ethyl acetate (20 mL), and washed by K_2CO_3 (0.5 M, 10 mL) and brine, dried over anhydrous Na_2SO_4. Removing solvents to give the crude product. Then, the crude residue was purified by trituration over hexane (50 mL) for 5 min at room temperature. Then, the solid was filtrated over a Buchner funnel, then the collected precipitate was dried under vacuum to give the pure product as a white solid.

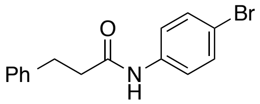

***N*-(4-Bromophenyl)-3-phenylpropanamide** (entry 6, Table 4): white solid; IR (KBr) 3300, 1655, 1523 cm^{-1}; ^{1}H NMR ($CDCl_3$, 400 MHz) δ 2.66 (t, *J* = 7.8 Hz, 2H), 3.05 (t, *J* =

7.8 Hz, 2H), 6.96 (brs, 1H), 7.23–7.33 (m, 7H), 7.40 (d, *J* = 8.7 Hz, 2H); ^{13}C NMR ($CDCl_3$, 100 MHz) δ 31.6, 39.5, 117.0, 121.6 (2C), 126.6, 128.5 (2C), 128.8 (2C), 132.0 (2C), 136.9, 140.5, 170.6; HRMS (ESI) calcd for $C_{15}H_{14}BrNNaO$ $[M+Na]^+$ 326.0151, found 326.0153.

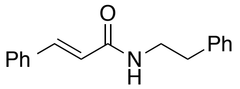

***N*-(2-Phenylethyl)cinnamide (10a)**[12] (entries 1–3, Table 6): white solid; ^{1}H NMR (400 MHz, $CDCl_3$) δ 2.88 (t, *J* = 6.9 Hz, 2H), 3.64 (dt, *J* = 6.9, 13.3 Hz, 2H), 5.98 (br s, 1H), 6.36 (d, *J* = 15.6 Hz, 1H), 7.16–7.36 (m, 8H), 7.41–7.49 (m, 2H), 7.61 (d, *J* = 15.6 Hz, 1H); ^{13}C NMR (100 MHz, $CDCl_3$) δ 35.7, 41.0, 122.0, 126.5, 127.8 (2C), 128.6 (2C), 128.76 (2C), 128.78 (2C), 129.6, 134.8, 138.9, 140.8, 166.2.

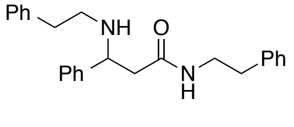

***N*-(2-Phenethyl)-3-((2-phenethylamino)-3-phenylpropanamide (11a)** (entries 1–3, Table 6): colorless oil; IR (neat) 3283, 3027, 2927, 1647, 1542, 1454, 749, 699 cm^{-1}; ^{1}H NMR ($CDCl_3$, 400 MHz) δ 2.39 (dd, *J* = 4.2, 15.6 Hz, 1H), 2.47 (dd, *J* = 10.1, 16.0 Hz, 1H), 2.56–2.76 (m, 7H), 3.36–3.44 (m, 2H), 3.87 (dd, *J* = 4.2, 9.6 Hz, 1H), 7.08–7.37 (m, 15H), 7.49 (brs, 1H); ^{13}C NMR ($CDCl_3$, 100 MHz) δ 35.7, 36.2, 40.2, 44.0, 48.1, 60.0, 126.4, 126.5, 126.6 (2C), 127.6, 128.6 (2C), 128.7 (2C), 128.75 (2C), 128.8 (2C), 128.9 (2C), 139.3, 139.8, 142.4, 171.4; HRMS (ESI) calcd for $C_{25}H_{29}N_2O$ $[M+H]^+$ 373.2274, found 373.2274.

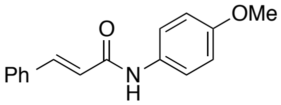

***N*-(4-Methoxyphenyl)cinnamamide (10b)**[13] (entries 4 and 5, Table 6): ^{1}H NMR ($CDCl_3$, 400 MHz) δ 3.80 (s, 3H), 6.54 (d, *J* = 15.6 Hz, 1H), 6.89 (d, *J* = 9.2 Hz, 2H), 7.30–7.38 (m, 4H), 7.50–7.55 (m, 4H), 7.74 (d, *J* = 15.6 Hz, 1H); ^{13}C NMR ($CDCl_3$, 100 MHz) δ 55.6, 114.3 (2C), 121.0, 122.1 (2C), 128.0 (2C), 129.0 (2C), 130.0, 131.2, 134.7, 142.1, 156.6, 164.3.

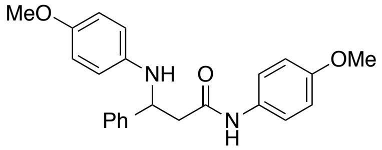

***N*-(4-Methoxyphenyl)-3-((4-methoxyphenyl)amino)-3-phenylpropanamide (11b)** (entries 4 and 5, Table 6): White solid; IR (KBr) 3401, 3360, 1666, 1514, 1238 cm^{-1}; ^{1}H NMR ($CDCl_3$, 400 MHz) δ 2.78 (d, *J* = 6.0 Hz, 2H), 3.70 (s, 3H), 3.76 (s, 3H), 4.77 (t, *J* = 6.0 Hz, 1H), 6.58 (d, *J* = 9.2 Hz, 2H), 6.70 (d, *J* = 8.7 Hz, 2H), 6.80 (d, *J* = 9.2 Hz, 2H), 7.23–7.34 (m, 7H), 7.94 (brs, 1H); ^{13}C NMR ($CDCl_3$, 100 MHz) δ 45.3, 55.5, 55.7, 56.9, 114.2 (2C), 114.8 (2C), 116.1 (2C), 122.3 (2C), 126.3 (2C), 127.6, 128.9 (2C), 130.7, 140.6, 142.5, 152.8, 156.6, 169.1; HRMS (ESI) calcd for $C_{23}H_{25}N_2O_3$ $[M+H]^+$ 377.1860, found 377.1863.

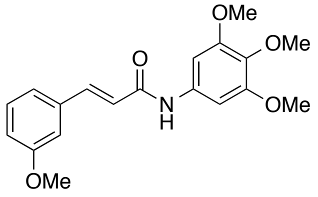

(*E*)-3-(3-Methoxyphenyl)-*N*-(3,4,5-trimethoxyphenyl)acrylamide (10c)[14] (entry 6, Table 6): ^{1}H NMR ($CDCl_3$, 400 MHz) δ 3.81 (s, 3H), 3.82 (s, 6H), 3.83 (s, 3H), 6.57 (d, *J* = 15.6 Hz, 1H), 6.92 (dd, *J* = 2.3, 8.3 Hz, 1H), 6.98 (s, 2H), 7.02 (s, 1H), 7.09 (d, *J* = 7.4 Hz, 1H), 7.28 (t, *J* = 8.2 Hz, 1H), 7.71 (d, *J* = 15.1 Hz, 1H), 7.79 (brs, 1H); ^{13}C NMR ($CDCl_3$, 100 MHz) δ 55.3, 55.9 (2C), 61.1, 97.7 (2C), 113.4, 115.6, 120.4, 121.3, 130.0, 134.3, 134.8, 135.9, 142.2, 153.3 (2C), 159.9, 164.6.

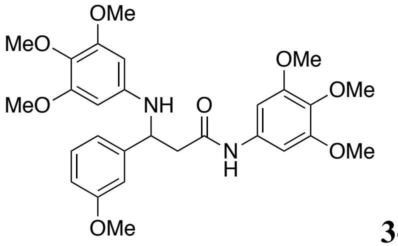

3-(3-Methoxyphenyl)-*N*-(3,4,5-trimethoxyphenyl)-3-((3,4,5-trimethoxyphenyl)amino)propanamide (11c) (entry 6, Table 6)**:** white solid; IR (KBr) 3348, 2937, 1671, 1609, 1508, 1451, 1411, 233, 1127, 1008 cm^{-1}; ^{1}H NMR ($CDCl_3$, 400 MHz) δ 2.81–2.82 (m, 2H), 3.70 (s, 6H), 3.72 (s, 3H), 3.75 (s, 3H), 3.80 (s, 3H), 3.82 (s, 6H), 4.78–4.79 (m, 2H), 5.85 (s, 2H), 6.60 (s, 2H), 6.83 (dd, *J* = 2.3, 8.2 Hz, 1H), 6.95 (brs, 1H), 6.99 (brd, *J* = 7.3 Hz, 1H) 7.22–7.31 (m, 2H); ^{13}C NMR ($CDCl_3$, 100 MHz) δ 45.5, 55.3, 55.8 (2C), 56.05 (2C), 56.1, 61.0, 61.1, 91.5 (2C), 97.9 (2C), 112.0, 112.7, 118.5, 129.9, 130.1, 133.8, 134.7, 143.8, 144.5, 153.3 (2C), 153.7 (2C), 160.2, 169.1; HRMS (ESI) calcd for $C_{28}H_{34}N_2NaO_8$ $[M+Na]^+$ 549.2207, found 549.2207.

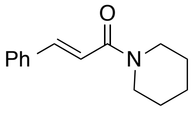

***(E)*-3-Phenyl-1-(piperidin-1-yl)prop-2-en-1-one (10d)**[15] (entry 7, Table 6): white solid; ^{1}H NMR ($CDCl_3$, 400 MHz) δ 1.61–1.69 (m, 6H), 3.59 (brs, 2H), 3.67 (brs, 2H), 6.91 (d, *J* = 15.1 Hz, 1H), 7.33–7.39 (m, 3H), 7.50–7.54 (m, 2H), 7.65 (d, *J* = 15.1 Hz, 1H); ^{13}C NMR ($CDCl_3$, 100 MHz) δ 24.8, 25.7, 26.9, 43.4, 47.1, 117.8, 127.8 (2C), 128.9 (2C), 129.5, 135.6, 142.2, 165.4.

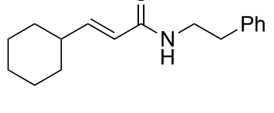

(*E*)-3-Cyclohexyl-*N*-(2-phenethyl)acrylamide (10e) (entries 8–10, Table 6): white solid; IR (KBr) 3298, 2915, 2849, 1672, 1630, 1561 cm^{-1}; ^{1}H NMR ($CDCl_3$, 400 MHz) δ 1.07–1.36 (m, 5H), 1.64–1.80 (m, 5H), 2.05–2.14 (m, 1H), 2.85 (t, *J* = 6.9 Hz, 1H), 3.59 (dt, *J* = 6.9, 13.3 Hz, 1H), 5.48 (brs, 1H), 5.64 (d, *J* = 15.6 Hz, 1H), 6.78 (dd, *J* = 6.9, 15.6 Hz, 1H), 7.19–7.37 (m, 5H); ^{13}C NMR ($CDCl_3$, 100 MHz) δ 25.8 (2C), 26.0, 32.0 (2C), 35.8, 40.3, 40.75, 121.1, 126.5,

128.7 (2C), 128.8 (2C), 139.0, 150.0, 166.6. HRMS (ESI) calcd for $C_{17}H_{23}NNaO$ $[M+Na]^+$ 280.1672, found 280.1673.

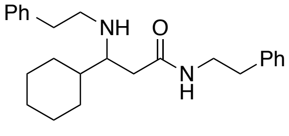

3-Cyclohexyl-*N*-(2-phenethyl)-3-(2-phenethylamino)propanamide (11e) (entries 8–10, Table 6): colorless oil; IR (neat) 3296, 2925, 2851, 1646, 1543, 1452, 748, 699 cm^{-1}; ^1H NMR ($CDCl_3$, 400 MHz) δ 0.72–0.94 (m, 2H), 0.98–1.22 (m, 3H), 1.28–1.74 (m, 7H), 2.05 (dd, J = 8.2, 16.0 Hz, 1H), 2.30 (dd, J = 3.2, 16.0 Hz, 1H), 2.46–2.51 (m, 1H), 2.54–2.76 (m, 5H), 2.78–2.87 (m, 1H), 3.33–3.46 (m, 2H), 7.13–7.32 (m, 10H), 8.30 (brs, 1H); ^{13}C NMR ($CDCl_3$, 100 MHz) δ 26.3, 26.4, 26.5, 27.9, 30.2, 35.7, 36.1, 36.5, 39.5, 39.9, 47.5, 60.1, 126.4 (2C), 128.5 (2C), 128.6 (2C), 128.7 (2C), 128.9 (2C), 139.5, 139.8, 172.7; HRMS (ESI) calcd for $C_{25}H_{35}N_2O$ $[M+H]^+$ 379.2744, found 379.2746.

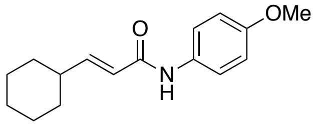

(*E*)-3-Cyclohexyl-*N*-(4-methoxyphenyl)acrylamide (10f) (entry 11, Table 6): white solid; IR (KBr) 3301, 2920, 1667, 1637, 1534, 1511, 1247 cm^{-1}; ^1H NMR ($CDCl_3$, 400 MHz) δ 1.11–1.33 (m, 5H), 1.66–1.78 (m, 5H), 2.01–2.22 (m, 1H), 3.80 (s, 3H), 5.83 (d, J = 15.6 Hz, 1H), 6.85–6.95 (m, 3H), 7.01 (brs, 1H), 7.47 (d, J = 8.3 Hz, 2H); ^{13}C NMR ($CDCl_3$, 100 MHz) δ 25.9 (2C), 26.1, 32.0 (2C), 40.4, 55.6, 114.2 (2C), 121.6, 121.9 (2C), 131.4, 151.1, 156.4, 164.6; HRMS (ESI) calcd for $C_{16}H_{22}NO_2$ $[M+H]^+$ 260.1645, found 260.1649.

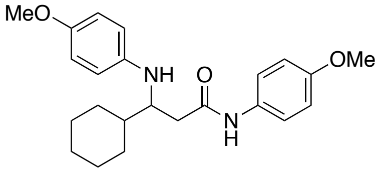

3-Cyclohexyl-*N*-(4-methoxyphenyl)-3-((4-methoxyphenyl)-amino)propanamide (11f) (entry 11, Table 6): White solid; IR (KBr) 3318, 2925, 1639, 1510, 1238 cm^{-1}; ^1H NMR ($CDCl_3$, 400 MHz) δ 0.99–1.26 (m, 5H), 1.60–1.77 (m, 6H), 2.41 (dd, J = 9.2, 15.1 Hz, 1H), 2.55 (dd, J = 2.3, 15.1 Hz, 1H), 3.50-3.60 (m, 2H), 3.77 (s, 6H), 6.72 (d, J = 9.2 Hz, 2H), 6.79–6.82 (m, 4H), 7.26–7.30 (m, 2H), 8.81 (brs, 1H); ^{13}C NMR ($CDCl_3$, 100 MHz) δ 26.3, 26.33, 26.5, 28.3, 29.8, 38.8, 41.6, 55.5, 55.8, 58.0, 114.1 (2C), 115.1 (2C), 116.5 (2C), 121.7(2C), 131.3, 141.0, 153.1, 156.2, 170.4; HRMS (ESI) calcd for $C_{23}H_{31}N_2O_3$ $[M+H]^+$ 383.2329, found 383.2329.

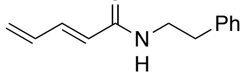

(*E*)-*N*-(2-Phenethyl)penta-2,4-dienamide (10g) (entry 11, Table 6): white solid; IR (KBr) 3249, 1656, 1619, 1553, 1334, 1276, 1012, 922, 868, 701 cm^{-1}; ^1H NMR ($CDCl_3$, 400 MHz) δ 2.85 (t, J = 6.8 Hz, 2H), 3.61 (dt, J = 6.9, 13.8 Hz, 2H), 5.42 (d, J = 10.6 Hz, 1H), 5.47

(brs, 1H), 5.55 (d, J = 17.0 Hz, 1H), 5.79 (d, J = 14.6 Hz, 1H), 6.35–6.44 (m, 1H), 7.16–7.34 (m, 6H); ^{13}C NMR ($CDCl_3$, 100 MHz) δ 35.7, 40.9, 124.5, 124.7, 126.6, 128.7 (2C), 128.9 (2C), 134.8, 138.9, 141.2, 166.0; HRMS (ESI) calcd for $C_{13}H_{15}NNaO$ $[M+Na]^+$ 224.1046, found 224.1044.

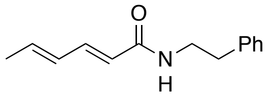

(2*E*,4*E*)-*N*-(2-Phenethyl)hexa-2,4-dienamide (10h) (entry 13, Table 6): white solid; IR (KBr) 3260, 1655, 1605, 1559, 1351, 1264, 1007, 698 cm^{-1}; ^{1}H NMR ($CDCl_3$, 400 MHz) δ 1.82 (d, J = 5.5 Hz, 3H), 2.85 (t, J = 6.9 Hz, 2H), 3.60 (dt, J = 6.0, 12.8 Hz, 2H), 5.41 (brs, 1H), 5.65 (d, J = 14.7 Hz, 1H), 6.03–6.16 (m, 2H), 7.15–7.33 (m, 6H); ^{13}C NMR ($CDCl_3$, 100 MHz) δ 18.7, 35.8, 40.8, 121.5, 126.6, 128.7 (2C), 128.9 (2C), 129.7, 137.9, 139.0, 141.3, 166.5. HRMS (ESI) calcd for $C_{14}H_{17}NNaO$ $[M+Na]^+$ 238.1202, found 238.1197.

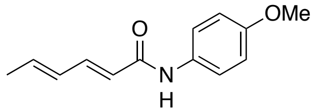

(2*E*,4*E*)-*N*-(4-Methoxyphenyl)hexa-2,4-dienamide (10i) (entry 14, Table 6): white solid; IR (KBr) 3305, 1655, 1609, 1509, 1343, 1293, 1236, 1142, 1032, 826 cm^{-1}; ^{1}H NMR ($CDCl_3$, 400 MHz) δ 1.86 (d, J = 5.5 Hz, 3H), 3.80 (s, 3H), 5.86 (d, J = 14.7 Hz, 1H), 6.10–6.25 (m, 2H), 6.87 (d, J = 9.2 Hz, 2H), 7.03 (brs, 1H), 7.31 (dd, J = 10.1, 14.6 Hz, 1H), 7.48 (d, J = 8.3 Hz, 2H); ^{13}C NMR (DMSO-d_6, 100 MHz) δ 18.4, 55.2, 113.9 (2C), 120.6 (2C), 123.1, 130.0, 132.6, 137.5, 140.3, 155.2, 163.5; HRMS (ESI) calcd for $C_{13}H_{15}NNaO_2$ $[M+Na]^+$ 240.0995, found 240.0991.

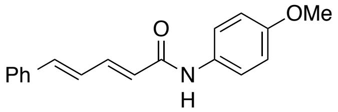

(2*E*,4*E*)-*N*-(4-Methoxyphenyl)hexa-2,4-dienamide (10j) (entry 15, Table 6) white solid; IR (KBr) 3301, 1654, 1542, 1508, 1237 cm^{-1}; ^{1}H NMR ($CDCl_3$, 400 MHz) δ 3.81 (s, 3H), 6.09 (d, J = 14.6 Hz, 1H), 6.87–6.92 (m, 4H), 7.10 (brs, 1H), 7.30-7.38 (m, 3H), 7.46–7.55 (m, 5H); ^{13}C NMR (DMSO-d_6, 100 MHz) δ 55.2, 113.9 (2C), 120.7 (2C), 125.6, 126.9, 127.1 (2C), 128.7, 128.8 (2C), 132.6, 136.3, 138.6, 140.3, 155.2, 163.3; HRMS (ESI) calcd for $C_{18}H_{17}NNaO_2$ $[M+Na]^+$ 302.1151, found 302.1150.

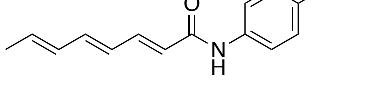

(2*E*,4*E*)-*N*-(4-Methoxyphenyl)hexa-2,4-dienamide (10k) (entry 16, Table 6): white solid; IR (KBr) 3284, 1650, 1604, 1533, 1509, 1238, 1139, 1011, 826 cm^{-1}; ^{1}H NMR ($CDCl_3$, 400 MHz) δ 1.82 (d, J = 6.9 Hz, 3H), 3.78 (s, 3H), 5.87–5.96 (m, 2H), 6.11–6.23 (m, 2H), 6.52 (dd, J = 11.0, 15.1 Hz, 1H), 6.85 (d, J = 9.2 Hz, 2H), 7.12 (brs, 1H), 7.34 (dd, J = 11.0, 14.6 Hz, 1H), 7.47 (d, J = 8.2 Hz, 2H); ^{13}C NMR (DMSO-d_6, 100 MHz) δ 18.4, 55.2, 113.9 (2C), 120.6 (2C), 124.4, 128.1, 131.5, 132.6, 133.8, 139.5, 140.3, 155.2, 163.4; HRMS (ESI) calcd for $C_{15}H_{17}NNaO_2$ $[M+Na]^+$ 266.1151, found 266.1149.

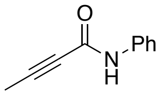

N-Phenylbut-2-ynamide (12) (entry 17, Table 6): white solid; IR (KBr) 3258, 2237, 1637, 1597, 1558, 1440, 1329, 1263, 752 cm^{-1}; ^{1}H NMR ($CDCl_3$, 400 MHz) δ 2.00, (s, 3H), 7.13 (t, J = 7.3 Hz, 1H), 7.33 (t, J = 7.3 Hz, 2H), 7.43 (brs, 1H), 7.50 (d, J = 7.8 Hz, 2H); ^{13}C NMR ($CDCl_3$, 100 MHz) δ 3.8, 75.4, 84.7, 120.0 (2C), 124.8, 129.1 (2C), 137.5, 151.4; HRMS (ESI) calcd for $C_{10}H_{10}NO$ $[M+H]^+$ 160.0757, found 160.0756.

The inert species 9

The inert species **9** generated from direct condensation reaction catalyzed by **2** and DMAPO (entries 1 and 3, Table 4) was isolated and it was determined to be three components of **2**, DMAPO, and carboxylic acid were contained in a 1:1:2 molar ratio in **9** by ^{1}H- and ^{13}B-NMR analyses. The stability of **9** (entries 1 and 3, Table 4) is shown in wet deuterated chloroform and benzene (Table S1). The hydrolysis of inert species **9** (generated from **2** or **4b**) was slow in wet solvents at room temperature. Inert species **9** was more stable in less polar solvents and should be even more stable under dehydrative conditions. There is no doubt that the generation of stable inert species during azeotropic reflux would suppress the catalytic condensation between carboxylic acid and amines. In contrast, inert species **9** generated from **4b** was less stable than inert species **9** generated from **2**. Thus, it cannot be excluded that inert species **9** generated from **4b** might break down and regenerated active species *in situ*. And also the sterically bulky *o*-substituent of **4b** might prevent the coordination between boron center and DMAPO, which decreased the risk to generate inert species. They are why **4b**·DMAPO gave a higher chemical yield than **2**·DMAPO for α-nonbranched carboxylic acids.

Table S1. ^{1}H NMR Experiments: Stability of **9** in Wet $CDCl_3$

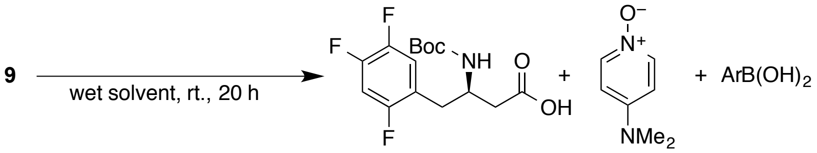

Inert species	Purity [%] in $CDCl_3$		Purity [%] in C_6D_6	
	0 h	after 20 h	0 h	after 20 h
**9** generated from **2** [via entry 1, Table 4]	98	84	>99	88
**9** generated from **4b** [via entry 3, Table 4]	98	41	>99	33
**9** generated from **2** and benzoic acid	90	4	–	–

Furthermore, inert species **9** (generated from **2**, DMAPO and benzoic acid) was also conformed by ^{1}H and ^{11}B NMR analyses. Almost all the inert species in $CDCl_3$ decomposed after 20 h as well as inert species used in Table S1.

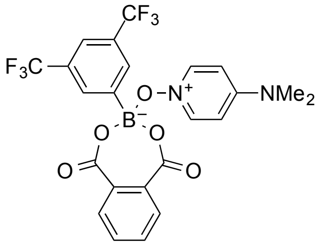

3-(3,5-Bis(trifluoromethyl)phenyl)-3-((4-(dimethylamino)pyridine-1-ium-1-yl)oxy)-1,5-dioxo-1,5-dihydrobenzo[*e*][1,3,2]dioxaborepin-3-uide (9z): ^{1}H NMR ($CDCl_3$, 400 MHz) δ 3.26 (s, 6H), 6.72 (d, J = 8.2 Hz, 2H), 7.33 (dd, J = 3.2, 6.0 Hz, 2H), 7.54 (s, 1H), 7.70 (dd, J = 3.2, 6.0 Hz, 2H), 7.78 (s, 2H), 8.44 (d, J = 7.8 Hz, 2H); ^{11}B NMR (C_6D_6, 128 MHz) δ –13.04; This complex was recrystallized in chloroform at 20 °C for X-ray diffraction analysis. Formula $C_{23}H_{17}BF_6N_2O_5$, colorless, triclinic, space group *P-1 (#2)*, a = 10.3672(14) Å, b = 15.451(2) Å, c = 17.504(2) Å, α = 84.827(6)°, β = 78.441(5)°, γ = 86.992(6)°, V = 2734.1(6) Å^3, Z = 2, ρ_{calc} = 1.568 g/cm^3, λ(MoKα) = 0.71075Å, T = 123 K, 11774 reflections collected, and 743 parameters were used for the solution of the structure. R_1 = 0.0600 and wR_2 = 0.1186. GOF = 1.037. Crystallographic data (excluding structure factors) for the structure reported in this paper have been deposited with Cambridge Crystallographic Data Centre as supplementary publication no. CCDC-1429213. Copies of the data can be obtained free of charge on application to CCDC, 12 Union Road, Cambridge CB2 1EZ, UK [Fax: int. code + 44(1223)336-033; E-mail: deposit@ccdc.cam.ac.uk; Web page: http://www.ccdc.cam.ac.uk/pages/Home.aspx].

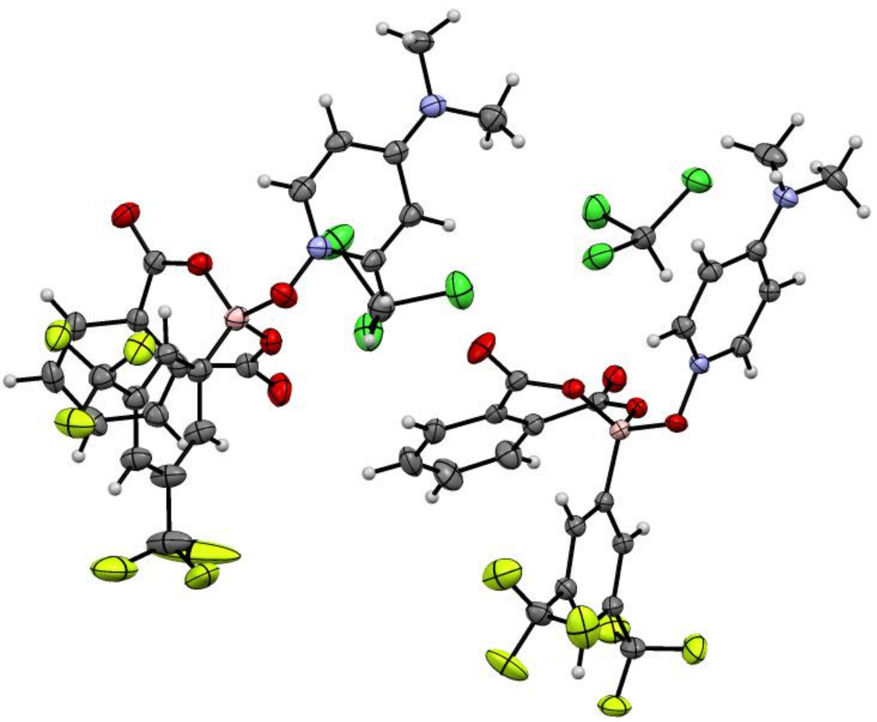

Figure S1. OPTEP drawing of the inert complex. C: gray, H: white, O: red, N: blue, F: lignt green, B: pink, Cl: green.

Table S2. The Dehydrative Condensation between Phthalic Acid and Benzylamine

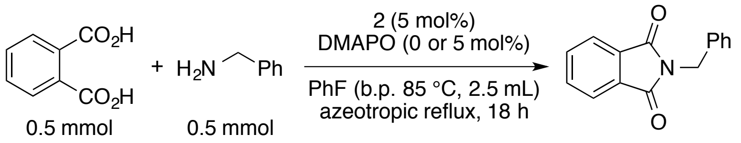

Entry	Catalysts	Yield [%]	
		N-Benzylphthalimide	**9z**
1	**2** (5 mol%)	64	–
2	**2** (5 mol%) + DMAPO (5 mol%)	49	73
3[a]	**9z** (5 mol%)	23	50

[a]0.475 mmol of phthalic acid was used as a substrate because 0.025 mmol of phthalic acid was included in **9z**.

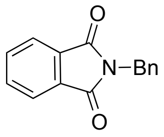

2-Benzylisoindoline-1,3-dione:[16] white solid; ^{1}H NMR (400 MHz, $CDCl_3$) δ 4.85 (s, 2H), 7.24–7.33 (m, 3H), 7.43 (d, J = 7.3 Hz, 2H), 7.68–7.71 (m, 2H), 7.82–7.85 (m, 2H); ^{13}C NMR (100 MHz, $CDCl_3$) δ 41.7, 123.5, 127.9, 128.7, 128.8, 132.2, 134.1, 136.5, 168.2.

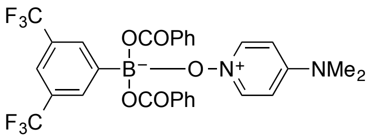

3,5-Bis(trifluoromethyl)phenyl[di(benzoyloxy)]boron•DMAPO: ^{1}H NMR (C_6D_6, 400 MHz) δ 1.66 (s, 6H), 5.20 (d, *J* = 8.2 Hz, 2H), 7.10–7.14 (m, 6H), 7.81 (s, 1H), 8.16 (d, *J* = 7.8 Hz, 2H), 8.41–8.44 (m, 4H), 8.93 (s, 2H); ^{11}B NMR (C_6D_6, 128 MHz) δ –12.32.

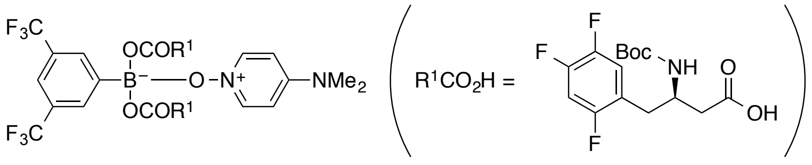

3,5-Bis(trifluoromethyl)phenyl-[di(acyloxy)]boron•DMAPO: ^{1}H NMR ($CDCl_3$, 400 MHz) δ 1.37 (m, 18H), 2.50 (m, 4H), 2.71–2.85 (m, 4H), 3.20 (s, 6H), 4.09 (m, 2H), 5.21–5.31 (m, 2H), 6.66 (m, 2H), 6.82–6.89 (m, 2H), 6.96–7.11 (m, 2H), 7.73 (s, 1H), 8.07 (s, 2H), 8.18 (brd, *J* = 5.5 Hz, 2H); ^{11}B NMR (C_6D_6, 128 MHz) δ –13.96.

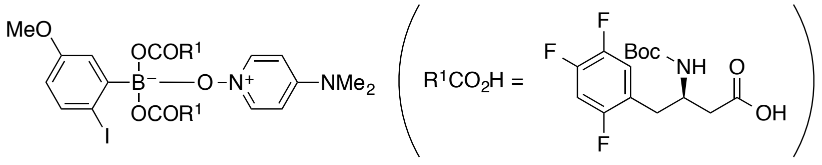

2-Iodo-5-methoxyphenyl[di(acyl-oxy)]-boron•DMAPO: ^{1}H NMR ($CDCl_3$, 400 MHz) δ 1.32 (m, 18H), 2.57 (m, 4H), 2.74–2.88 (m, 4H), 3.18 (s, 6H), 3.80 (s, 3H), 4.11 (m, 2H), 5.37–5.46 (m, 2H), 6.50 (dd, *J* = 3.2, 8.2 Hz, 1H), 6.63 (m, 2H), 6.81–6.90 (m, 2H), 6.97–7.14 (m, 2H), 7.27 (s, 1H), 7.70 (d, *J* = 8.2 Hz, 1H), 8.30 (brd, *J* = 5.5 Hz, 2H); ^{11}B NMR (C_6D_6, 128 MHz) δ –13.72.

Inert species **9** generated from **2** (via entry 1, Table 4) was not converted to desired amide product directly under the conditions of amide synthesis as shown in Scheme S2: A solution of **9** (0.039 mmol) and 3-(trifluoromethyl)-5,6,7,8-tetrahydro-[1,2,4]triazolo[4,3-*a*]pyrazine (0.039 mmol) in fluorobenzene was heated under azeotropic reflux conditions with removal of water for 3 h. As a result, **9** was recovered, and no amide product was generated.

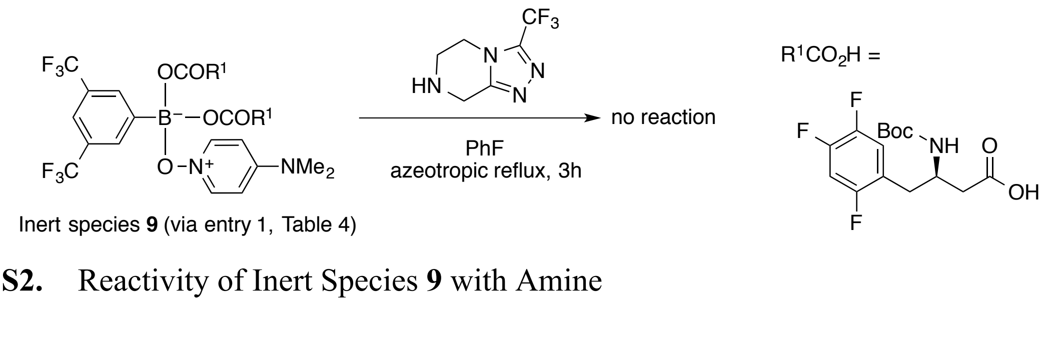

Scheme S2. Reactivity of Inert Species **9** with Amine

Control Experiments for the Chemoselective Dehydrative Condensation of Unsaturated Carboxylic Acids

The unsaturated amide **10e** didn't react with 2-phenylethylamine to give the β-amino amide **11e**. It was ascertained that **11e** was selectively given from β-aminocarboxylic acid **14e** quantitatively.

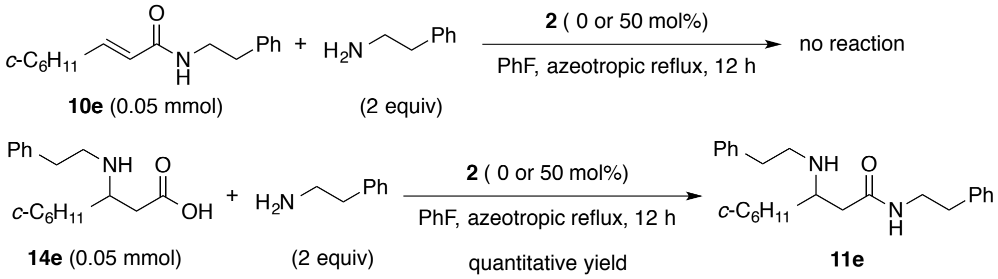

Scheme S3. Reactivity of **10e** and **14e** with 2-Phenylethylamine

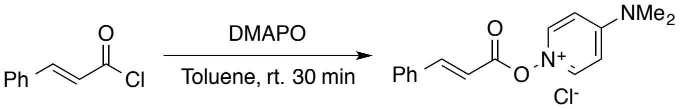

To a solution of cinnamoyl chloride (8.3 mg, 0.05 mmol) in dry toluene (2 mL) was added DMAPO (7 mg, 0.05 mmol). After the resulting mixture was allowed stirring at room temperature for 30 min, solvent was removed to give the crude product *in vacuo*. Thus, the generation of acyloxypyridinium species was confirmed by ^{1}H NMR study. ^{1}H NMR ($CDCl_3$, 400 MHz) δ 3.26 (s, 3H), 7.04 (d, *J* = 16.5 Hz, 1H), 7.18 (d, *J* = 7.8 Hz, 2H), 7.04–7.54 (m, 5H), 7.91 (d, *J* = 7.3 Hz, 2H), 8.06 (d, *J* = 16.0 Hz, 1H), 8.74 (d, *J* = 7.3 Hz, 2H).

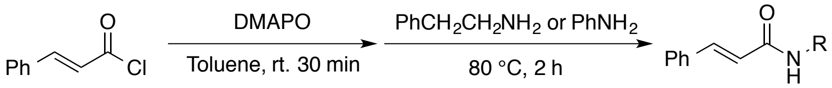

To a solution of cinnamoyl chloride (16.7 mg, 0.10 mmol) in dry toluene (2 mL) was added DMAPO (14 mg, 0.10 mmol). The resulting mixture was allowed stirring at room temperature for 30 min. Then the mixture was heated to 80 °C, after 5 minutes, 2-phenylethylamine (0.20 mmol, 25 mL) or aniline (0.2 mmol, 19 mL) was added dropwise, and then heated at 80 °C for 2 h. After reaction cooling to room temperature, 1 mL of brine was added, the organic phase was separated and dried over anhydrous Na_2SO_4. Removing the solvent to give the crude product. ^{1}H NMR analysis showed that only direct amide product was obtained.

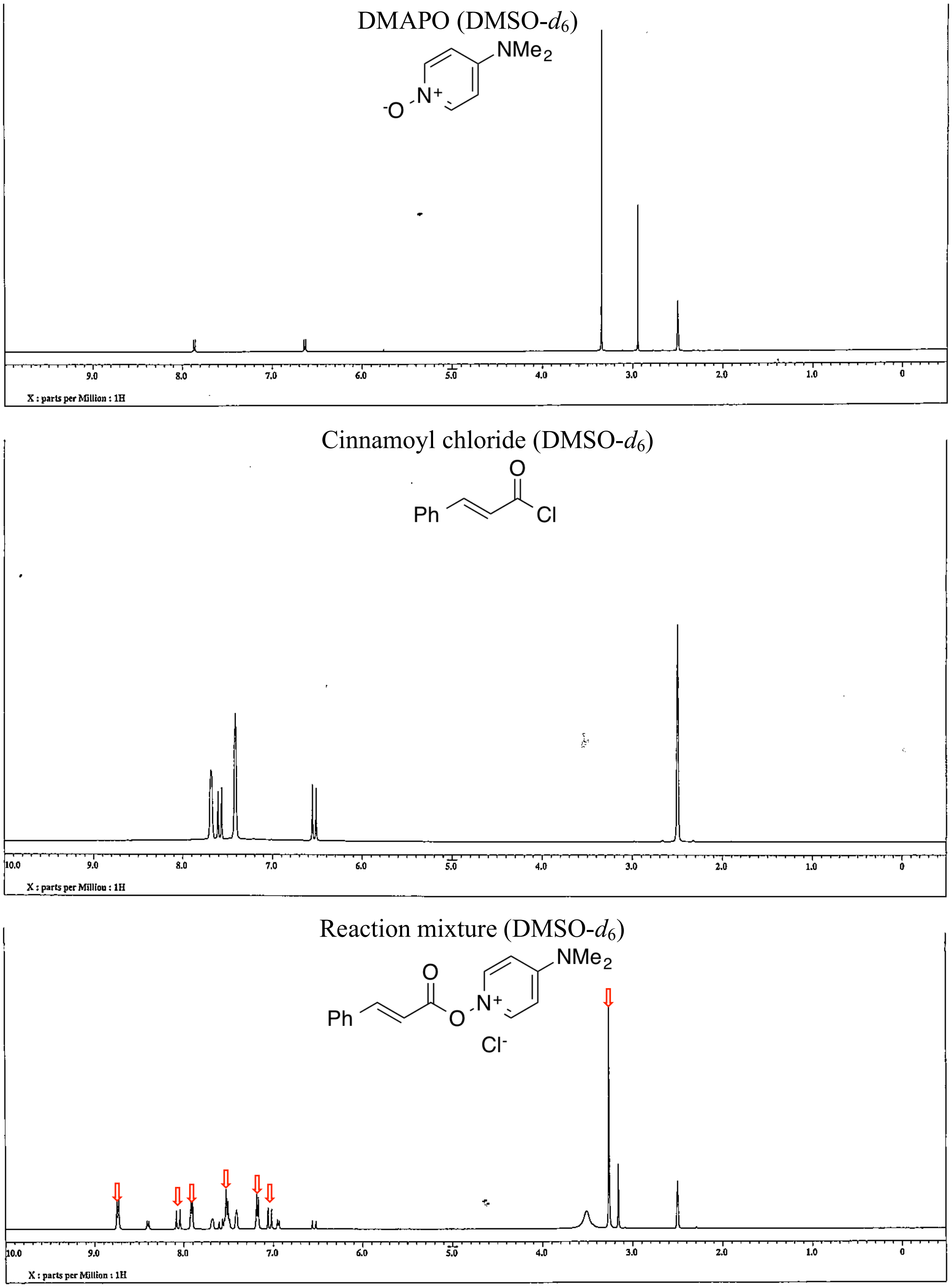

Figure S1. NMR Spectra for control experiments.

References and Notes

1. K. Arnold, B. Davies, R. L.Giles, C. Grosjean, G. E. Smith, A. Whiting *Adv. Synth. Catal.* **2006**, *348*, 813–820.
2. N. Germigon, R. M. Al-Zoubi, D. G. Hall *J. Org. Chem.* **2012**, *77*, 8386–8400.
3. I. Shina, H. Ushiyama, Y. Yamada, Y. Kawakita, K. Nakata *Chem. Asian J.* **2008**, *3*, 454–461.
4. D. Lafrance, P. Bowles, K. Leeman, R. Rafka *Org. Lett.* **2011**, *13*, 2322–2325.
5. Commercially available from Aldrich.
6. Y. Tsuji, T. Ohsumi, T. Kondo, Y. Watanabe *J. Organomet. Chem.* **1986**, *309*, 333–344.
7. T. Maki, K. Ishihara, H. Yamamoto *Org. Lett.* **2006**, *8*, 1431–1434.
8. A. Gissot, A. Volonterio, M. Zanda *J. Org. Chem*. **2005**, *70*, 6925–6928.
9. C. A. Faler, M. M. Joullié *Tetrahedron Lett.* **2006**, *47*, 7229–7231.
10. W. Yoo, C. Li *J. Am. Chem. Soc.* **2006**, *128*, 13064–13065.
11. D. Kim, L. Wang, M. Beconi, G. J. Eiermann, M. H. Fisher, H. He, G. J. Hickey, J. E. Kowalchick, B. Leiting, K. Lyons, F. Marsilio, M. E. McCann, R. A. Patel, A. Petrov, G. Scapin, S. B. Patel, R. S. Roy, J. K. Wu, M. J. Wyvratt, B. B. Zhang, L. Zhu, N. A. Thornberry, A. E. Weber *J. Med. Chem.* **2005**, *48*, 141–151.
12. K. M. Driller, S. Prateeptongkum, R. Jackstell, M. Beller *Angew. Chem. Int. Ed.* **2011**, *50*, 537–541.
13. Q. Dong, G. Liu, H. Zhou, L. Chen, Z. Yao *Tetrahedron Lett.* **2008**, *49*, 1636–1640.
14. J. B. Leslie, R. C. Holaday, T. Nguyen, P. J. Hergenrother *J. Med. Chem*. **2010**, *53*, 3964–3972.
15. S. Das, D. Addis, S. Zhou, K. Junge, M. Beller *J. Am. Chem. Soc.* **2010**, 132, 1770–1771.
16. J-C. Hsieh, C-H. Cheng *Chem. Commun.* **2005**, *36*, 4554–4556.

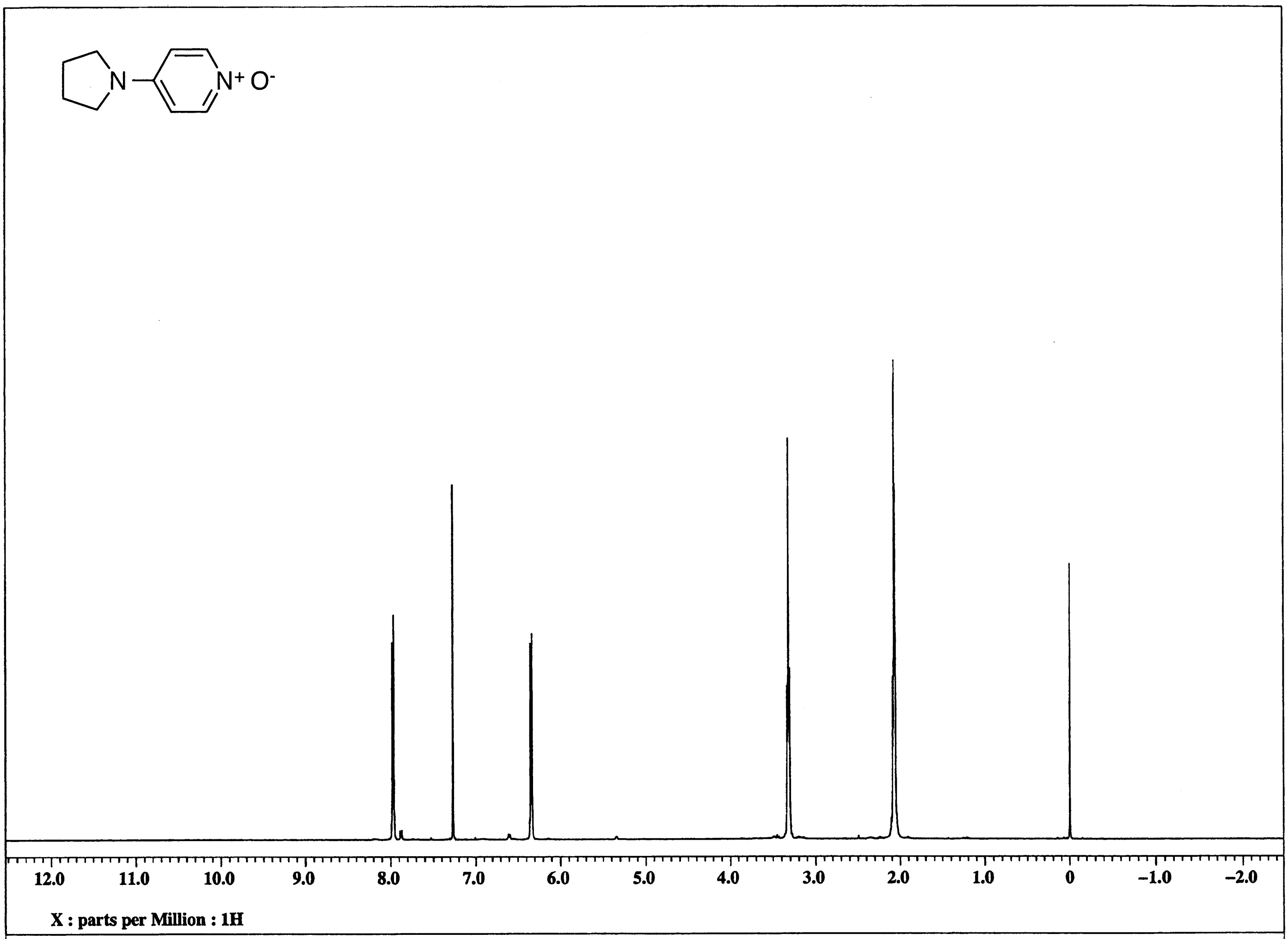
N
N+ O-
12.0 11.0 10.0 9.0 8.0 7.0 6.0 5.0 4.0 3.0 2.0 1.0 0 −1.0 −2.0
X : parts per Million : 1H

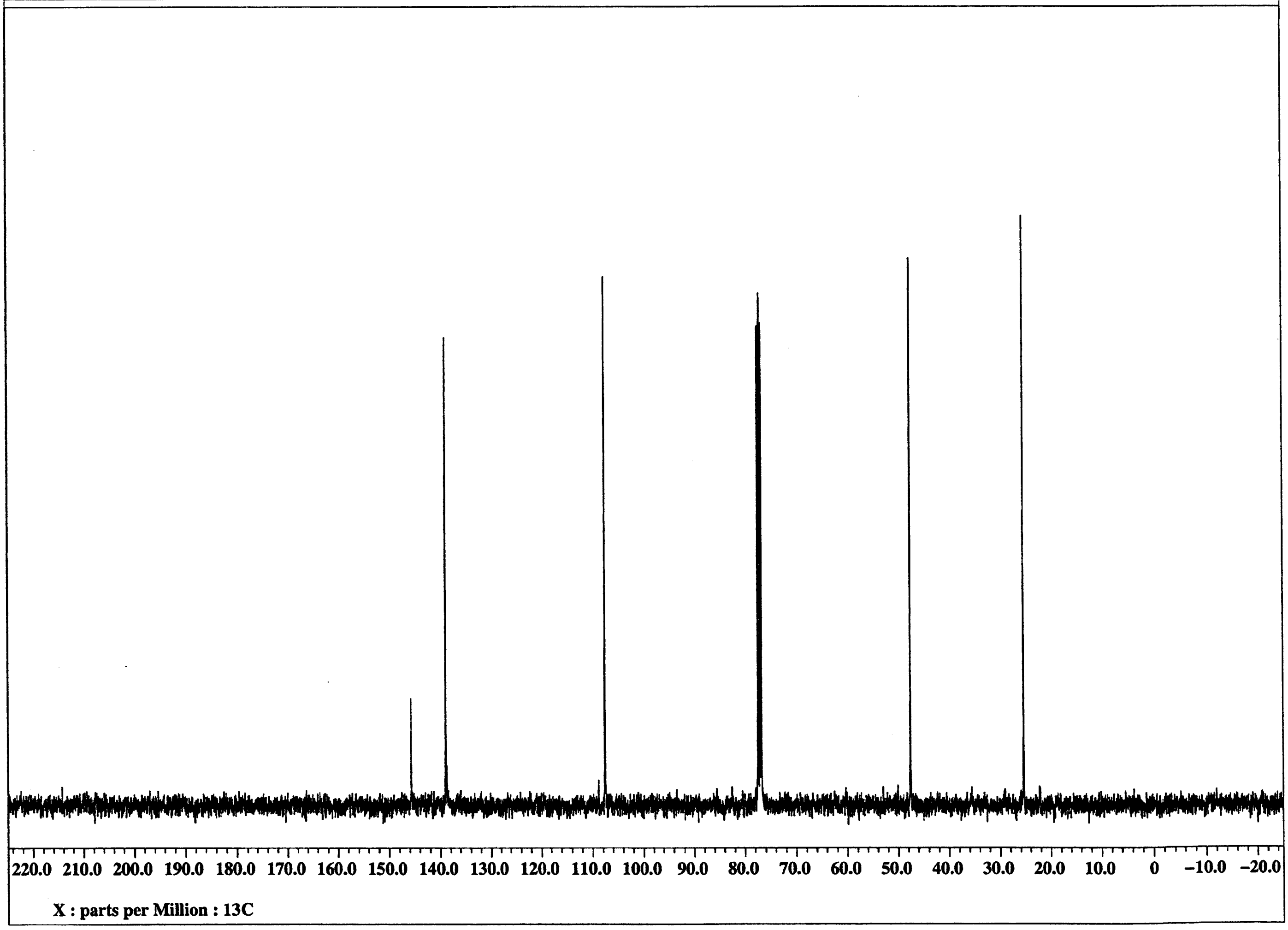
220.0 210.0 200.0 190.0 180.0 170.0 160.0 150.0 140.0 130.0 120.0 110.0 100.0 90.0 80.0 70.0 60.0 50.0 40.0 30.0 20.0 10.0 0 −10.0 −20.0
X : parts per Million : 13C

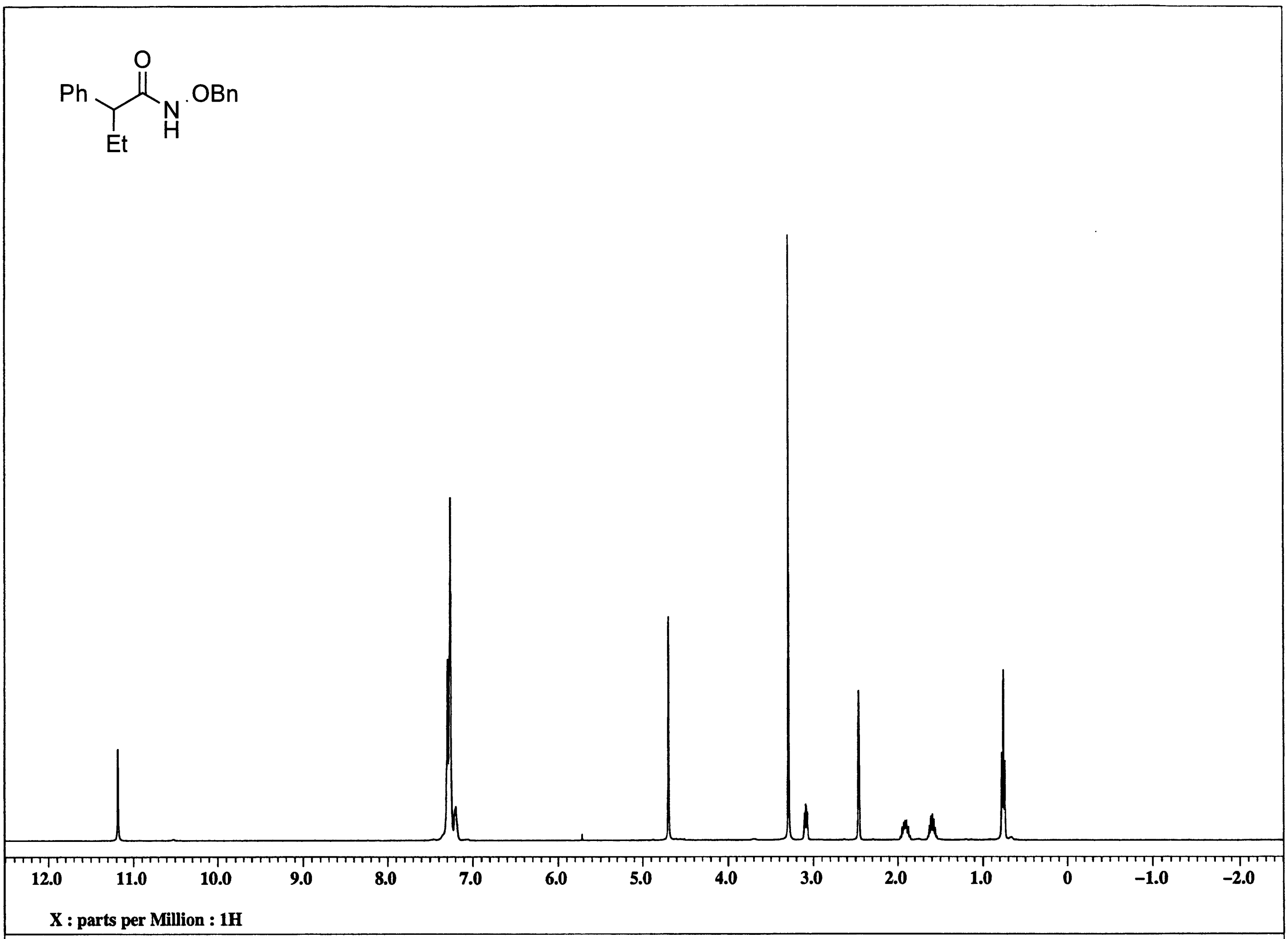
Ph
O
N
OBn
H
Et
12.0 11.0 10.0 9.0 8.0 7.0 6.0 5.0 4.0 3.0 2.0 1.0 0 −1.0 −2.0
X : parts per Million : 1H

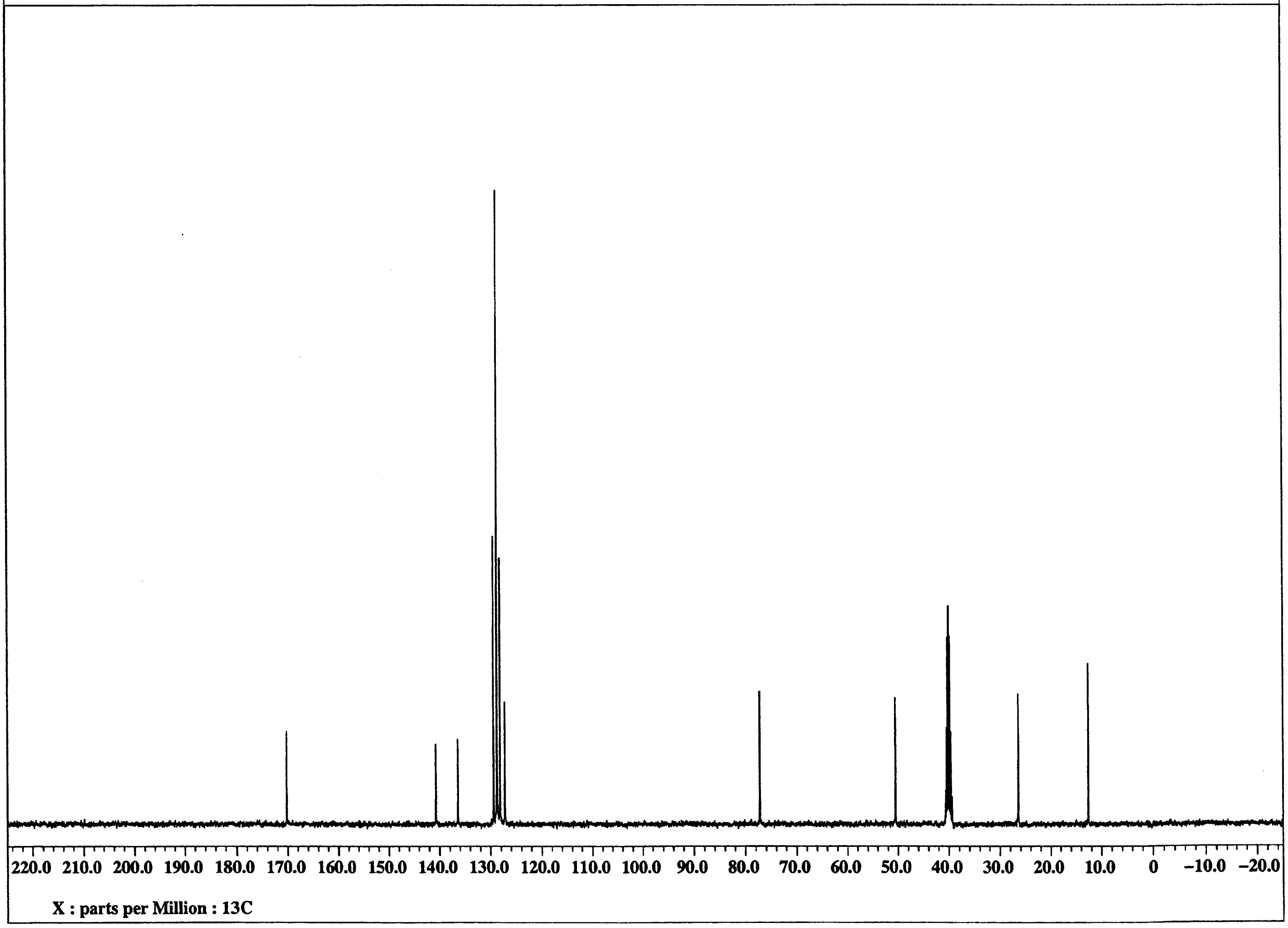
220.0 210.0 200.0 190.0 180.0 170.0 160.0 150.0 140.0 130.0 120.0 110.0 100.0 90.0 80.0 70.0 60.0 50.0 40.0 30.0 20.0 10.0 0 −10.0 −20.0
X : parts per Million : 13C

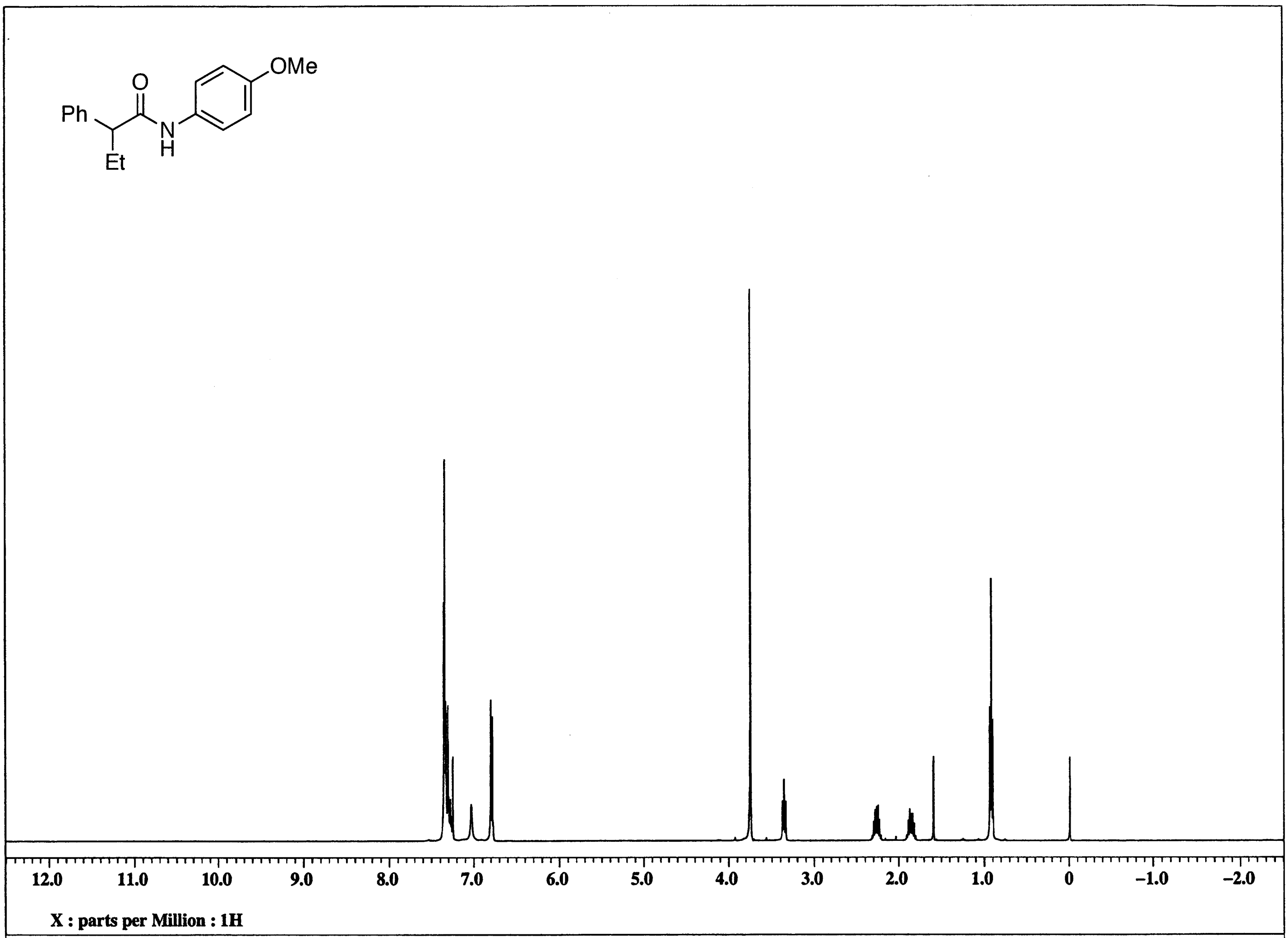

OMe
O
Ph
N
H
Et
12.0 11.0 10.0 9.0 8.0 7.0 6.0 5.0 4.0 3.0 2.0 1.0 0 −1.0 −2.0
X : parts per Million : 1H

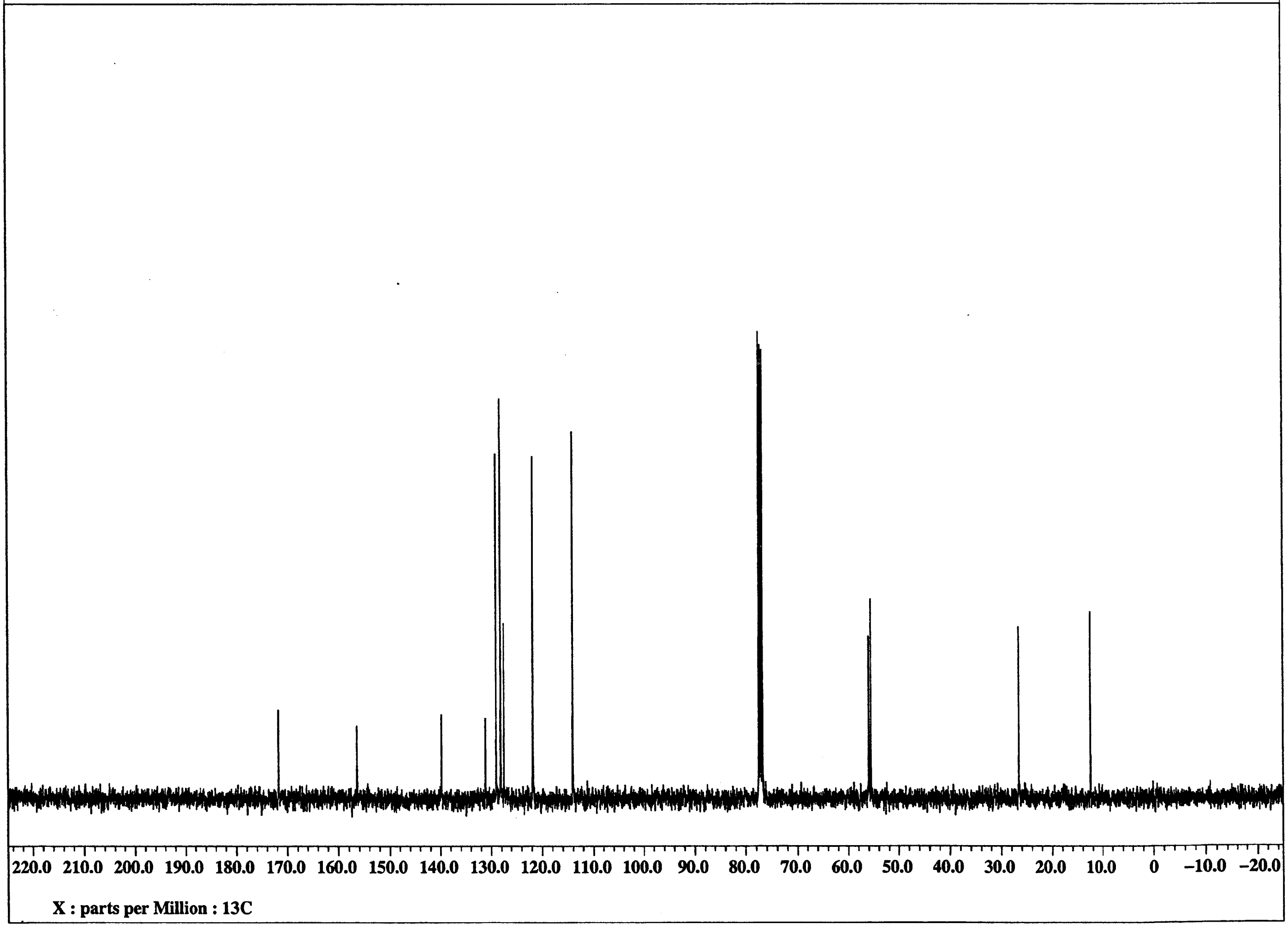

220.0 210.0 200.0 190.0 180.0 170.0 160.0 150.0 140.0 130.0 120.0 110.0 100.0 90.0 80.0 70.0 60.0 50.0 40.0 30.0 20.0 10.0 0 −10.0 −20.0
X : parts per Million : 13C

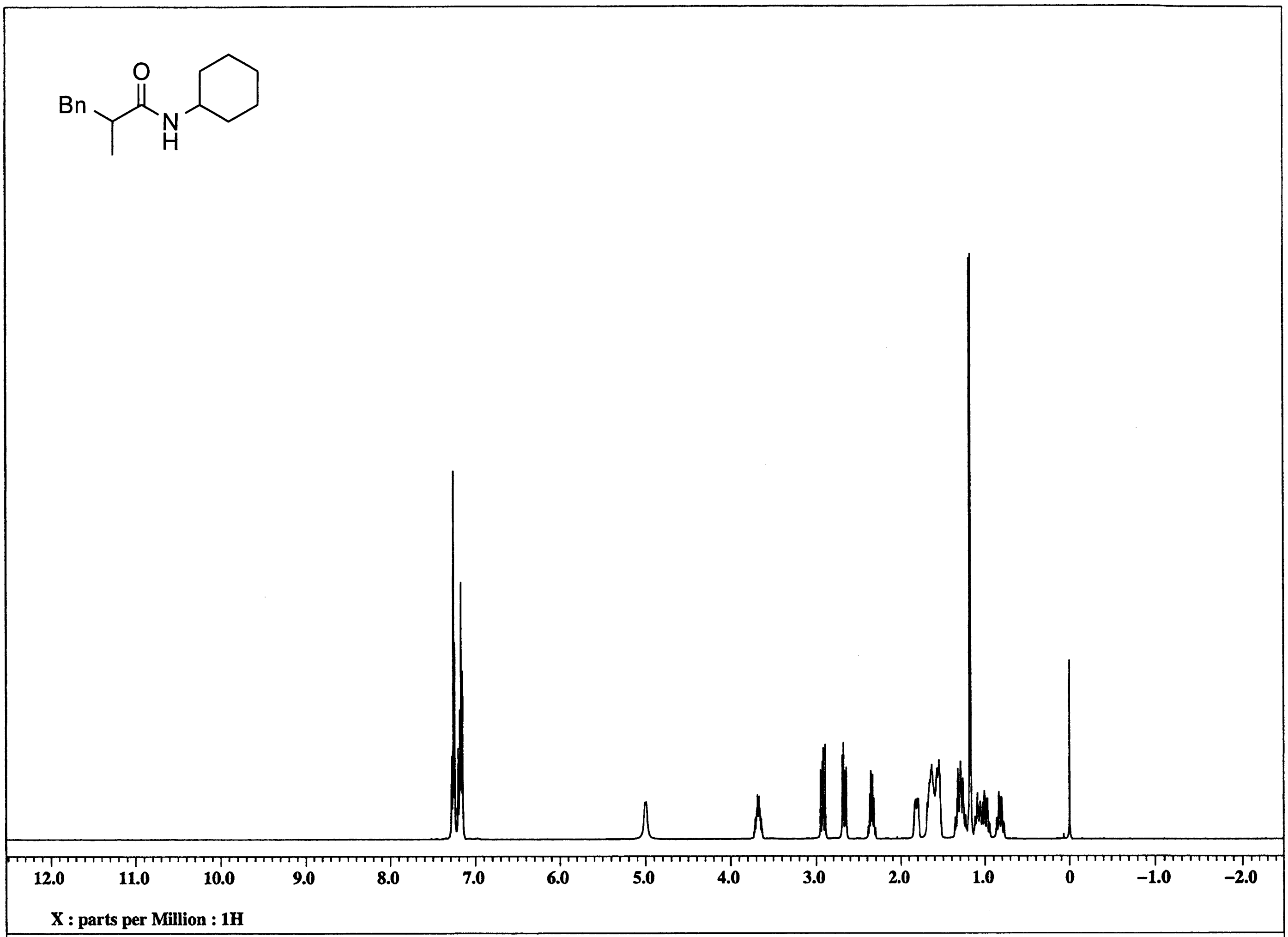
Bn
O
N
H
12.0 11.0 10.0 9.0 8.0 7.0 6.0 5.0 4.0 3.0 2.0 1.0 0 −1.0 −2.0
X : parts per Million : 1H

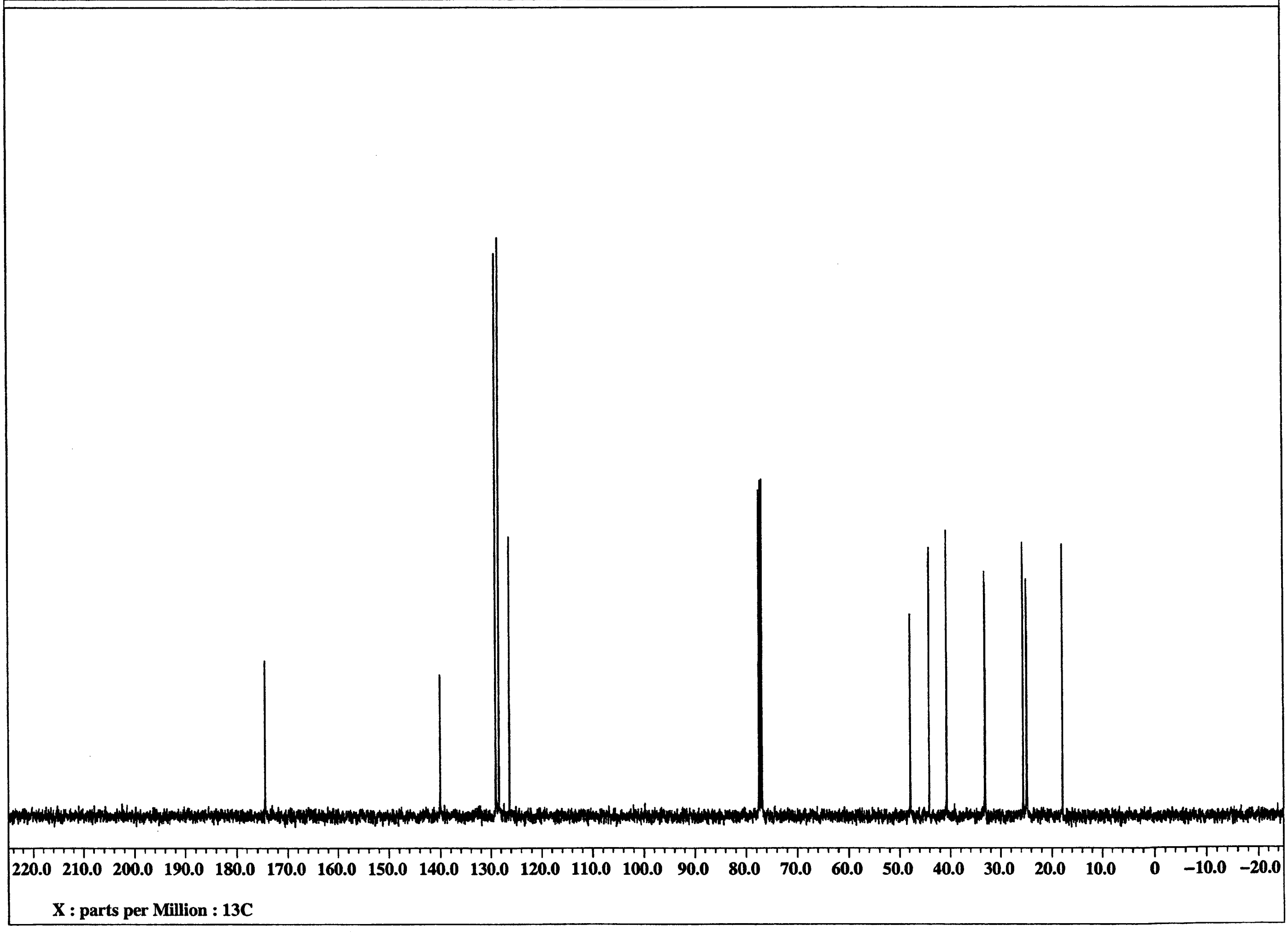
220.0 210.0 200.0 190.0 180.0 170.0 160.0 150.0 140.0 130.0 120.0 110.0 100.0 90.0 80.0 70.0 60.0 50.0 40.0 30.0 20.0 10.0 0 −10.0 −20.0
X : parts per Million : 13C

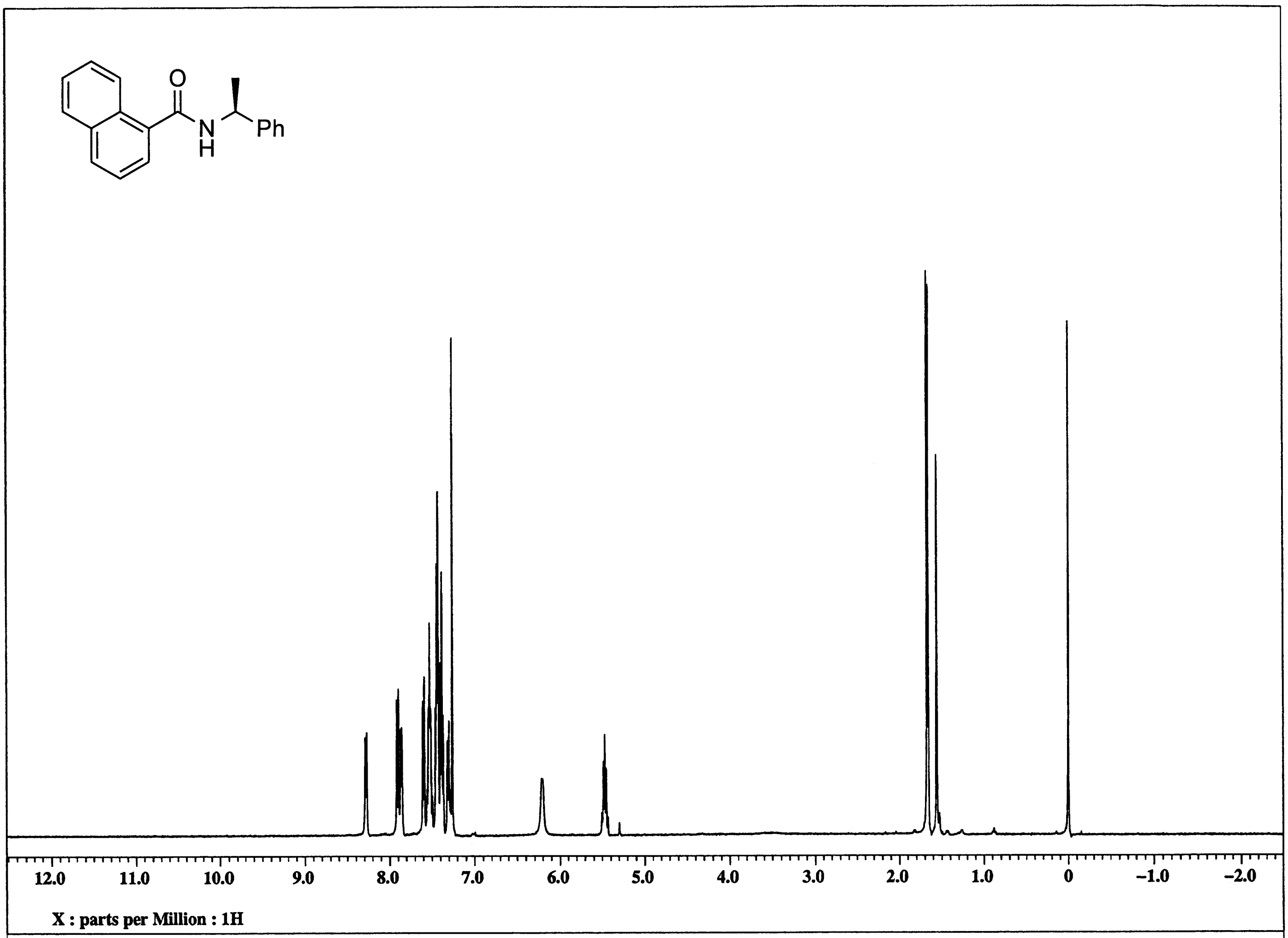
Ph
12.0 11.0 10.0 9.0 8.0 7.0 6.0 5.0 4.0 3.0 2.0 1.0 0 −1.0 −2.0
X : parts per Million : 1H

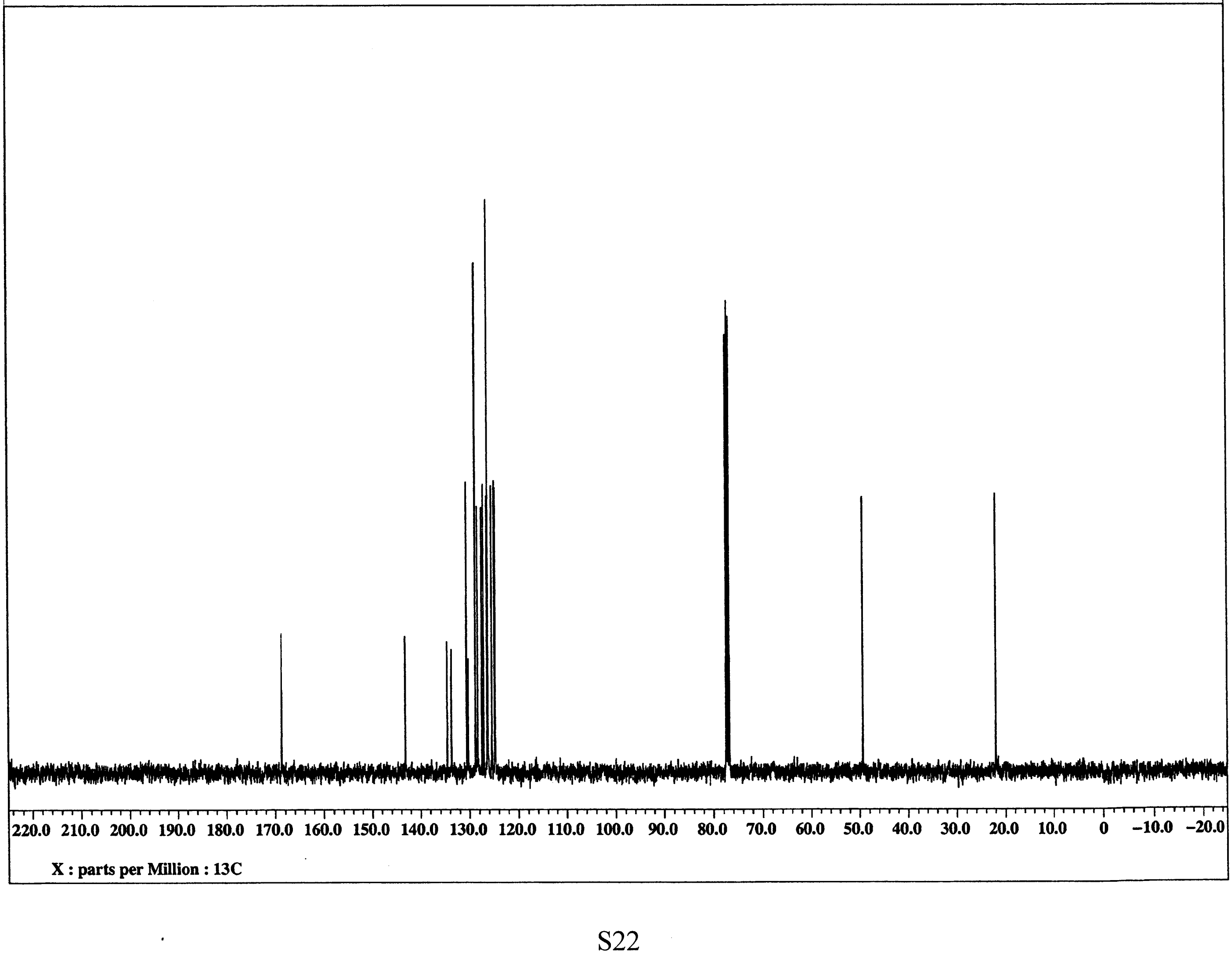
220.0 210.0 200.0 190.0 180.0 170.0 160.0 150.0 140.0 130.0 120.0 110.0 100.0 90.0 80.0 70.0 60.0 50.0 40.0 30.0 20.0 10.0 0 −10.0 −20.0
X : parts per Million : 13C

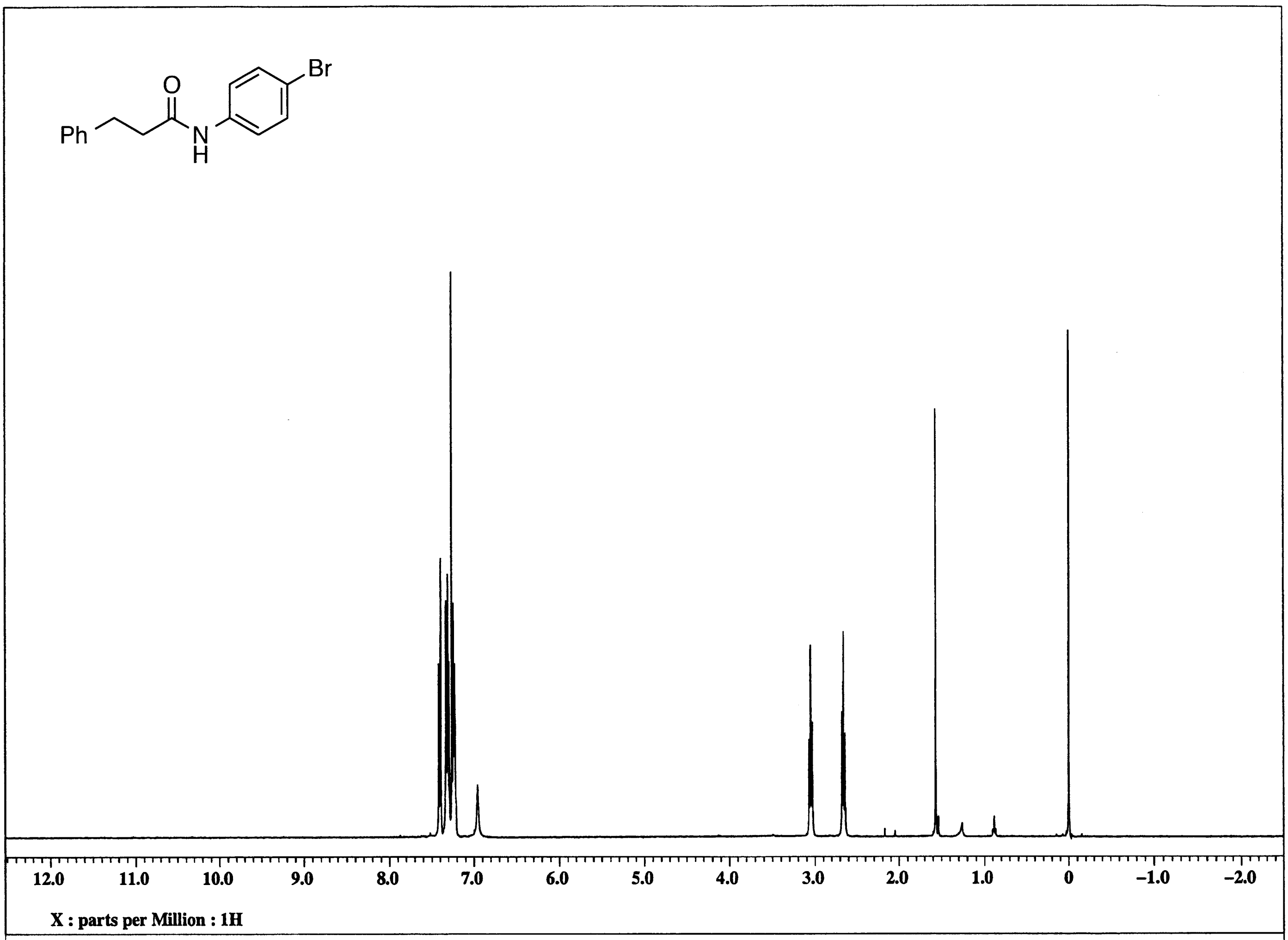
Ph
O
N
H
Br
12.0 11.0 10.0 9.0 8.0 7.0 6.0 5.0 4.0 3.0 2.0 1.0 0 −1.0 −2.0
X : parts per Million : 1H

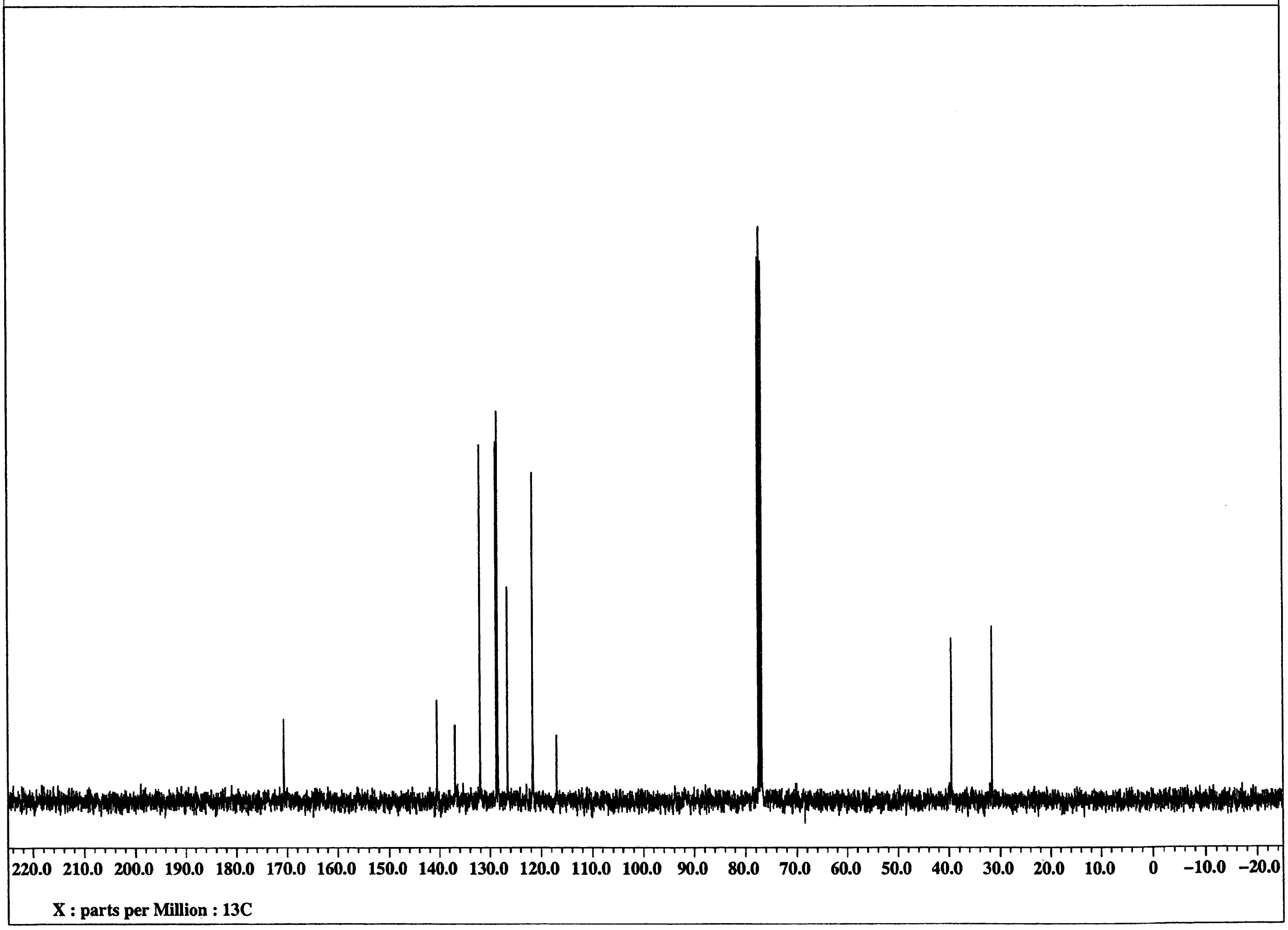
220.0 210.0 200.0 190.0 180.0 170.0 160.0 150.0 140.0 130.0 120.0 110.0 100.0 90.0 80.0 70.0 60.0 50.0 40.0 30.0 20.0 10.0 0 −10.0 −20.0
X : parts per Million : 13C

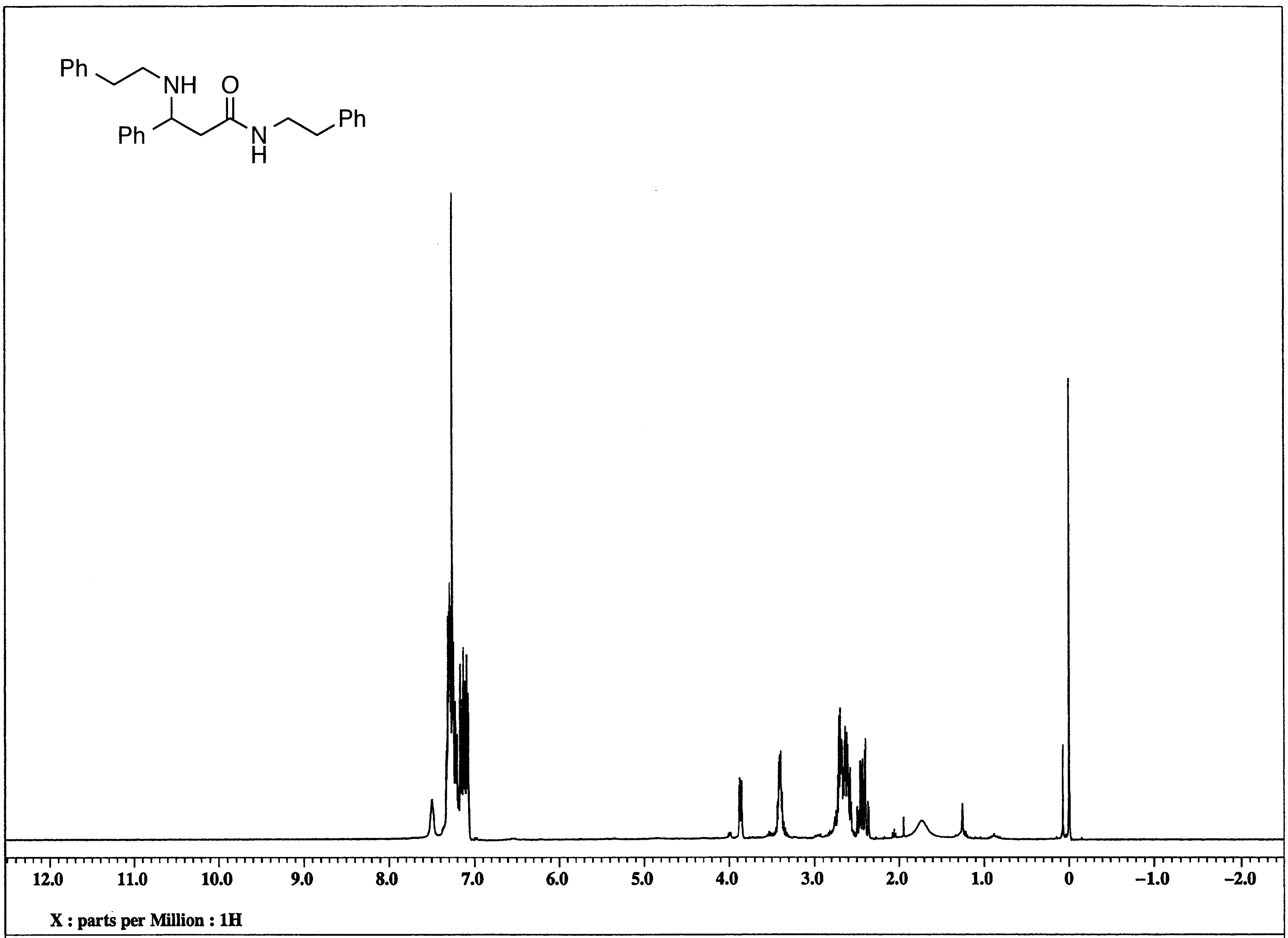
Ph
NH
O
Ph
N
H
Ph
12.0 11.0 10.0 9.0 8.0 7.0 6.0 5.0 4.0 3.0 2.0 1.0 0 −1.0 −2.0
X : parts per Million : 1H

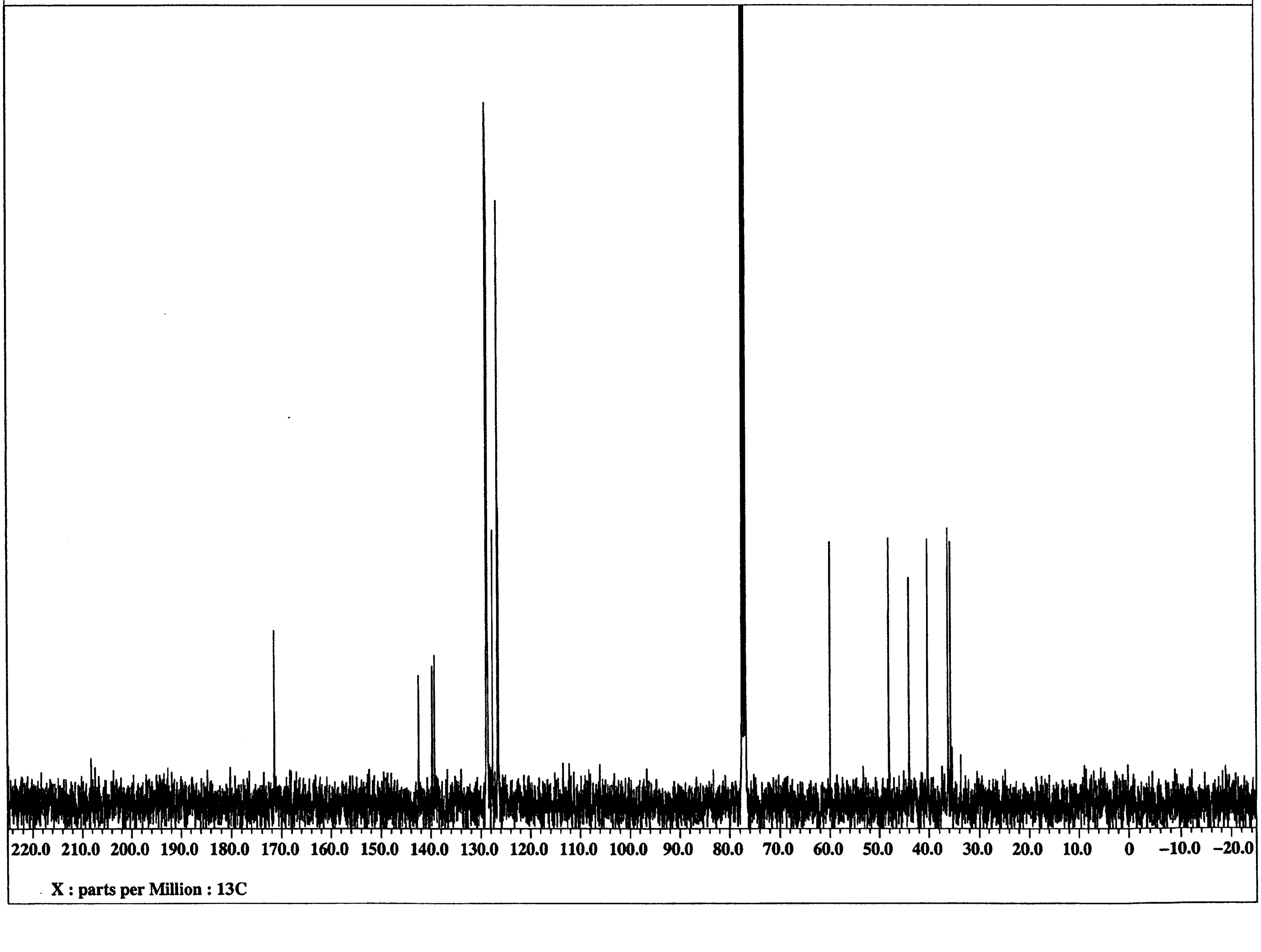
220.0 210.0 200.0 190.0 180.0 170.0 160.0 150.0 140.0 130.0 120.0 110.0 100.0 90.0 80.0 70.0 60.0 50.0 40.0 30.0 20.0 10.0 0 −10.0 −20.0
X : parts per Million : 13C

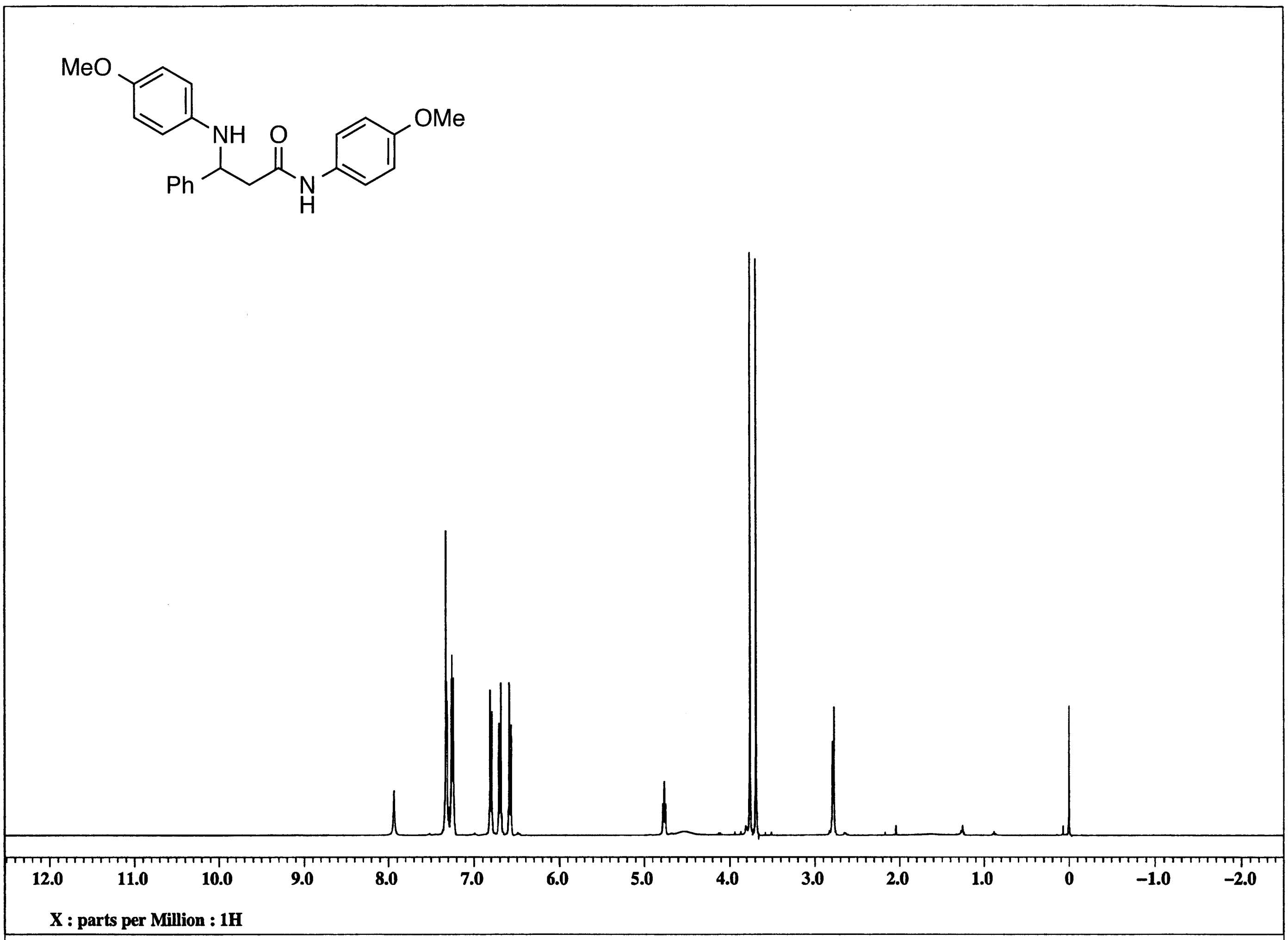
MeO
NH
O
OMe
Ph
N
H
12.0 11.0 10.0 9.0 8.0 7.0 6.0 5.0 4.0 3.0 2.0 1.0 0 −1.0 −2.0
X : parts per Million : 1H

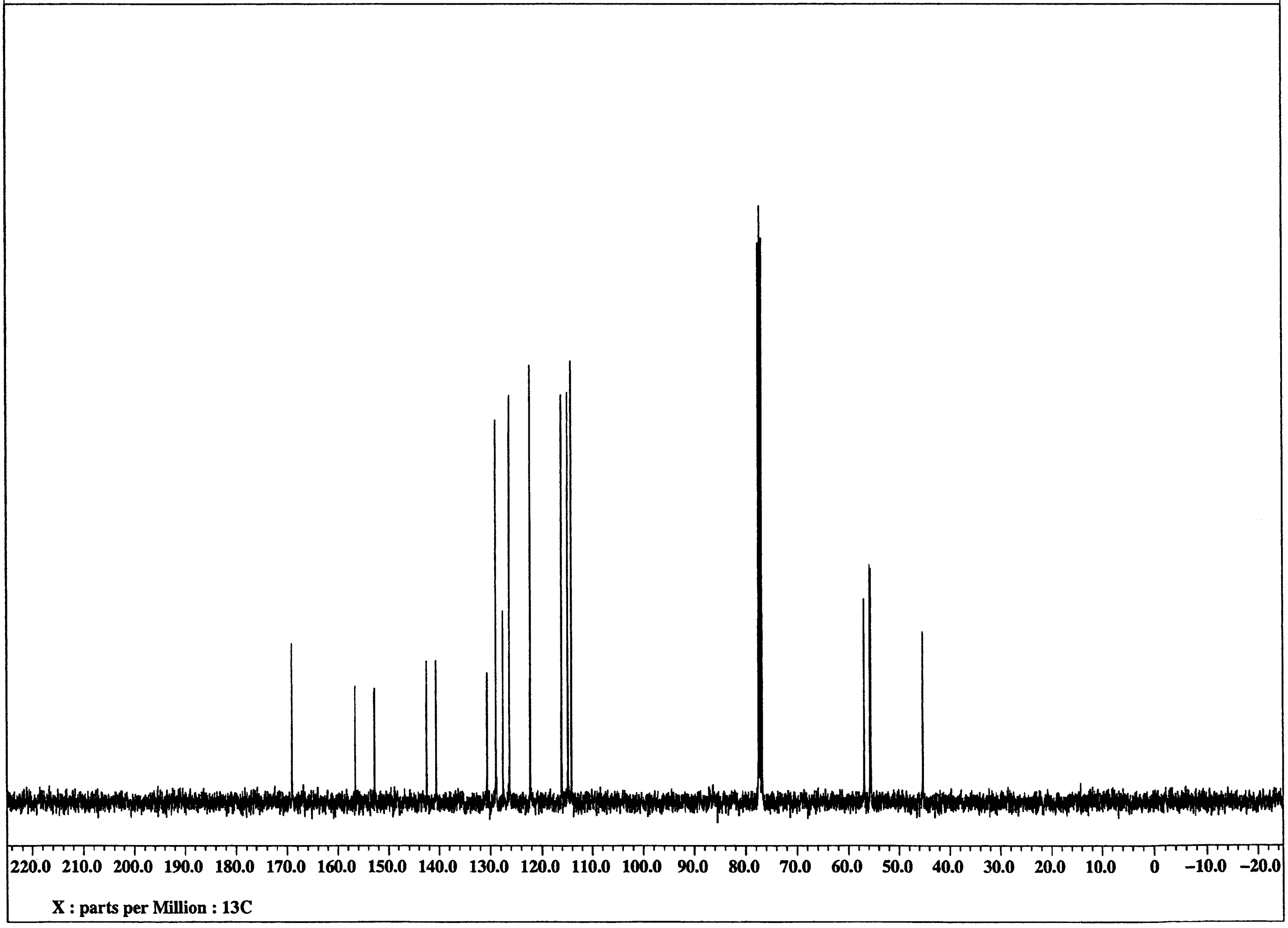
220.0 210.0 200.0 190.0 180.0 170.0 160.0 150.0 140.0 130.0 120.0 110.0 100.0 90.0 80.0 70.0 60.0 50.0 40.0 30.0 20.0 10.0 0 −10.0 −20.0
X : parts per Million : 13C

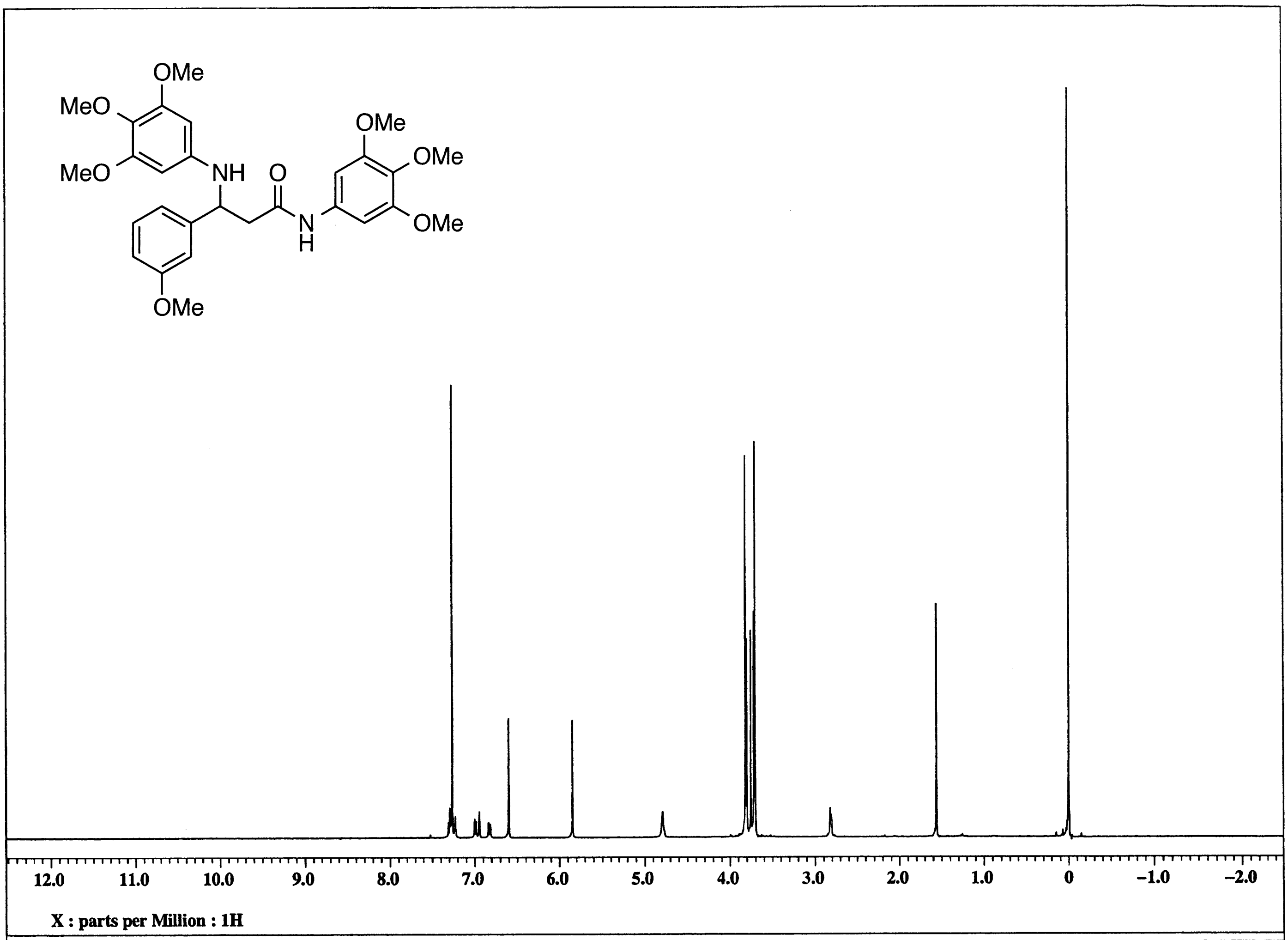
OMe
MeO
MeO
NH
O
OMe
OMe
N
H
OMe
OMe
12.0
11.0
10.0
9.0
8.0
7.0
6.0
5.0
4.0
3.0
2.0
1.0
0
−1.0
−2.0
X : parts per Million : 1H

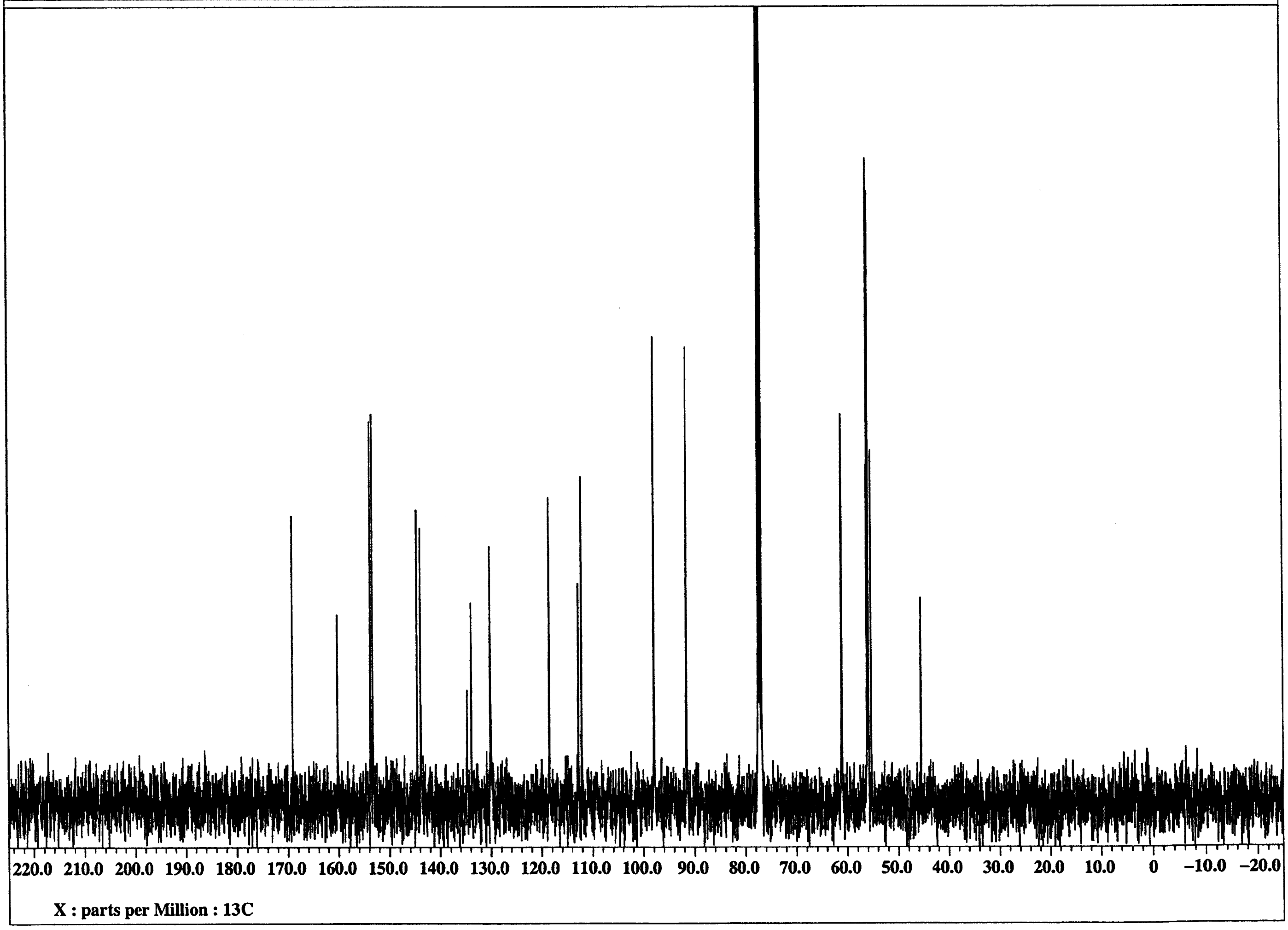
220.0
210.0
200.0
190.0
180.0
170.0
160.0
150.0
140.0
130.0
120.0
110.0
100.0
90.0
80.0
70.0
60.0
50.0
40.0
30.0
20.0
10.0
0
−10.0
−20.0
X : parts per Million : 13C

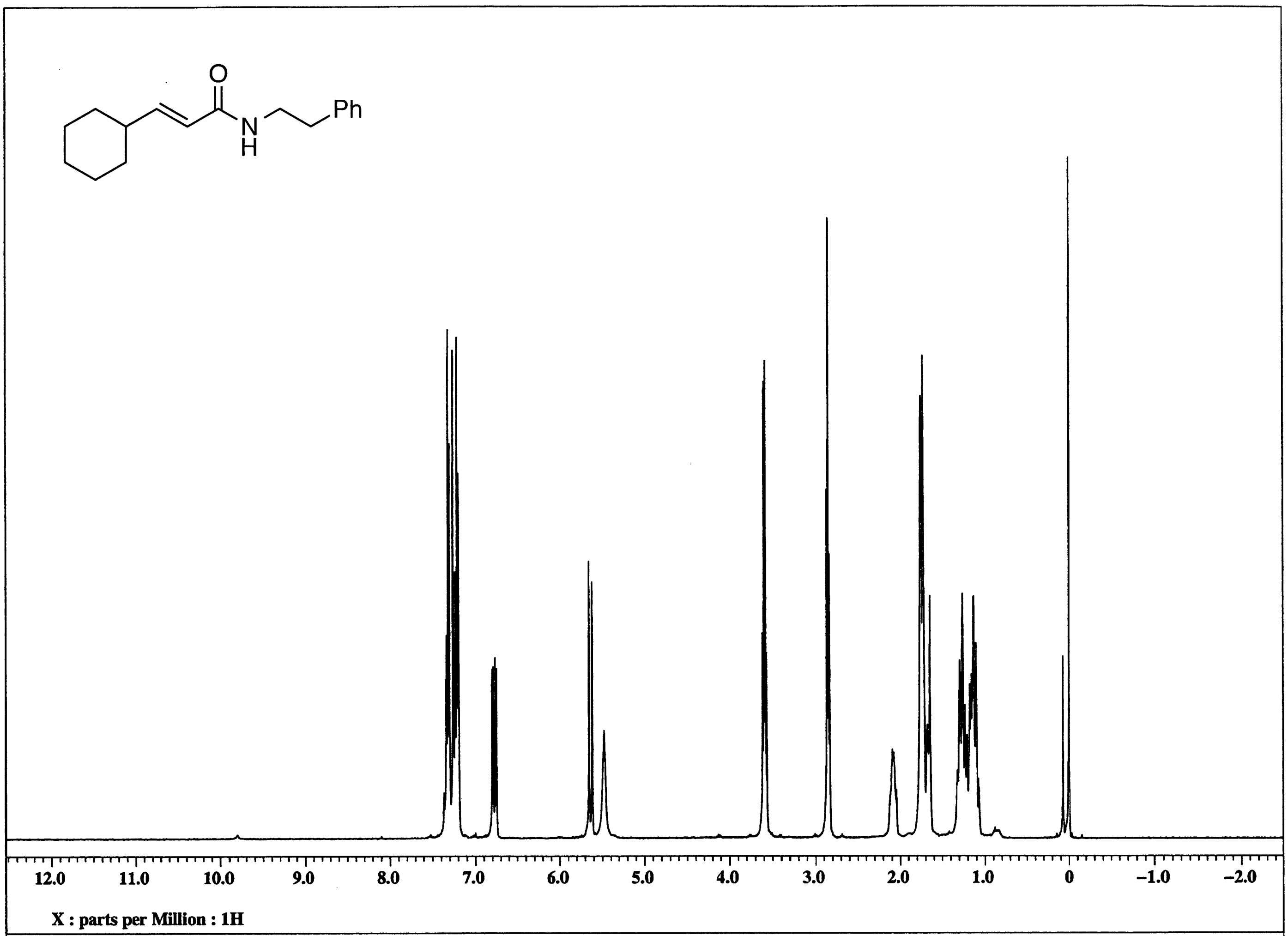
O
N
H
Ph
12.0 11.0 10.0 9.0 8.0 7.0 6.0 5.0 4.0 3.0 2.0 1.0 0 −1.0 −2.0
X : parts per Million : 1H

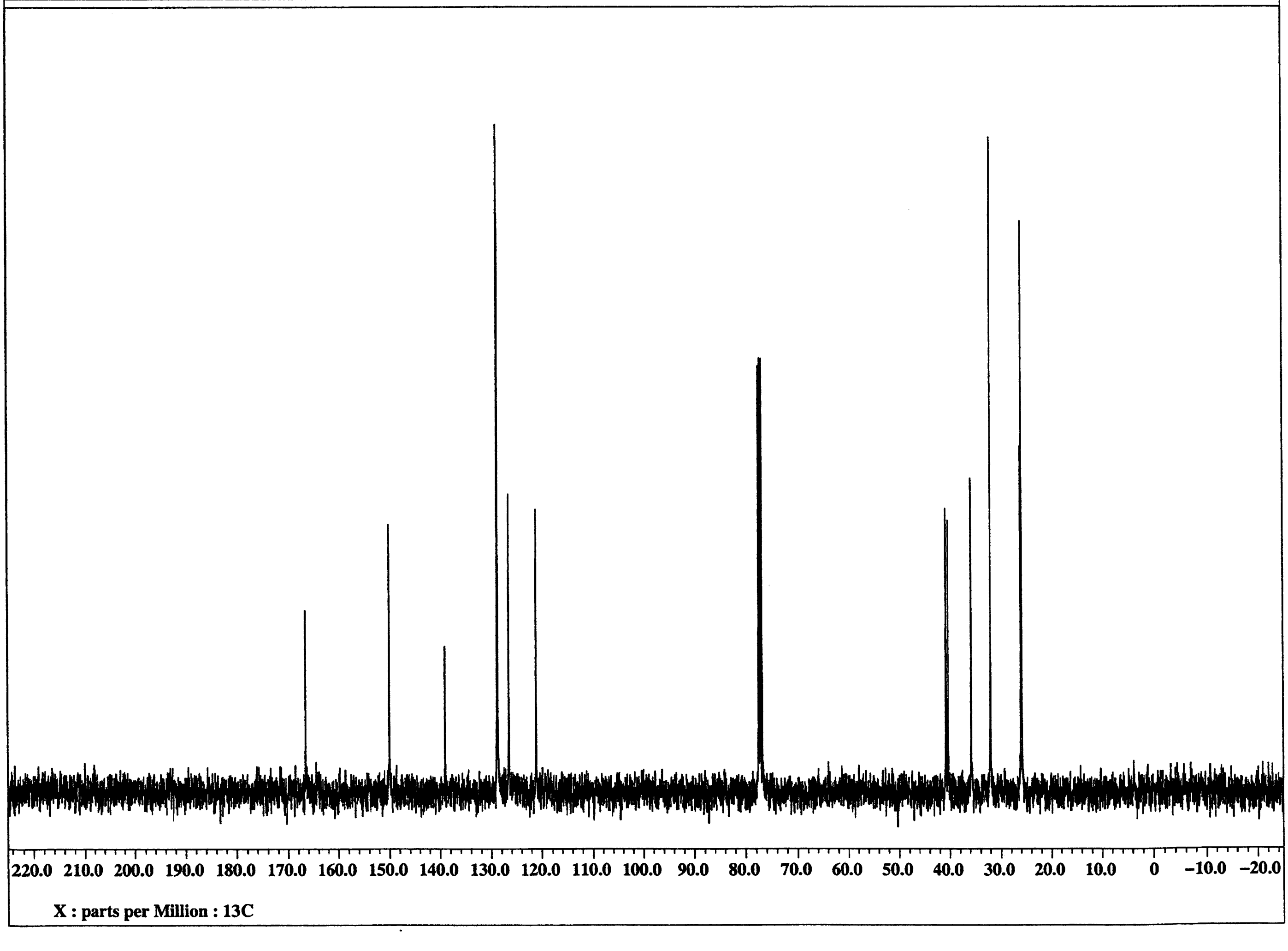
220.0 210.0 200.0 190.0 180.0 170.0 160.0 150.0 140.0 130.0 120.0 110.0 100.0 90.0 80.0 70.0 60.0 50.0 40.0 30.0 20.0 10.0 0 −10.0 −20.0
X : parts per Million : 13C

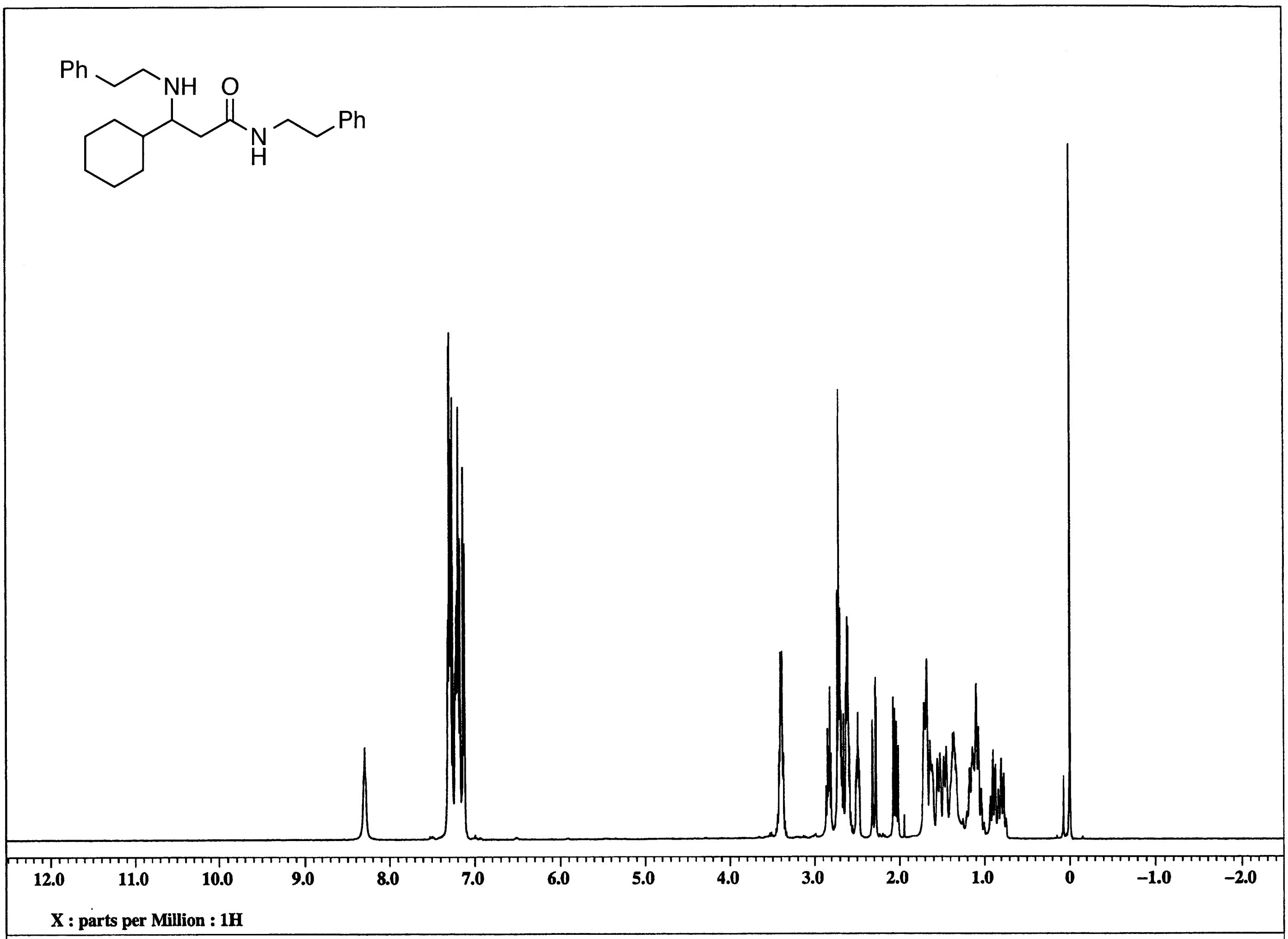
Ph
NH
O
N
H
Ph
12.0 11.0 10.0 9.0 8.0 7.0 6.0 5.0 4.0 3.0 2.0 1.0 0 −1.0 −2.0
X : parts per Million : 1H

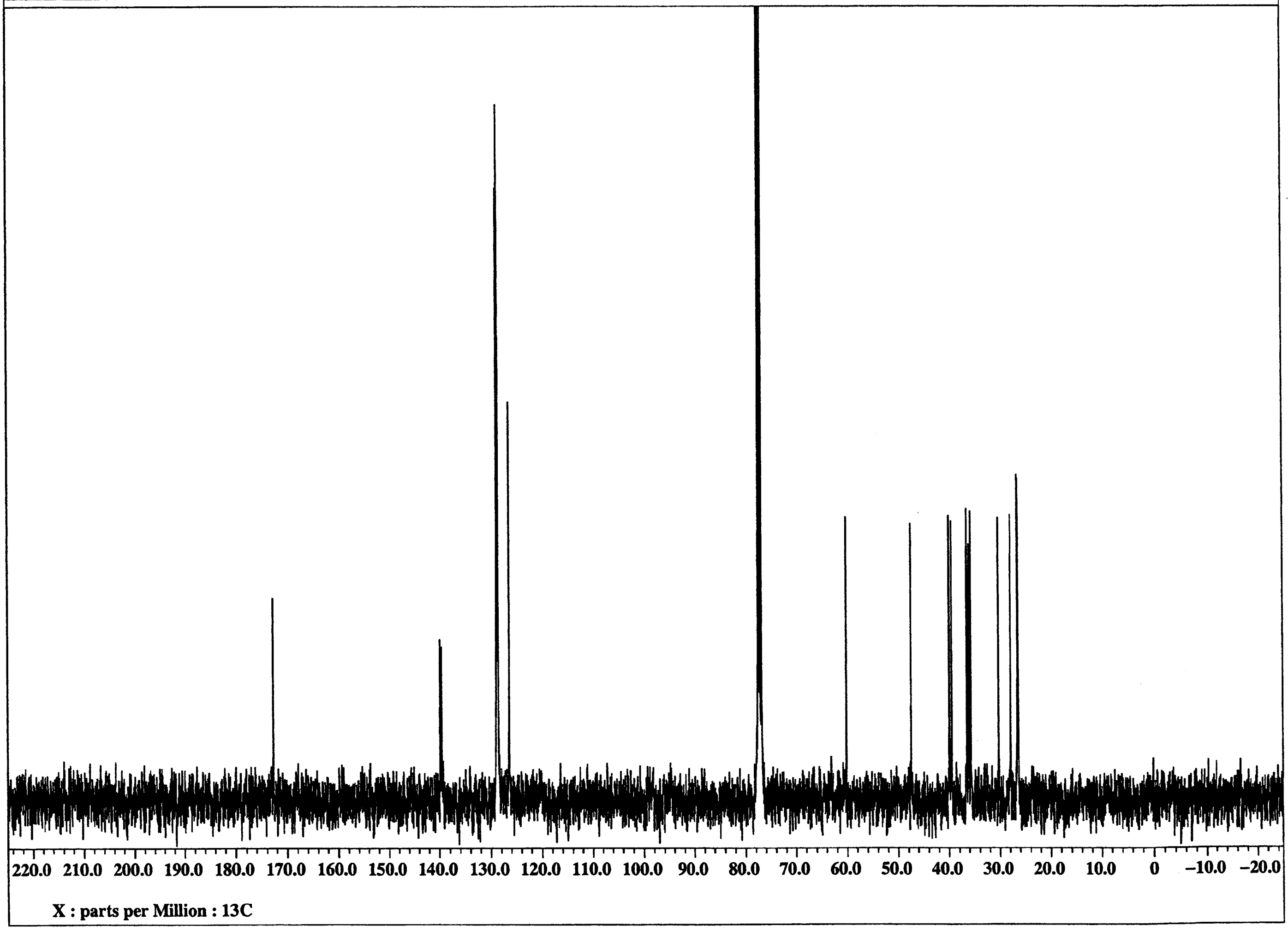
220.0 210.0 200.0 190.0 180.0 170.0 160.0 150.0 140.0 130.0 120.0 110.0 100.0 90.0 80.0 70.0 60.0 50.0 40.0 30.0 20.0 10.0 0 −10.0 −20.0
X : parts per Million : 13C

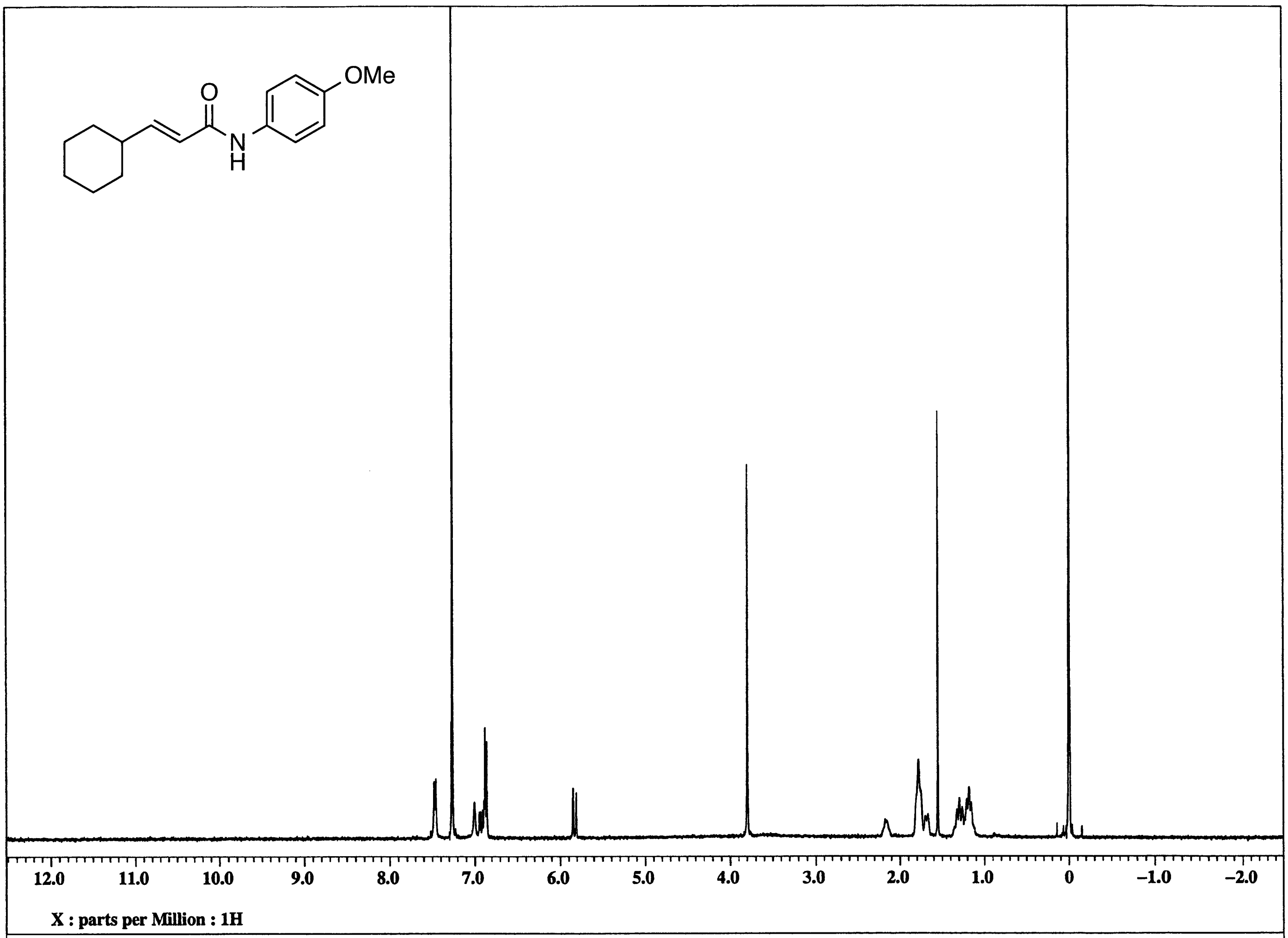
OMe
O
N
H
12.0 11.0 10.0 9.0 8.0 7.0 6.0 5.0 4.0 3.0 2.0 1.0 0 −1.0 −2.0
X : parts per Million : 1H

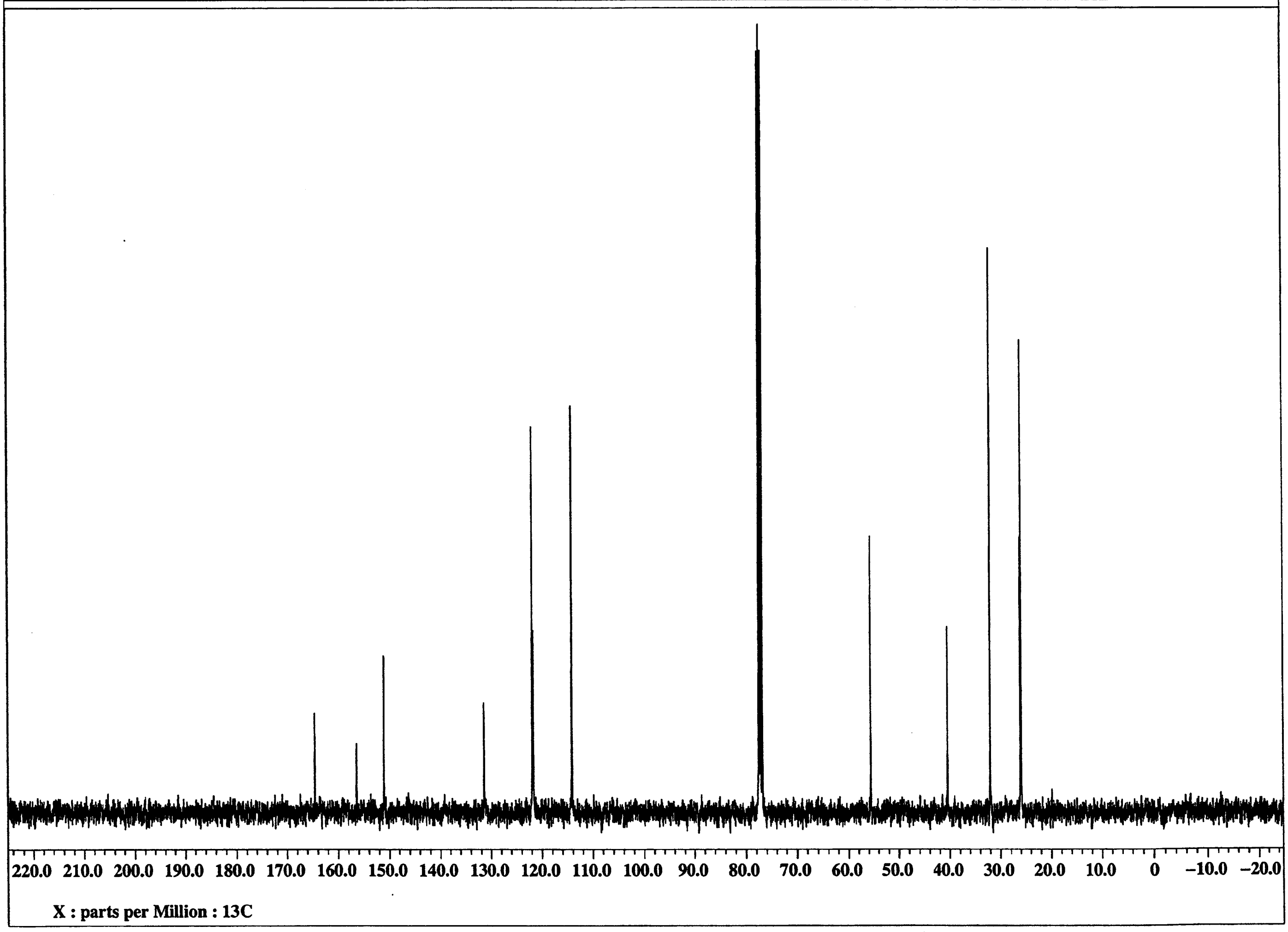
220.0 210.0 200.0 190.0 180.0 170.0 160.0 150.0 140.0 130.0 120.0 110.0 100.0 90.0 80.0 70.0 60.0 50.0 40.0 30.0 20.0 10.0 0 −10.0 −20.0
X : parts per Million : 13C

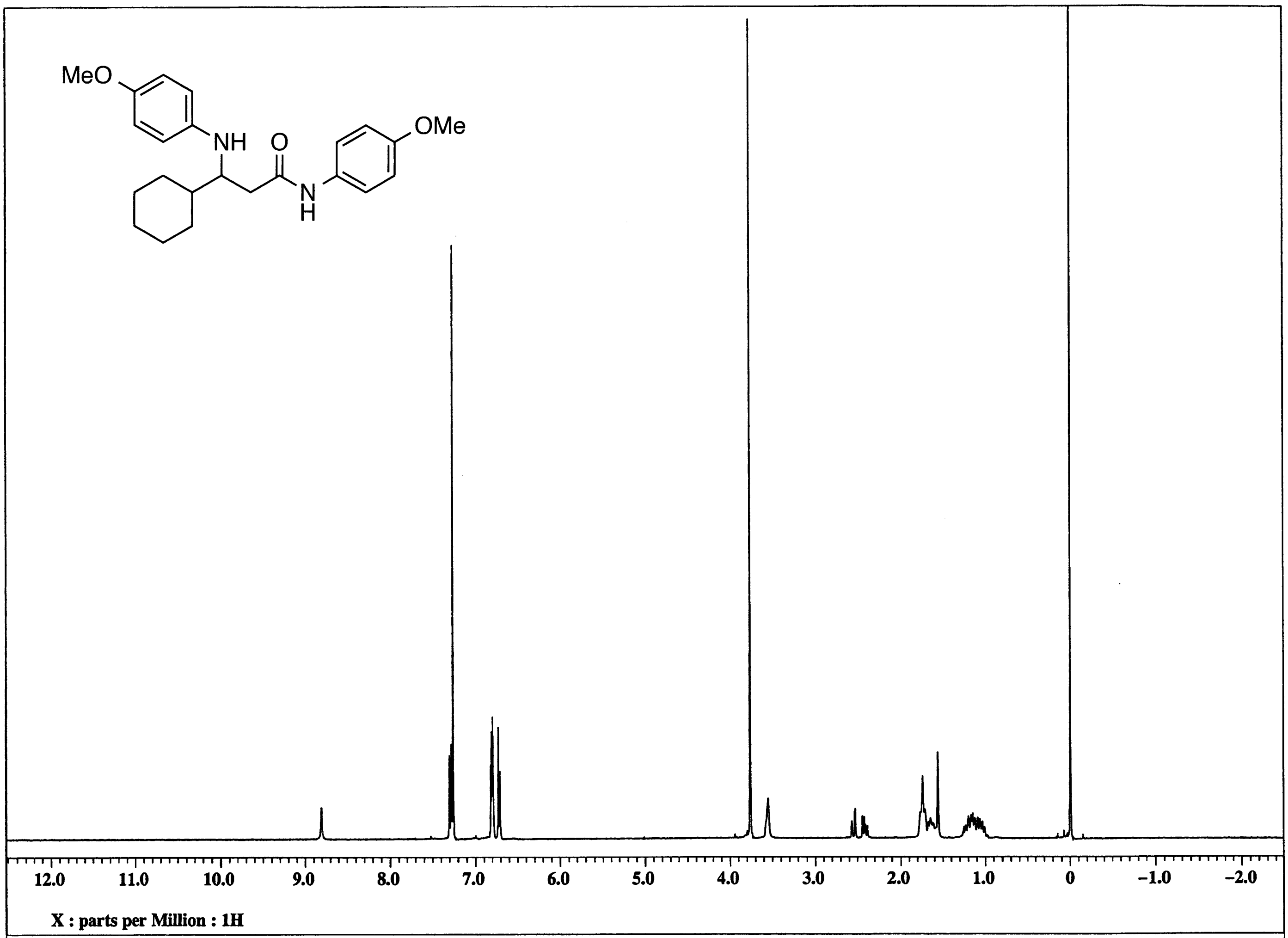
MeO
NH
O
OMe
N
H
12.0 11.0 10.0 9.0 8.0 7.0 6.0 5.0 4.0 3.0 2.0 1.0 0 −1.0 −2.0
X : parts per Million : 1H

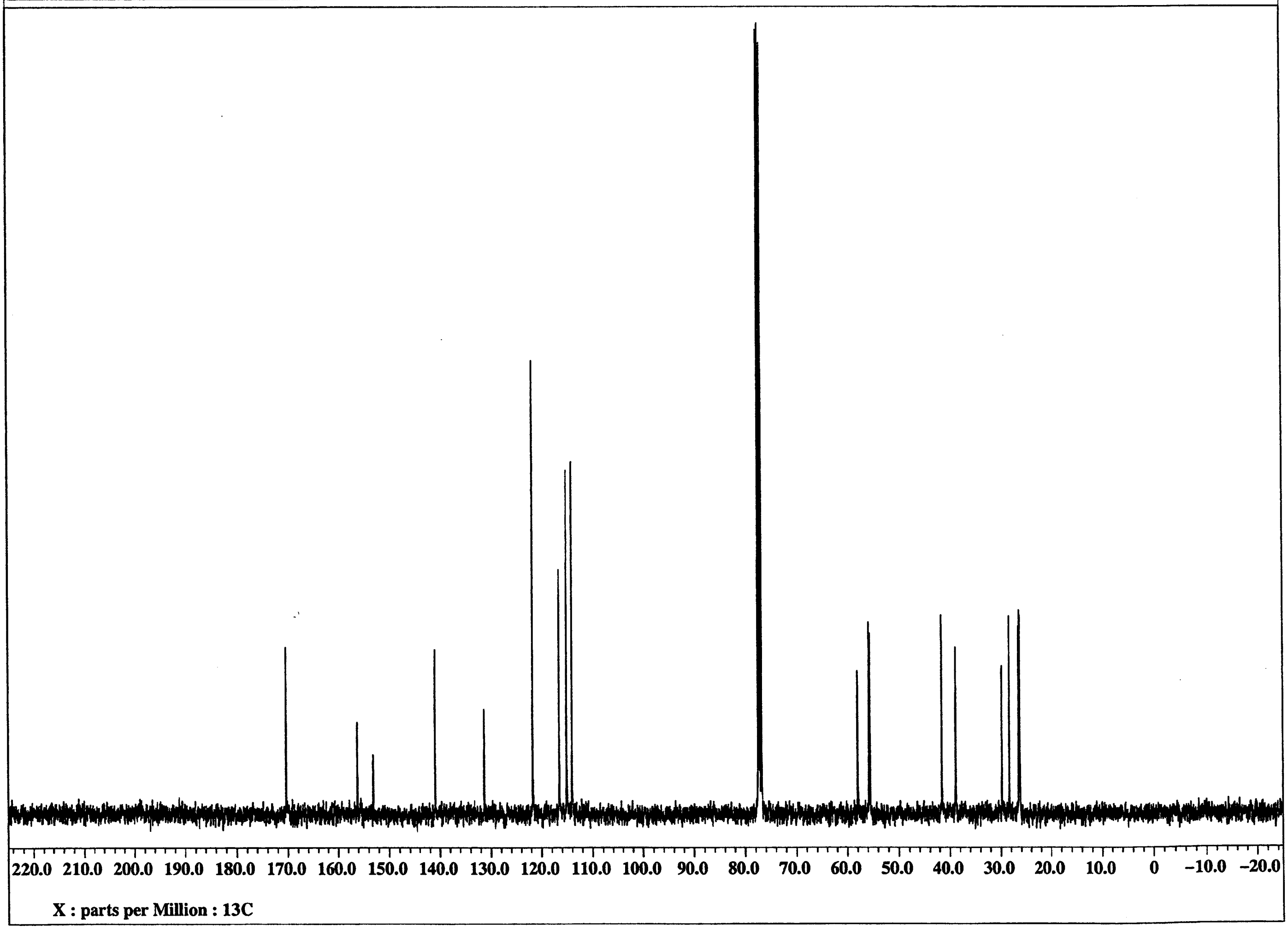
220.0 210.0 200.0 190.0 180.0 170.0 160.0 150.0 140.0 130.0 120.0 110.0 100.0 90.0 80.0 70.0 60.0 50.0 40.0 30.0 20.0 10.0 0 −10.0 −20.0
X : parts per Million : 13C

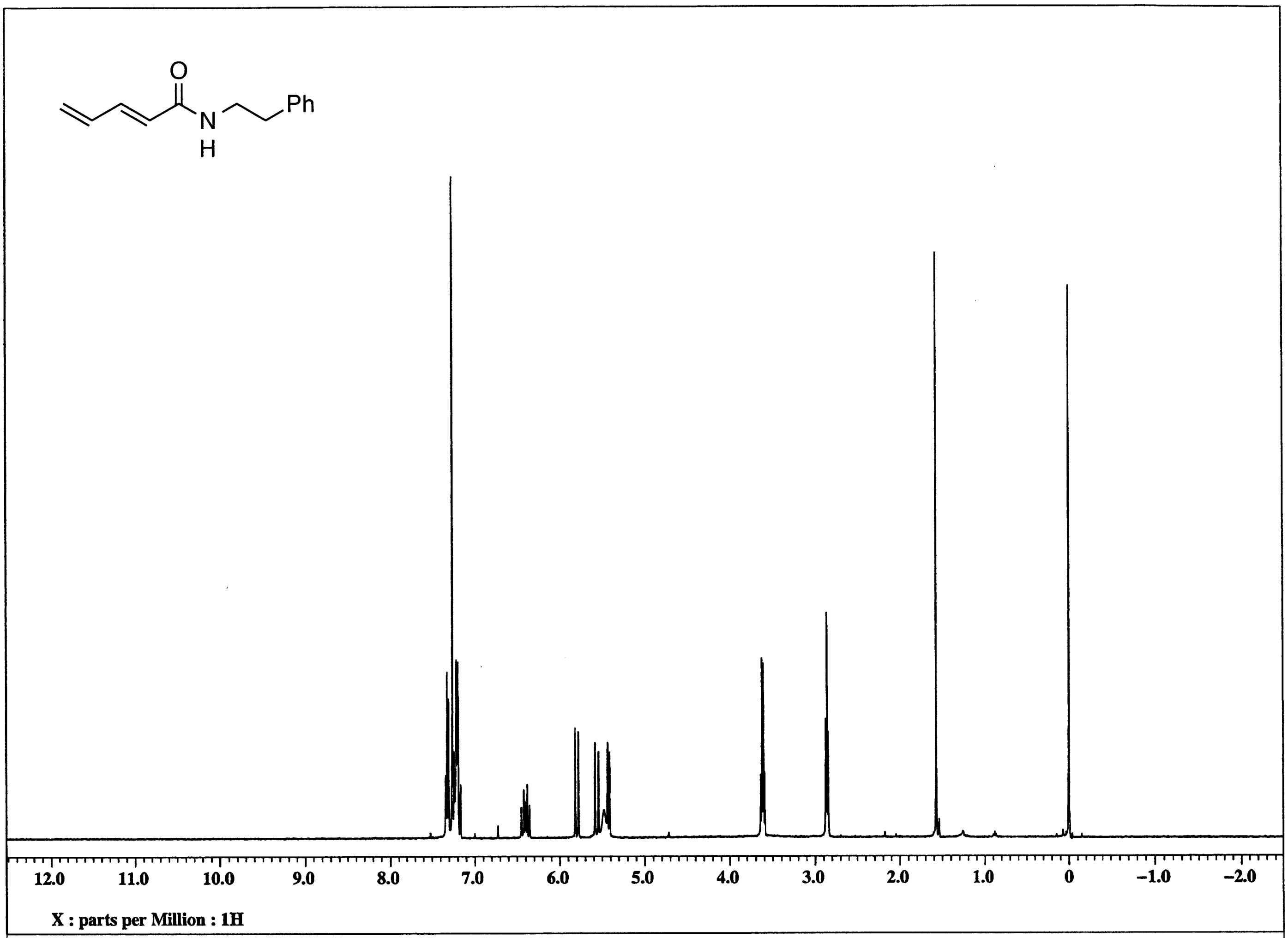
O
N
H
Ph
12.0 11.0 10.0 9.0 8.0 7.0 6.0 5.0 4.0 3.0 2.0 1.0 0 −1.0 −2.0
X : parts per Million : 1H

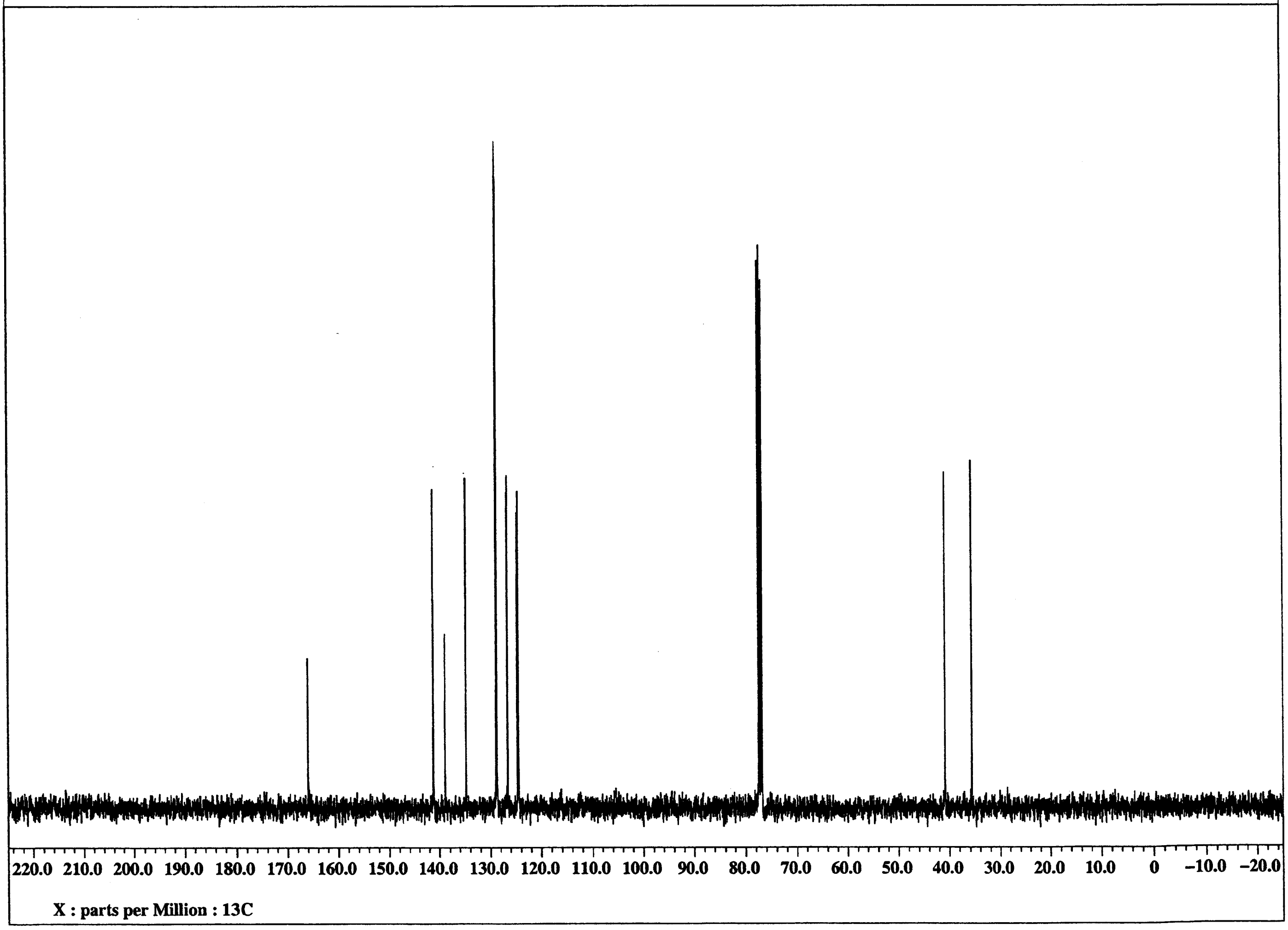
220.0 210.0 200.0 190.0 180.0 170.0 160.0 150.0 140.0 130.0 120.0 110.0 100.0 90.0 80.0 70.0 60.0 50.0 40.0 30.0 20.0 10.0 0 −10.0 −20.0
X : parts per Million : 13C

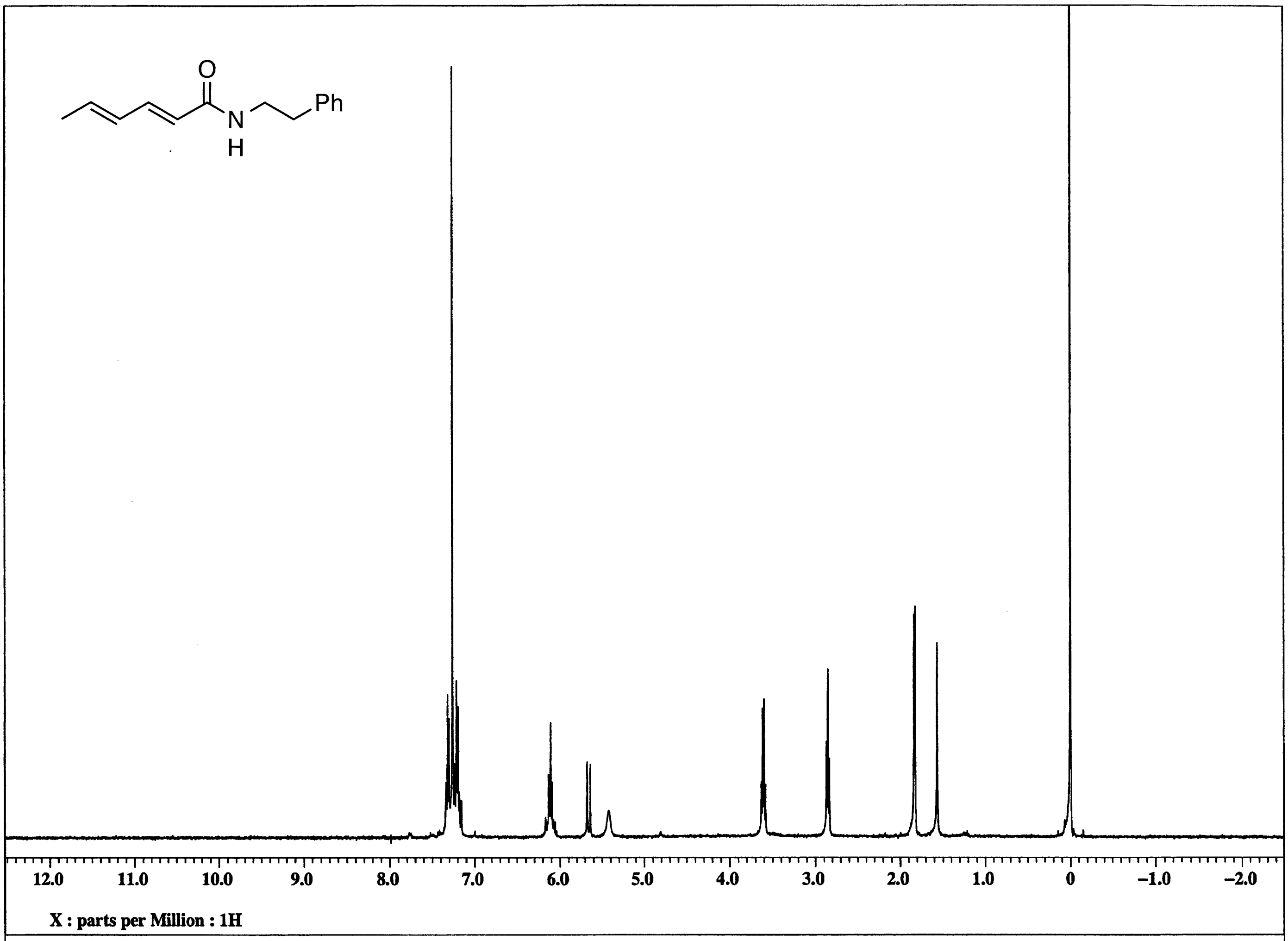
O
N
H
Ph
12.0 11.0 10.0 9.0 8.0 7.0 6.0 5.0 4.0 3.0 2.0 1.0 0 −1.0 −2.0
X : parts per Million : 1H

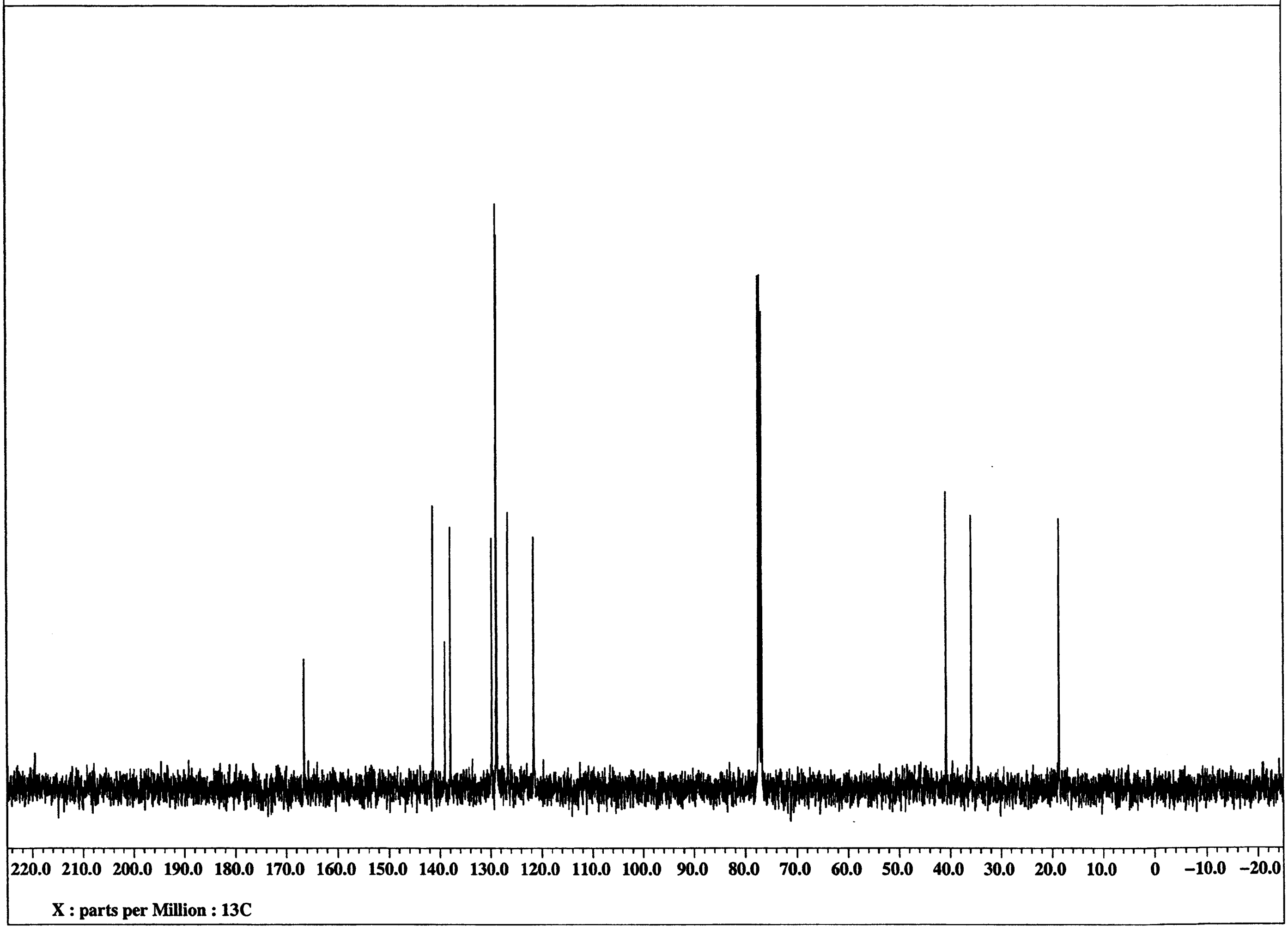
220.0 210.0 200.0 190.0 180.0 170.0 160.0 150.0 140.0 130.0 120.0 110.0 100.0 90.0 80.0 70.0 60.0 50.0 40.0 30.0 20.0 10.0 0 −10.0 −20.0
X : parts per Million : 13C

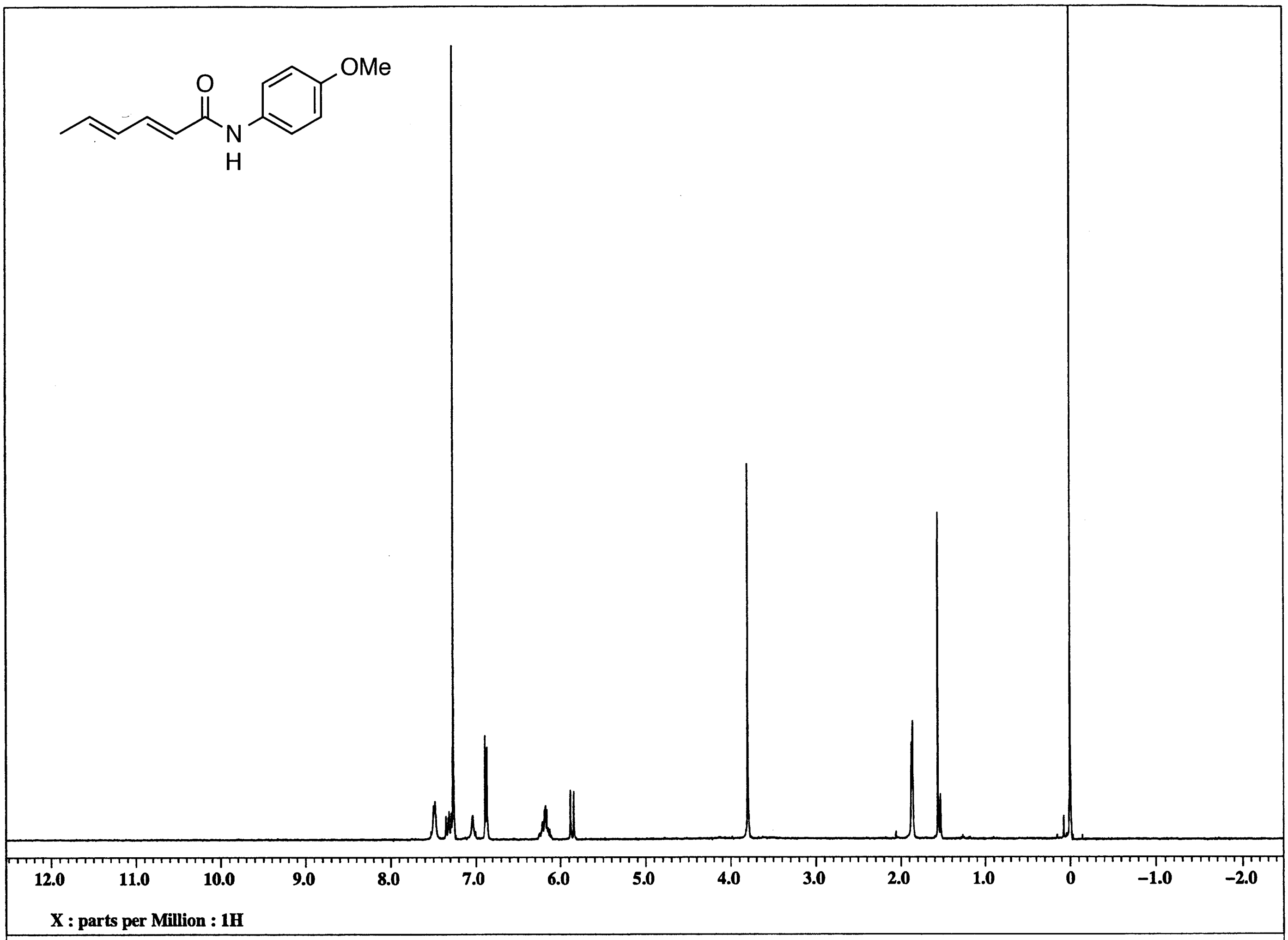
OMe
O
N
H
12.0 11.0 10.0 9.0 8.0 7.0 6.0 5.0 4.0 3.0 2.0 1.0 0 −1.0 −2.0
X : parts per Million : 1H

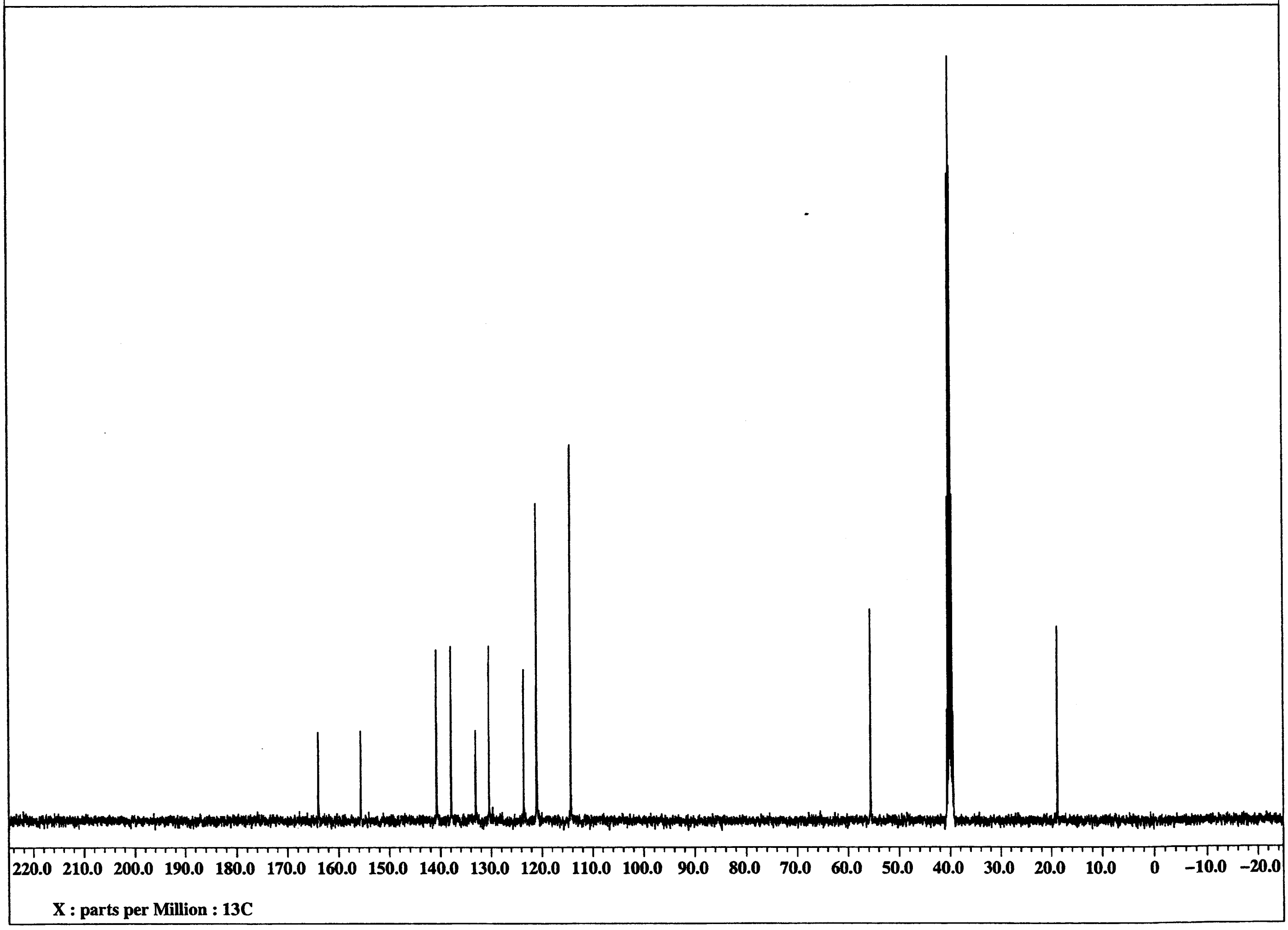
220.0 210.0 200.0 190.0 180.0 170.0 160.0 150.0 140.0 130.0 120.0 110.0 100.0 90.0 80.0 70.0 60.0 50.0 40.0 30.0 20.0 10.0 0 −10.0 −20.0
X : parts per Million : 13C

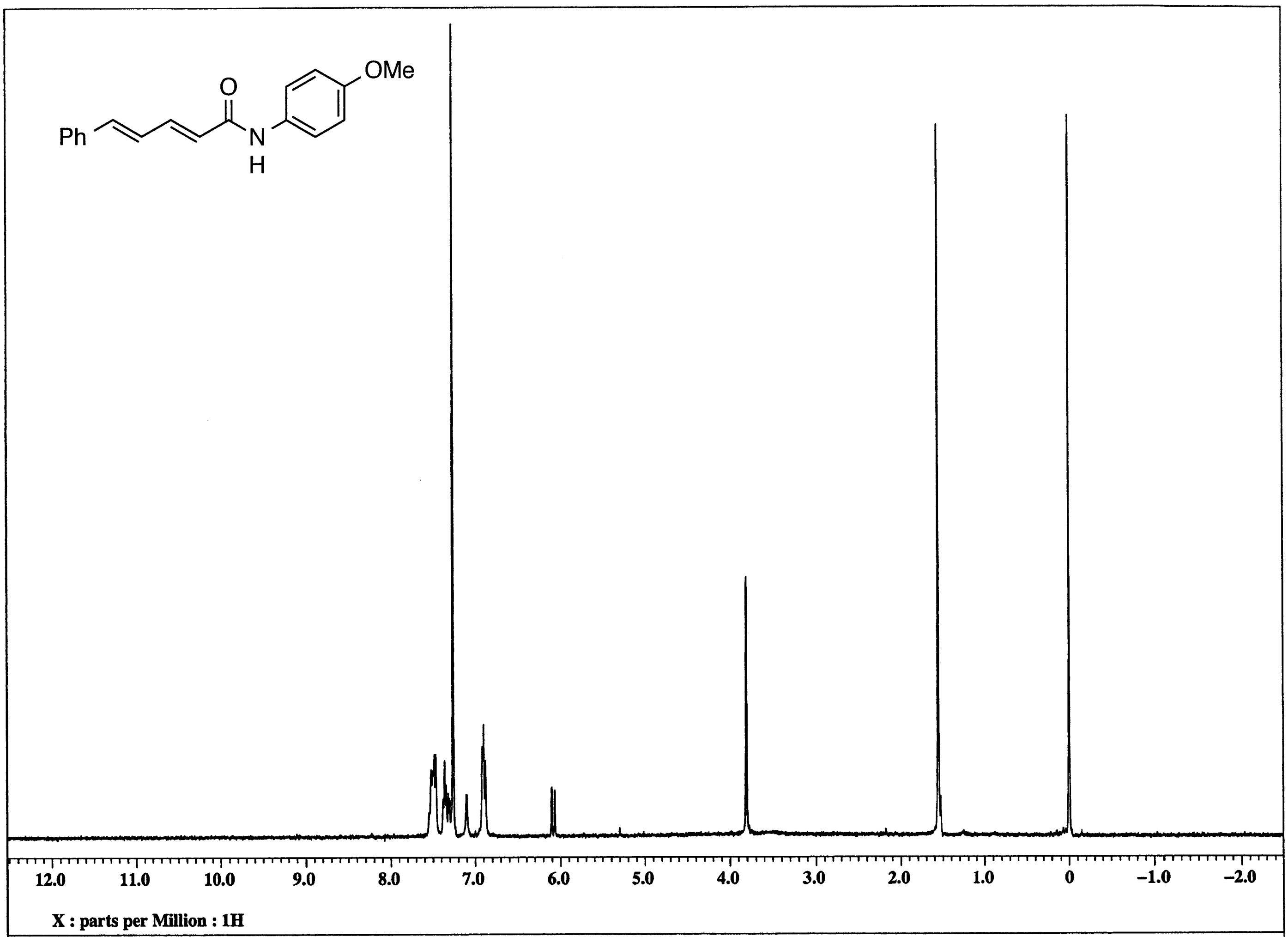
OMe
O
Ph
N
H
12.0 11.0 10.0 9.0 8.0 7.0 6.0 5.0 4.0 3.0 2.0 1.0 0 −1.0 −2.0
X : parts per Million : 1H

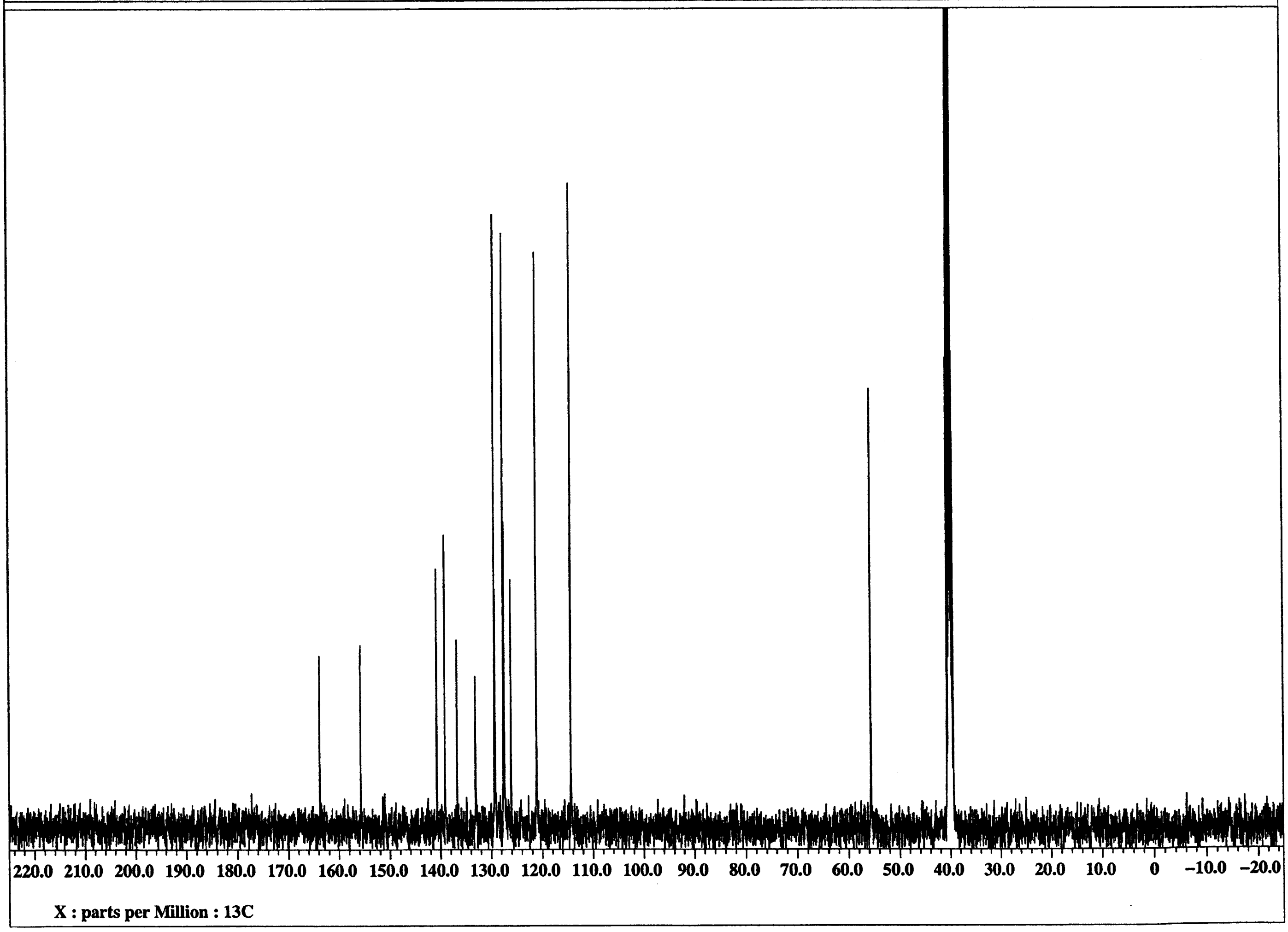
220.0 210.0 200.0 190.0 180.0 170.0 160.0 150.0 140.0 130.0 120.0 110.0 100.0 90.0 80.0 70.0 60.0 50.0 40.0 30.0 20.0 10.0 0 −10.0 −20.0
X : parts per Million : 13C

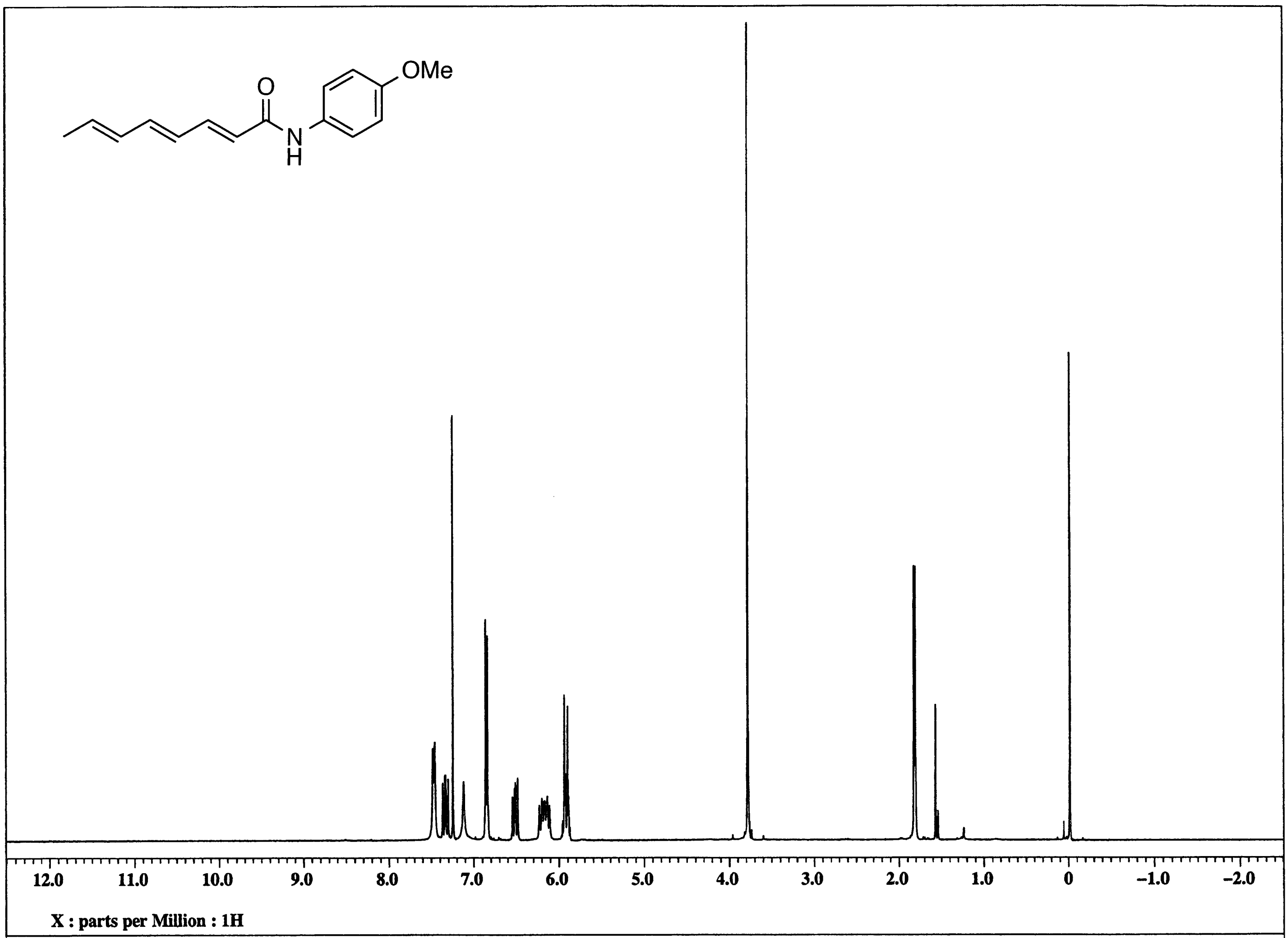

OMe
O
N
H
12.0 11.0 10.0 9.0 8.0 7.0 6.0 5.0 4.0 3.0 2.0 1.0 0 −1.0 −2.0
X : parts per Million : 1H

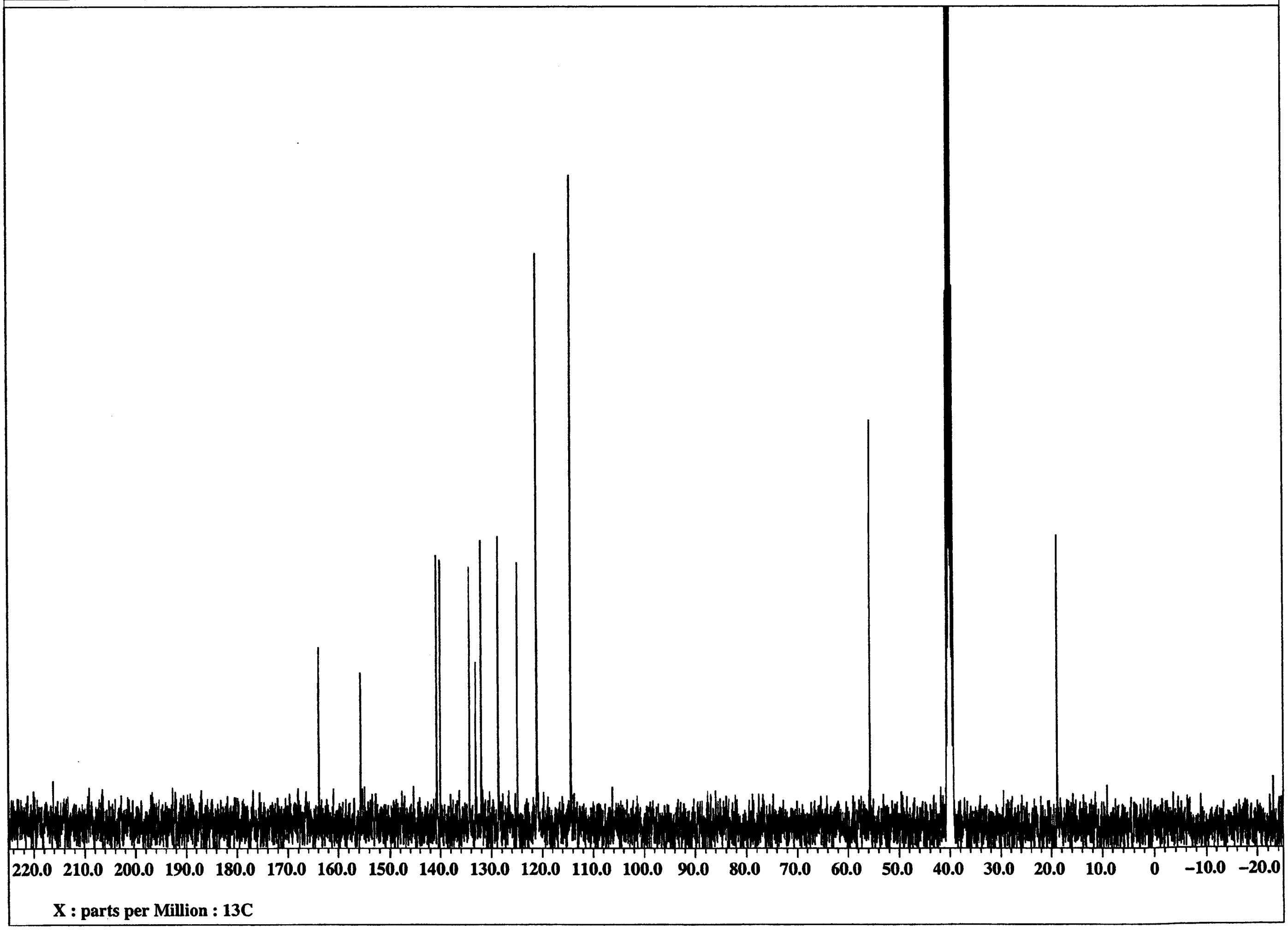

220.0 210.0 200.0 190.0 180.0 170.0 160.0 150.0 140.0 130.0 120.0 110.0 100.0 90.0 80.0 70.0 60.0 50.0 40.0 30.0 20.0 10.0 0 −10.0 −20.0
X : parts per Million : 13C

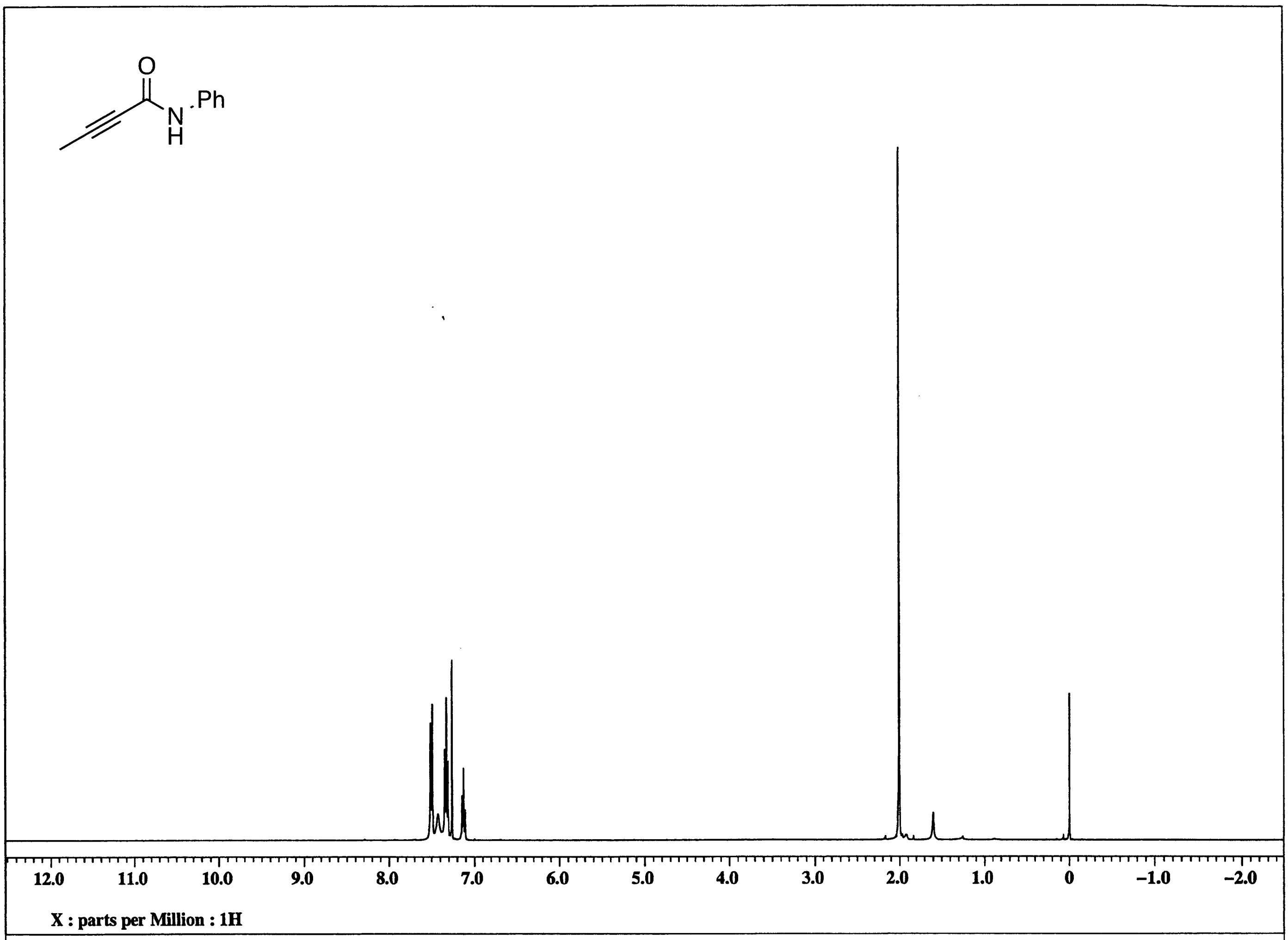

O
N
Ph
H
12.0 11.0 10.0 9.0 8.0 7.0 6.0 5.0 4.0 3.0 2.0 1.0 0 −1.0 −2.0
X : parts per Million : 1H

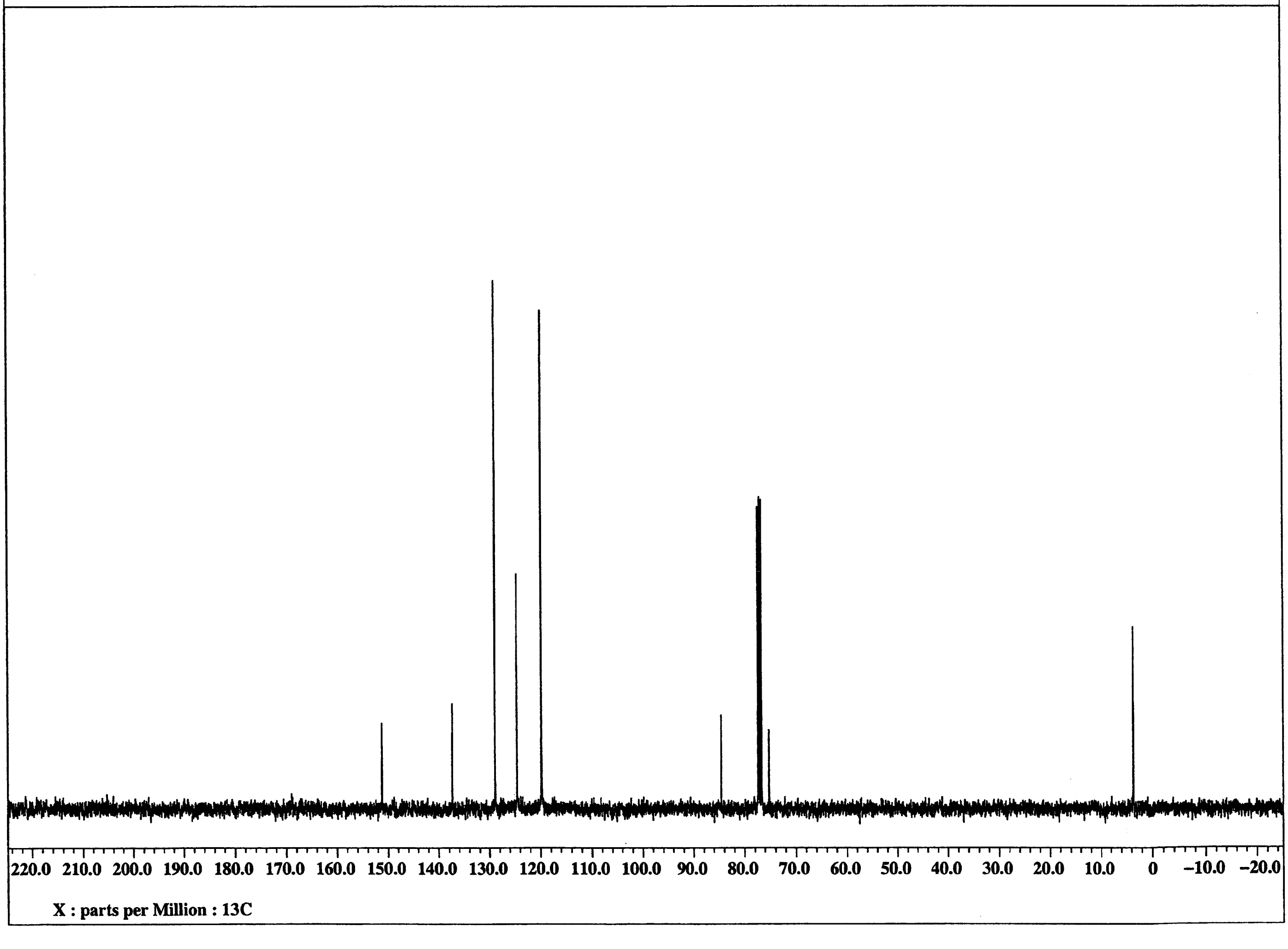

220.0 210.0 200.0 190.0 180.0 170.0 160.0 150.0 140.0 130.0 120.0 110.0 100.0 90.0 80.0 70.0 60.0 50.0 40.0 30.0 20.0 10.0 0 −10.0 −20.0
X : parts per Million : 13C

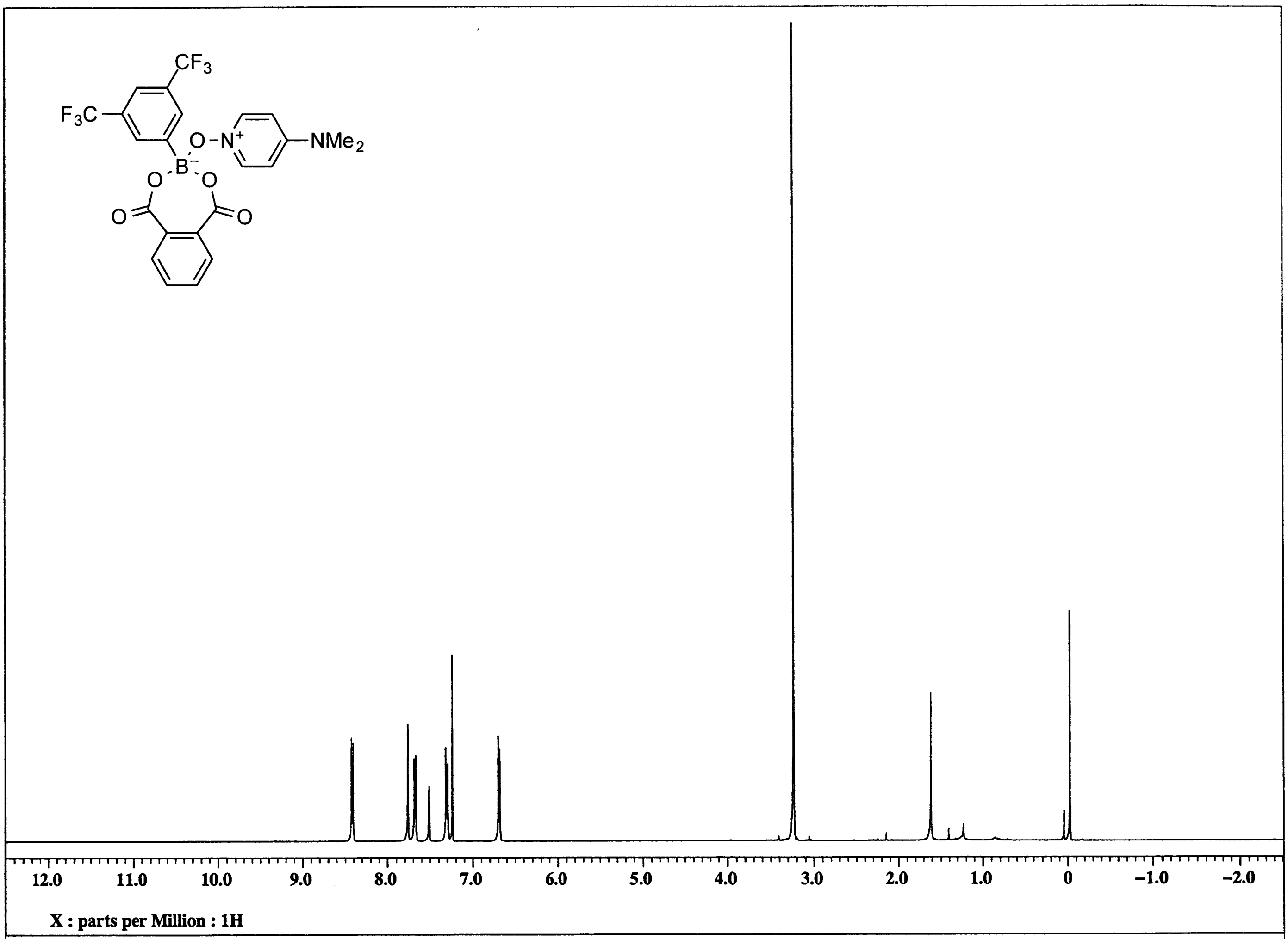
CF3
F3C
O
N+
NMe2
B−
O
O
O
O
12.0
11.0
10.0
9.0
8.0
7.0
6.0
5.0
4.0
3.0
2.0
1.0
0
−1.0
−2.0
X : parts per Million : 1H

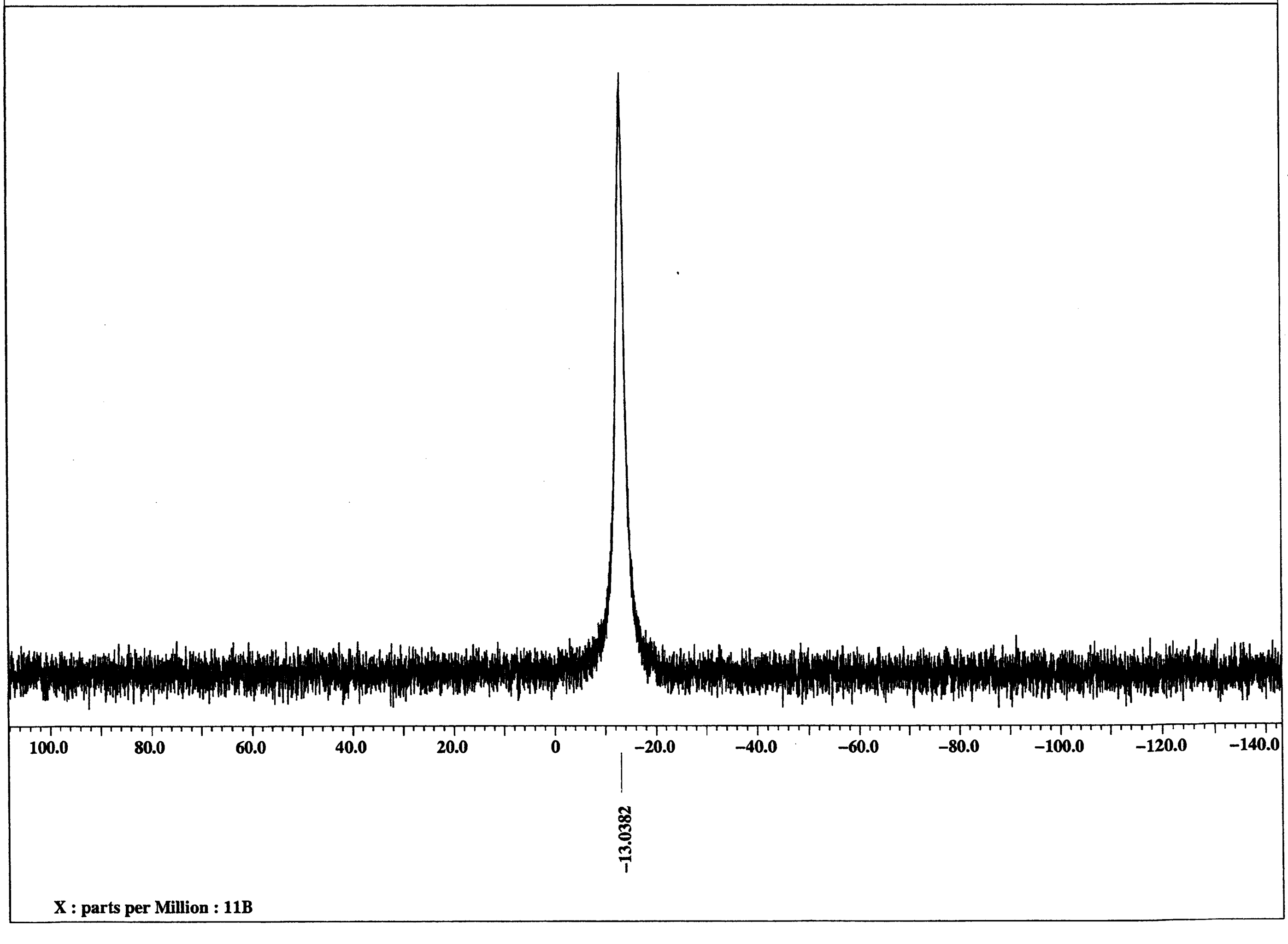
100.0
80.0
60.0
40.0
20.0
0
−20.0
−40.0
−60.0
−80.0
−100.0
−120.0
−140.0
−13.0382
X : parts per Million : 11B

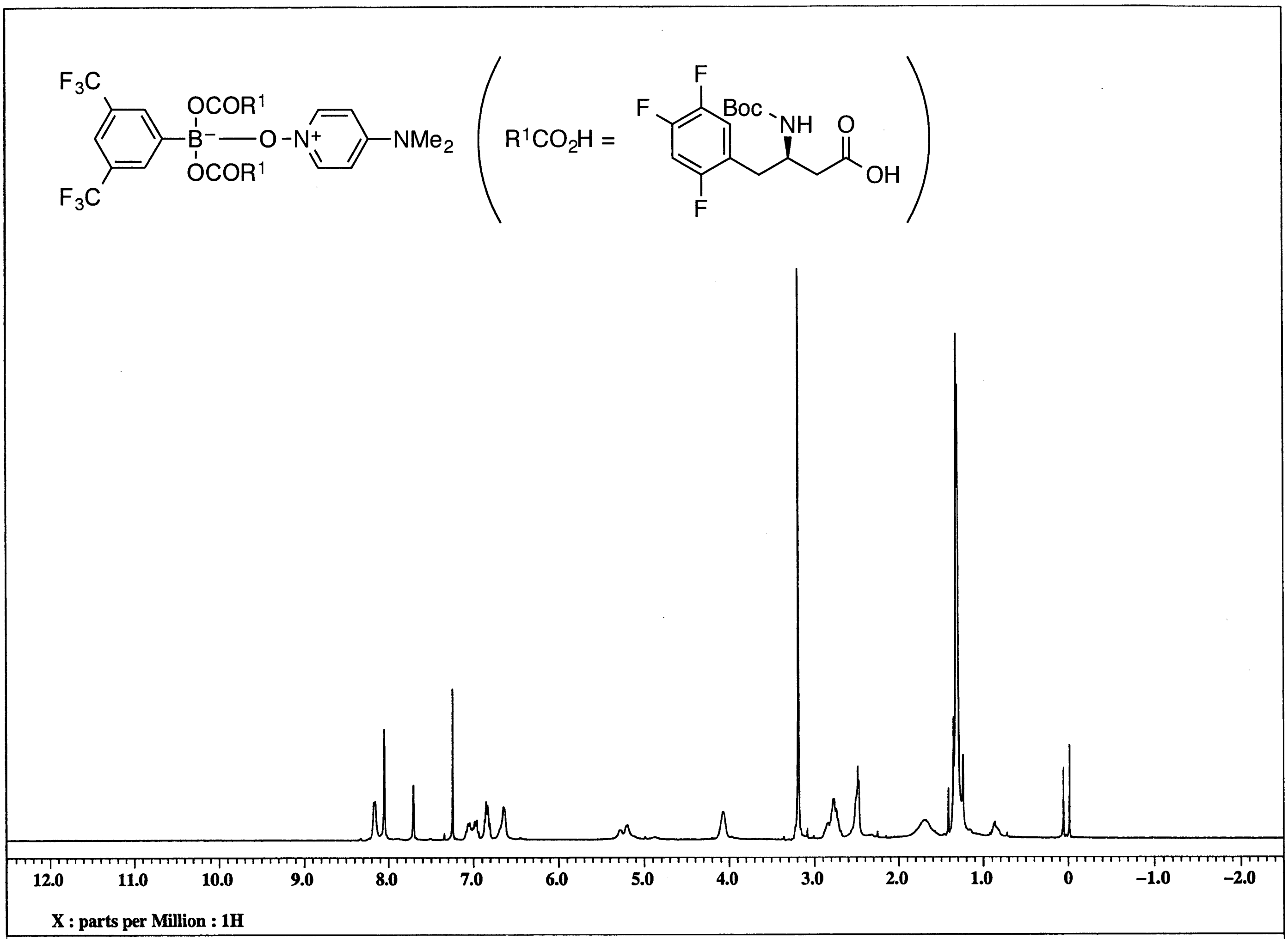
F3C
OCOR1
B−
O
N+
NMe2
OCOR1
F3C
R1CO2H =
F
F
Boc
NH
O
OH
F
12.0
11.0
10.0
9.0
8.0
7.0
6.0
5.0
4.0
3.0
2.0
1.0
0
−1.0
−2.0
X : parts per Million : 1H

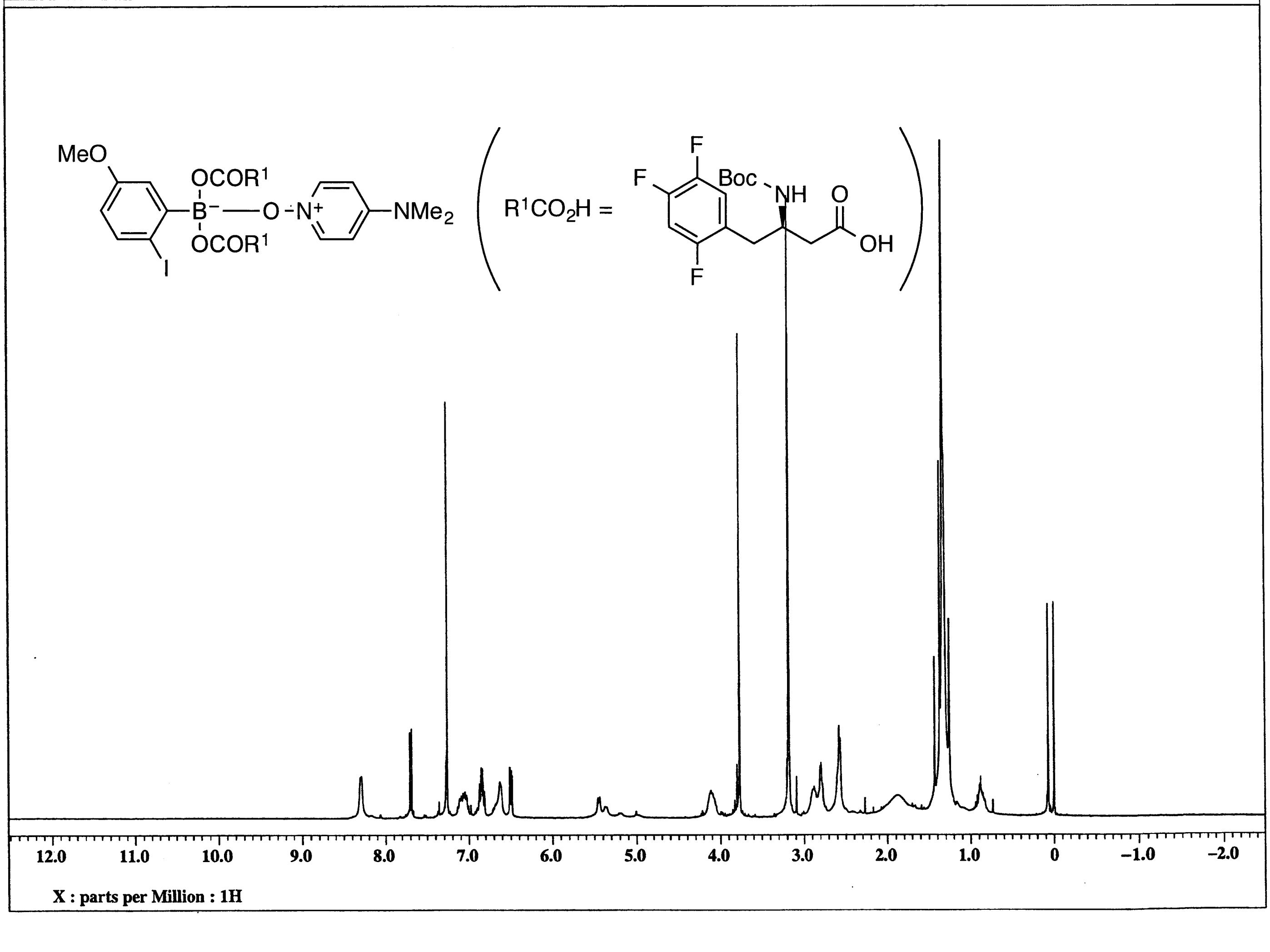
MeO
OCOR1
B−
O
N+
NMe2
OCOR1
I
R1CO2H =
F
F
Boc
NH
O
OH
F
12.0
11.0
10.0
9.0
8.0
7.0
6.0
5.0
4.0
3.0
2.0
1.0
0
−1.0
−2.0
X : parts per Million : 1H

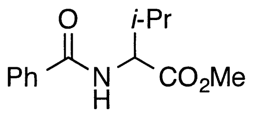

Methyl Benzoyl-*D,L*-valinate

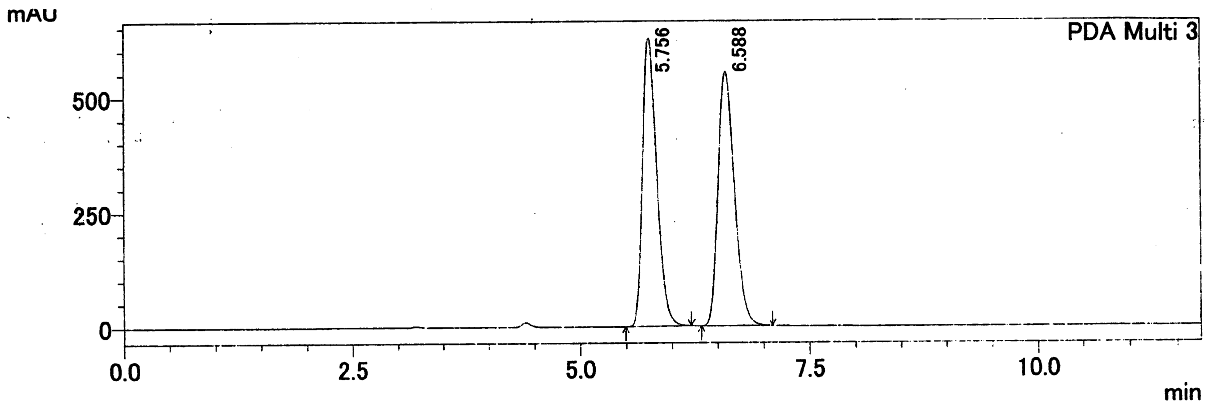

1 PDA Multi 3/225nm 4nm

ｽﾍﾟｸﾄﾙ

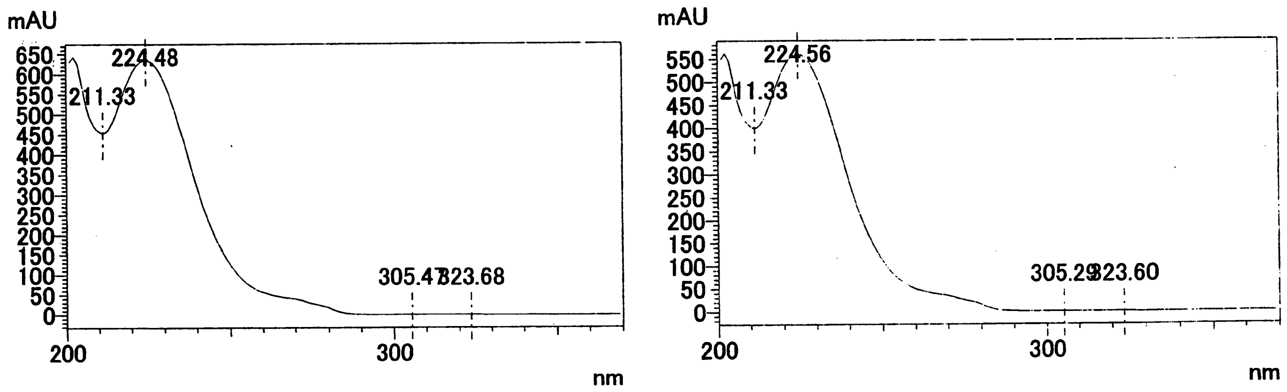

ﾋﾟｰｸﾃｰﾌﾞﾙ

PDA Ch3 225nm 4nm

ﾋﾟｰｸ#	化合物名	保持時間	最低純度点ﾋﾟｭﾘﾃｨ指数	高さ	面積	面積%
1		5.756	31	627252	6785696	50.025
2		6.588	31	553572	6779044	49.975
合計				1180824	13564740	100.000

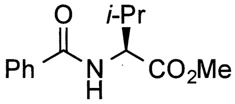

Methyl Benzoyl-*L*-valinate

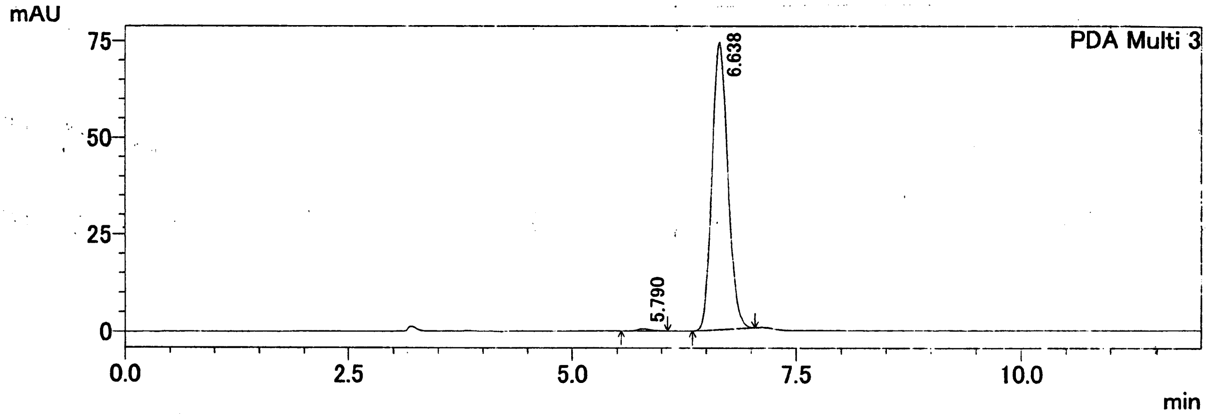

1 PDA Multi 3/225nm 4nm

ｽﾍﾟｸﾄﾙ

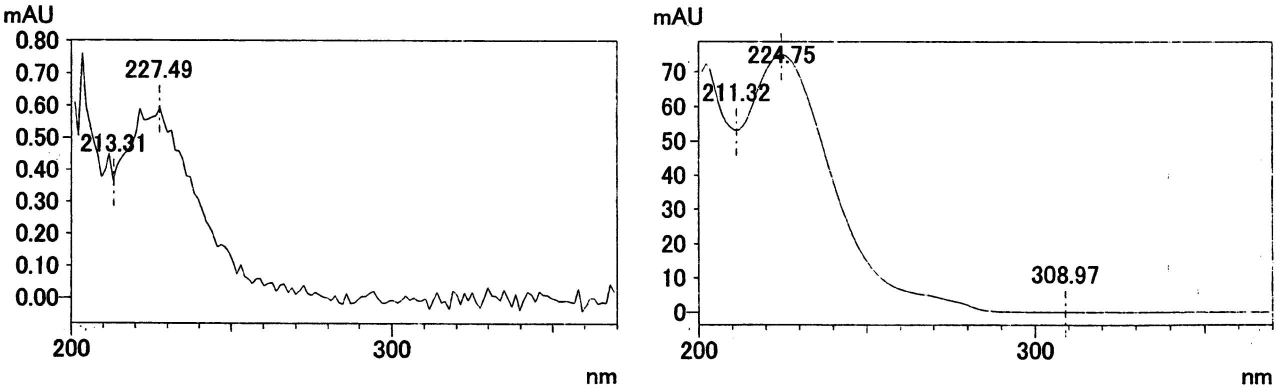

ﾋﾟｰｸﾃｰﾌﾞﾙ

PDA Ch3 225nm 4nm

ﾋﾟｰｸ#	化合物名	保持時間	最低純度点ﾋﾟｭﾘﾃｨ指数	高さ	面積	面積%
1		5.790	Cannot be calculated	572	6418	0.708
2		6.638	436	74064	900450	99.292
合計				74636	906868	100.000
